# Mild and Selective Hydrogenation of Unsaturated Compounds Using Mn/Water as a Hydrogen Gas Source

**DOI:** 10.1021/acs.orglett.3c03664

**Published:** 2023-12-14

**Authors:** Jennifer Rosales, Tania Jiménez, Rachid Chahboun, Miguel A. Huertos, Alba Millán, José Justicia

**Affiliations:** † Department of Organic Chemistry, Faculty of Sciences, University of Granada, C. U. Fuentenueva s/n, 18071 Granada, Spain; ‡ University of Basque Country (UPV/EHU), 20018 Donostia-San Sebastian, Spain; § IKERBASQUE, Basque Foundation for Science, 48013 Bilbao, Spain

## Abstract

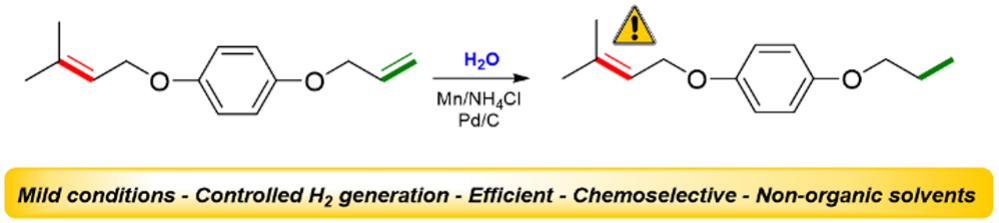

A mild and highly selective reduction of alkenes and alkynes using Mn/water is described. The highly controlled generation of H_2_ allows the selective reduction of these compounds in the presence of labile functional groups under mild and environmentally acceptable conditions.

The hydrogenation reaction is the addition of hydrogen atoms to multiple C–C bonds, C–heteroatom bonds, and others (Scheme 1A).^1^ Such reactions have been widely used for the production of compounds worldwide, from large-scale operations and important industrial processes to the synthesis of fine chemicals.^2^ This process is mediated by either homogeneous or heterogeneous transition-metal-based catalysts, used in large amounts (especially under homogeneous conditions).^3,4^ Recently, metal-free methodologies based on frustrated Lewis pairs (FLPs) have been described.^5^ Despite that H_2_ is the cleanest and most efficient reducing agent, it is an extremely flammable gas that requires special and costly materials for storage and reactions.^6^ To avoid these issues, other reagents, such as silanes, amines, ammonia-borane, alcohols, and strong acids, are also used as H atom sources.^7^ However, these reagents are toxic and require the use of organic solvents on a large scale, which have associated environmental disadvantages. Moreover, the lack of selectivity in general procedures requires the use of nonsimple catalysts.^3,8−10^

**Scheme 1:**
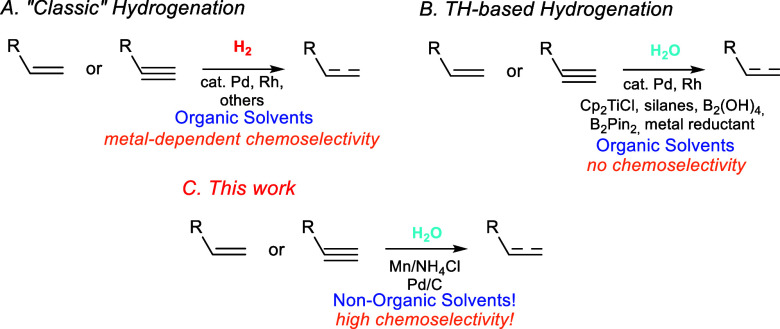
Methods for Hydrogenation of Multiple C–C Bonds

An alternative is catalytic transfer hydrogenation (TH) reactions, which uses eco-friendly sources of hydrogen atoms, such as water (Scheme 1B). Thus, from the pioneering works described by Inoue, Oltra, and Cuerva based on the use of an Rh-catalyzed transfer of H atoms from H_2_O^11a^ and a Cp_2_TiCl/H_2_O system,^11b^ respectively, several strategies of TH using amounts of water, transition-metal catalysts (Pd, Ni, Rh, Ru, or Co), metallic or metalloid reagents (Zn dust or B), in organic solvents, have been described.^12−14^ However, similar drawbacks can be found related to high-cost metal catalyst and/or reagents and the use of organic solvents together with the lack of selectivity in the reduction of alkenes.^12,15^ Thus, alternative processes for the selective hydrogenation of multiple C–C bonds using simple, cheap, and environmentally acceptable conditions are desirable.

Herein, we report a method for the efficient and highly selective hydrogenation of alkenes and alkynes using a combination of water and Mn dust to generate H_2_ gas *in situ* under low and controlled pressure. Previously, we described a highly chemoselective reduction of aldehydes to alcohols using these reagents, and a mechanism based on hydrogen atom generation from water promoted by “activated” Mn dust was proposed.^16^ During the experiments, we detected a slight overpressure in the reaction, possibly due to H_2_ generation. To check this hypothesis, we applied the developed conditions^16^ to the reduction of alkene **1a** using Pd/C as catalyst and tap water as solvent. To our delight, we obtained compound **2a** in quantitative yields, which confirmed the hypothesis (Scheme 2).

**Scheme 2:**
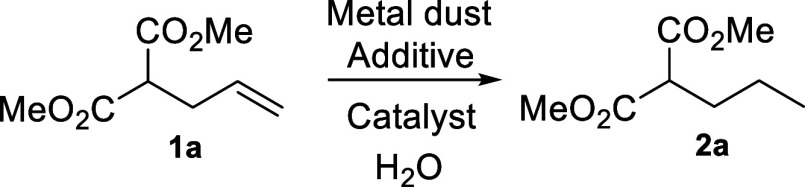
Reduction of **1a** Using a Metal Dust/H_2_O System

We explored the general conditions extensively using accessible metal dust (such as Ni, Fe, Zn, Al, Mg, and Mn), hydrochloric salts as additives, hydrogenation catalysts, and water as solvent for the reduction of **1a**.^17^ After several experiments, we confirmed that the best reagent combination was Mn dust, 5% Pd/C as catalyst, and water as both hydrogen atoms source and solvent.^18^ It is worth mentioning that NH_4_Cl, 2,4,6-collidine·HCl, 2,6-lutidine·HCl, and pyridine·HCl were also appropriate, although pyridine·HCl yielded the best results with all metals. However, its acidic character (p*K*_a_ = 5.23) could affect acid-labile functional groups, such as epoxides or esters, decreasing the chemoselectivity of the reaction. To avoid that and contribute to the environmentally acceptable character of this reaction,^19^ inexpensive, inorganic, and less acidic NH_4_Cl (p*K*_a_ = 9.25), which yields excellent results with Mn dust, was selected. These reagents provided soft and slightly basic conditions (pH = 9.2) for our reactions. Regarding the other tested metals, only Zn with pyridine·HCl performed the reaction with a good yield.^17^ These results indicate that the reaction is not related to the reduction potential (*E*^0^) because metals with higher *E*^0^ than Mn (Al or Mg) are unable to promote hydrogenation.

Once the best experimental conditions were determined,^17^ we applied them for the reduction of alkenes **1a**–**v**, **3a**–**f**, and **5a**, with different functional and protective groups. The results are depicted in Schemes 3 and 4.

**Scheme 3:**
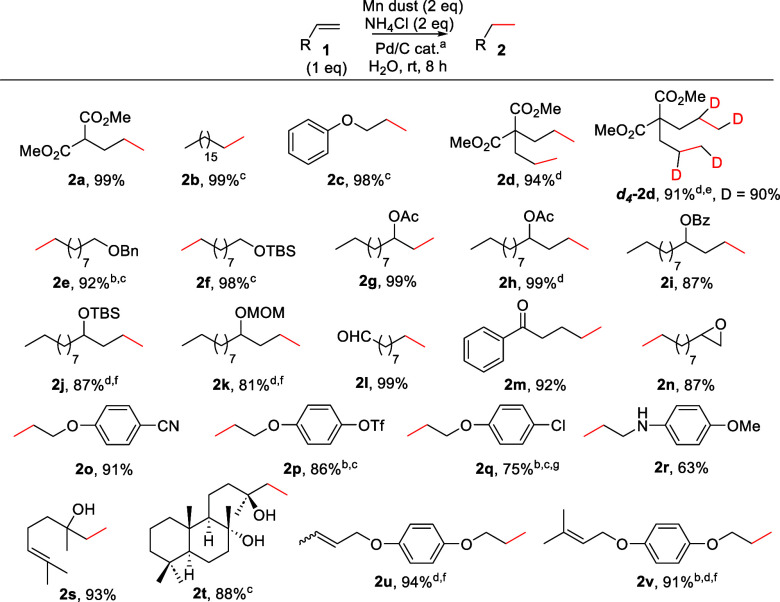
Hydrogenation of Alkenes **1a**–**v**^20^ 5% Pd/C (0.63–1.47 mol % Pd).^17^ Isolated yield after flash column chromatography purification. THF:H_2_O 1:4. 24 h. D_2_O and ND_4_Cl. THF:H_2_O 1:1. 17% of dehalogenation product.

Our reaction worked perfectly with different alkenes, yielding the corresponding alkanes in high or quantitative yields. Consequently, in most cases, no chromatographic purification was required for the isolation of pure compounds. The mild and controlled conditions were compatible with several functional groups, including those labile to the common hydrogenation reactions and/or transition metals and acidic media. Thus, alkenes in the presence of carbonyl groups (**1l**, **1m**), esters (**1a**, **1d**, **3a**, **3d**), and aromatic rings (**1c**, **1o**–**r**, **1u**–**v**, **3c**–**f**), susceptible to hydrogenation under determined conditions,^21,22^ were reduced chemoselectively to the corresponding alkanes. Commonly used protective groups, such as silyl ethers (**1f**, **1j**), benzoates (**1i**), methoxymethyl ethers (MOM) (**1k**), and acetates (**1g**, **1h**), were not affected under our hydrogenation conditions. In addition, functional groups prone to oxidative addition by Pd catalysts, such as triflates (**1p**) or halogens (**1q**), were also compatible. Interestingly, benzyl groups, normally removed by heterogeneous hydrogenations,^23^ were also stable and allowed the chemoselective reduction of the corresponding alkene **1e**. Our mild and nonacidic conditions allow the reduction of substrates containing acid labile epoxide (**2n**). Remarkably, an unusually high selectivity in the hydrogenation of less substituted alkenes in the presence of more substituted alkenes was observed. This selectivity is a consequence of the slower reaction rate observed for the latter compounds together with the controlled generation of H_2_. Thus, alkenes **1u**, **1v**, and **3f** were reduced to **2u**, **2v**, and **4f** in high yields and complete selectivity. These results are not possible using common conditions in heterogeneous hydrogenation. This selectivity is even greater than that of homogeneous Wilkinson’s catalyst,^24^ using cheaper and easier conditions. Additionally, we prepared deuterated compounds ***d***_**4**_**-2d** and ***d***_**2**_**-4c** in high yield and isotopic incorporation (90 and 81%, respectively) using D_2_O as solvent and ND_4_Cl as additive. These results confirmed that the incoming hydrogen atoms came from water, considering that no deuteration was observed when ND_4_Cl was used as the only deuterium source.

No reaction was observed when trisubstituted alkenes were checked.^17^ This fact matched with the previously observed reactivity (Schemes 3 and 4), providing more evidence of the high selectivity of our proposal. Surprisingly, compound **5a** yielded a selective reduction of the tetrasubstituted alkene (**6a**). This result could be due to the higher reactivity of this alkene due to the ring strain in this compound, with the release of tension being the driving force of the reaction. The *cis*-relative stereochemistry of **6a** was confirmed by NOESY and 2D NMR experiments.^17^

**Scheme 4:**
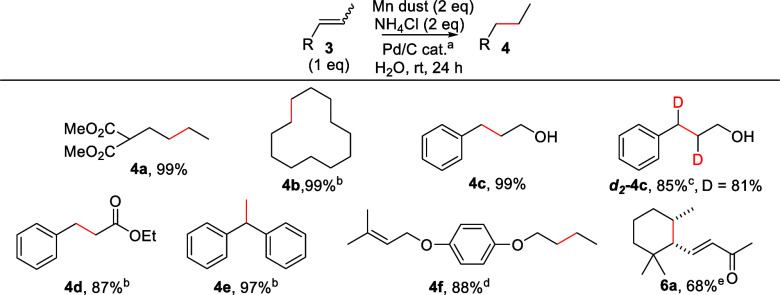
Hydrogenation of Alkenes **3a**–**f** and **5a**(20) 5% Pd/C (0.63–1.09 mol % Pd).^17^ THF:H_2_O 1:4. D_2_O and ND_4_Cl were used. 4 equiv of Mn dust and NH_4_Cl, THF:H_2_O 1:1, and 30 h. 14% of complete reduction product (**6a-*red***) was observed.

The semihydrogenation of alkynes to alkenes is also an important process in organic chemistry. This reaction has been applied to the preparation of useful building blocks for the synthesis of high-value chemicals or natural products,^25^ which include a double bond in defined (*E*) or (*Z*) configuration.^26^ Semihydrogenation to give (*Z*)-alkenes is usually performed under heterogeneous conditions using Lindlar’s catalyst as the main reagent. Recently, TH strategies have been described,^12−14,27^ introducing an alternative to the classic protocol. However, the use of considerable amounts of organic solvents and specific and structurally complex catalysts limits its application.

We extended our procedure to the partial and selective hydrogenation of alkynes **7a**–**i** to (*Z*)-alkenes **1a** and **8b**–**i**, using Lindlar’s catalyst to promote the reaction. The results are depicted in Scheme 5.

**Scheme 5:**
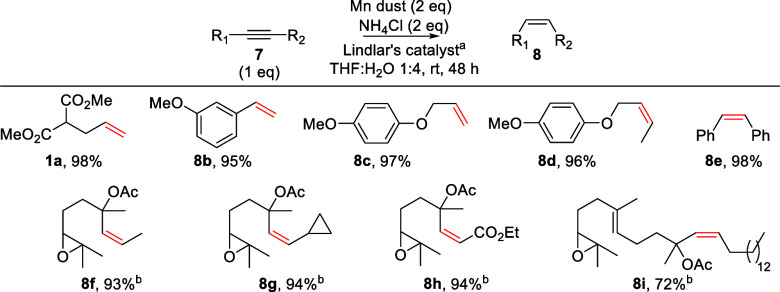
Semi-Hydrogenation of Alkynes **7a**–**i**^20^ 5% Lindlar (0.65–1.48 mol % Pd). 76 h.

Complex alkynes were efficiently reduced to *Z*-alkenes in excellent yields under mild and environmentally acceptable conditions. No generation of *E*-alkenes was detected. The reaction again worked efficiently in the presence of different functional and protective groups, including those labile to common hydrogenation conditions, such as cyclopropane **7g**.^28^ It is especially remarkable that examples **8f**–**i**, employed in biomimetic cyclizations promoted by Cp_2_TiCl,^29^ were obtained in high yield and complete *Z* selectivity, providing an alternative route for the preparation of complex polyenes. These results confirmed that our reaction is an excellent alternative to classic semihydrogenation protocols and new TH procedures.

Moreover, we performed experiments to determine the possible mechanism involved in this process. First, we measured the amount of H_2_ gas generated in the presence of Mn and NH_4_Cl.^17,30^ The evolution of the generated H_2_ is depicted in Figure 1A. Under these conditions, a pressure of 0.4 atm of H_2_ was obtained (approximately 0.5 mmol).^31^ This result indicated a 1:2 molar relationship between H_2_ and Mn dust, which matched the stoichiometry of the optimized experimental conditions. The winding profile could be attributed to a passivation-cleaning process occurring on the manganese metal surface.

**Figure 1:**
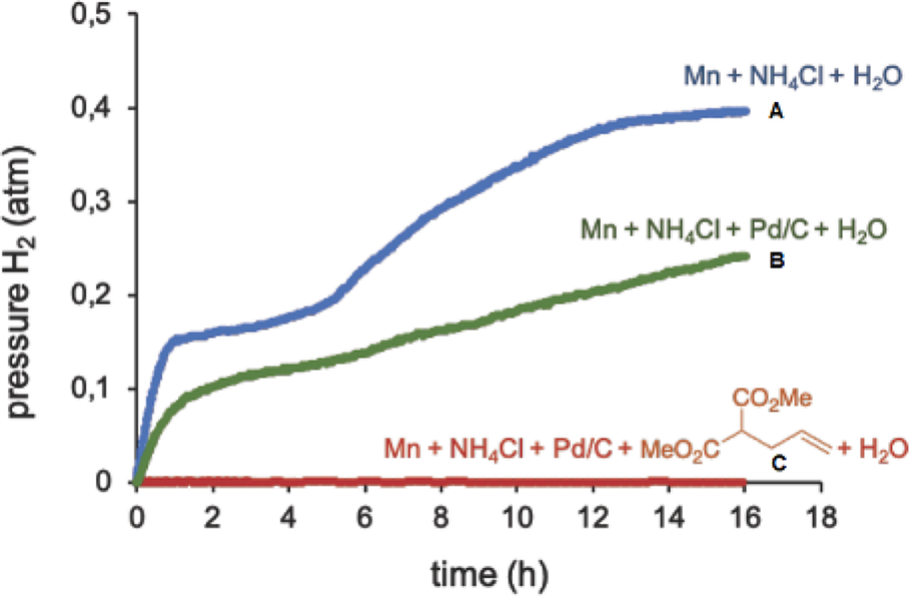
Determination of H_2_ generation under different conditions. H_2_ production was calculated by continuous monitoring of the pressure evolution using a pressure transducer (Man on the Moon X102 kit).^32^

Additionally, we determined the generated H_2_ in the presence of 5% Pd/C (2.4 mol % Pd, Figure 1B). After 16 h, a pressure of 0.22 atm was obtained, less than in Figure 1A. This could be due to the known adsorption of H_2_ on the surface of the catalyst.^1,33^ We also examined H_2_ evolution in the reduction of **1a**.^31^ As expected, no H_2_ pressure was detected (Figure 1C). ^1^H NMR spectroscopy of the crude product showed that the reaction was completed, giving **2a** in quantitative yields. This indicates that H_2_ gas generation is slow and controlled, being consumed immediately in the hydrogenation reaction. This matched the observed high selectivity.

We performed additional experiments to demonstrate that our protocol follows the “classic” hydrogenation mechanism (see Figures S1 and S2 in the Supporting Information). Thus, using the optimized conditions, we generated an amount of H_2_ gas after 16 h, and then **1a** was added. The reaction proceeded smoothly until complete consumption of H_2_ (after 7 h), yielding the expected **2a** in quantitative yield.^17^

With all of this information in hand, we propose the following tentative mechanism (Scheme 6).

**Scheme 6:**
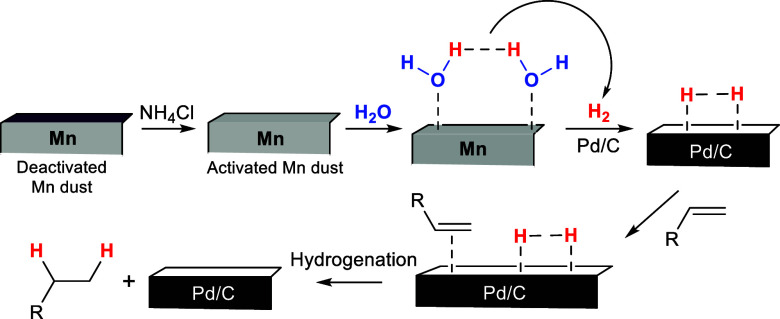
Proposed Mechanism for Mn/H_2_O-Promoted Hydrogenation

Our proposal begins with the activation of the surface of the deactivated Mn dust by using NH_4_Cl. The use of this salt for the activation and cleaning of metal surfaces is extensively known in the context of welding.^34^ Once the surface is “activated”, it can coordinate with water, as we previously proposed.^16,35^ In our case, Mn dust is essential for H_2_ generation (see Figure 1), and is not only a “reductant” of other transition metals involved in water dissociation, as has been described.^13^ The indicated coordination allows the weakening of the H–O bond of water,^36,37^ generating H atoms that could yield H_2_ gas.^35^ Then, in the presence of heterogeneous Pd/C, the reaction follows the classic mechanism^1^ proposed for hydrogenation: coordination of H_2_ and the substrate on the surface of the catalyst and subsequent addition of H atoms to the alkene, yielding the corresponding alkane and the released catalyst. This mechanism could be extended to the use of Lindlar’s catalyst for the semihydrogenation of alkynes.

In summary, we have developed a method for the reduction of multiple C–C bonds using Mn/H_2_O under simple, cheap, and environmentally acceptable conditions. The highly controlled generation of H_2_ allows for the selective reduction of alkenes with different substitution patterns. Additionally, the use of Mn, the third most abundant transition metal in Earth’s crust, which is cheap and accessible, and tap water, the cheapest and most accessible source of “H atoms”, guarantees the high availability of our methodology for its application in any laboratory around the world.

## Data Availability

The data underlying this study are available in the published article and its Supporting Information.

## Supporting Information Available

Additional experiments, detailed experimental procedures, characterization, and copies of ^1^H and ^13^C NMR spectra of the new and described compounds (PDF)
