# Supplementary material for: Mild and Selective Hydrogenation of Unsaturated Compounds Using Mn/Water as a Hydrogen Gas Source
Source: Org Lett. 2023 Dec 14;26(11):2147–51. doi: 10.1021/acs.orglett.3c03664 (PMC10964242; doi:10.1021/acs.orglett.3c03664)

Supporting Information

Mild, and Selective Hydrogenation of Unsaturated Compounds Using Mn/Water as Hydrogen Gas Source

Jennifer Rosales,[1] Tania Jiménez,[1] Rachid Chahboun,[1] Miguel A. Huertos,[2] Alba Millán,[1] and José Justicia[1,*]

[1]Department of Organic Chemistry, Faculty of Sciences, University of Granada, C. U. Fuentenueva s/n 18071 Granada (Spain). [2]University of Basque Country (UPV/EHU), Donostia-San Sebastian, 20018 San Sebastián (Spain); IKERBASQUE, Basque Fundation for Science, 48013 Bilbao, Spain.

E-mail: jjusti@ugr.es

Contents

- Synthetic processes and description of new compounds S2-S12
- References S12-S14
- ^{1}H and ^{13}C NMR spectra for described and new compounds S15-S45

General Details. THF was freshly distilled from Na. Other dry solvents, such as CH_2Cl_2 or acetone, were acquired from commercial suppliers. Lindar's catalyst was purchased from Sigma-Aldrich (62145-10G-F, Pd concentration 5%). Products were purified by flash chromatography on VWR silica gel 40-60 μm. Yields refer to analytically pure samples. NMR spectra were recorded in NMR Varian Direct Drive (400 MHz or 500 MHz) spectrometers. The following known compounds were isolated as pure samples and showed NMR spectra that matched those of the reported compounds: **1e**,[1] **1f**,[2] **1g**,[3] **1h**,[4] **1k**,[5] **1n**,[6] **1o**,[7] **1p**,[8] **1q**,[9] **1r**,[10] **2a**,[11] **2d**,[12] **2e**,[13] **2f**,[14] **2g**,[15] **2n**,[2] **2o**,[16] **2q**,[17] **2r**,[18] **2t**,[19] **3a**,[20] **4a**,[21] **4e**,[22] **5b**,[23] **5c**,[24] **7c**,[25] **7d**,[25] **7f-i**,[26] **8e**,[27] **8f-i**.[26]

Optimization of hydrogenation conditions

Table S1. Study of the hydrogenation reaction using different metals and additives

**Metal (E^0)** / **Addit. (pK_a)**	Ni (-0.25)	Fe (-0.44)	Zn (-0.76)	Mn (-1.18)	Al (-1.66)	Mg (-2.37)
Guanidine ·HCl (13.6)	0%	0%	7%	10%	0%	0%
Et_3N·HCl (10.8)	0%	0%	7%	10%	0%	0%
NH_4Cl (9.25)	0%	0%	10%	**99%**	0%	0%
Collidine ·HCl (7.43)	0%	23%	26%	99%	12%	9%
Lutidine ·HCl (6.65)	0%	35%	30%	99%	5%	10%
Pyridine ·HCl (5.23)	0%	65%	80%	99%	26%	16%

Table S2. Optimization of the reaction conditions for alkene hydrogenations

1a (mmol)	Mn (mmol)	NH_4Cl (mmol)	Catalyst (%mol Pd)	H_2O (mL)	Product (%)
1	8	3	5% Pd/C (0.5)	10	89[a]
1	8	2	5% Pd/C (0.5)	10	85
1	8	1	5% Pd/C (0.5)	10	32
1	4	3	5% Pd/C (0.5)	10	98
1	4	2	5% Pd/C (0.5)	10	99
1	4	1	5% Pd/C (0.5)	10	45
1	2	3	5% Pd/C (0.5)	10	95
**1**	**2**	**2**	**5% Pd/C (0.5)**	**10**	**99**
1	2	2	5% Pd/C (0.25)	10	97
1	2	2	5% Pd/Al_2O_3 (0.5)	10	96
1	2	2	$Pd(dba)_2$ (0.1 mmol)	5[b]	59
1	2	2	5% Rh/Al_2O_3 (0.5)	10	NR
1	2	2	Wilkinson (0.1 mmol)	10	NR
1	2	2	Wilkinson (0.1 mmol)	5[b]	NR
1	2	1	5% Pd/C (0.5)	10	20
1	1	3	5% Pd/C (0.5)	10	12
1	1	2	5% Pd/C (0.5)	10	NR
1	1	1	5% Pd/C (0.5)	10	NR
1	2	0	5% Pd/C (0.5)	10	NR
1	0	2	5% Pd/C (0.5)	10	NR
1	2	2	-	10	12

[a]See ref. 28. [b]THF:H_2O 1:1 (10 mL) was used.

Figure S1. Hydrogenation of **1a** after previous generation of H_2[a]

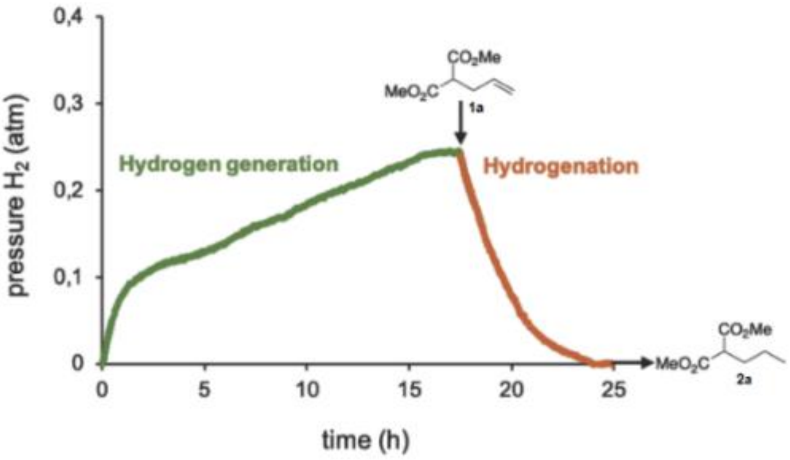

[a]The experiments were performed using the optimized conditions.

Figure S2. Determination of pressure of H_2 gas in presence of 5% Pd/C catalyst[a]

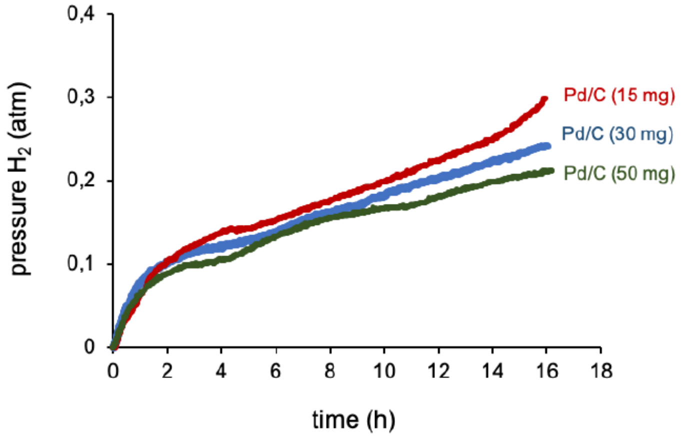

[a]The experiments were performed using the optimized conditions. The catalyst loading used was: Red (0.07 mmol of Pd); Blue (0.0141 mmol of Pd); Green (0.023 mmol of Pd).

Scheme S1. Studied tri- and tetrasubstituted alkenes

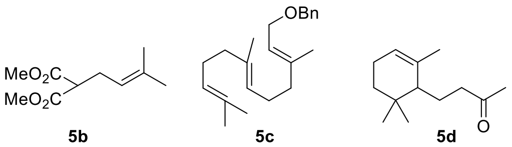

Synthesis of alkenes 1i and 1j

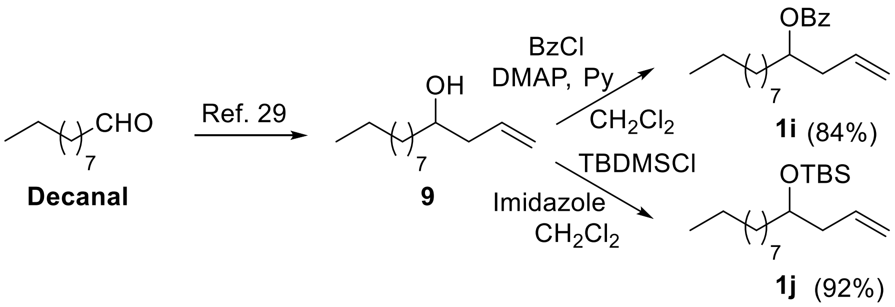

Preparation of alkene 1i: To a solution of alcohol **9** (200 mg, 1.01 mmol, see ref. 29) in CH_2Cl_2 (15 mL), BzCl (0.234 mL, 2.02 mmol), DMAP (370 mg, 3.03 mmol), and pyridine (0.25 mL, 3.1 mmol) were added, and the mixture was stirred at room temperature for 16 h. Then, the mixture was diluted with CH_2Cl_2 and washed with solution 2N HCl, and brine, dried over anhyd. Na_2SO_4, and the solvent removed. The residue was purified by flash chromatography on silica gel (hexane:AcOEt 97:3) to yield **1i** (257 mg, 84%). Colorless oil; ^{1}H NMR (400 MHz, $CDCl_3$): δ 8.04 (d, *J* = 5.9 Hz, 2H), 7.55 (t, *J* = 7.7 Hz, 1H), 7.44 (t, *J* = 7.7 Hz, 2H), 5.89–5.76 (m, 1H), 5.17 (quint, *J* = 7.3 Hz, 1H), 5.14–5.03 (m, 2H), 2.45 (t, *J* = 6.6 Hz, 2H), 1.76–1.61 (m, 2H), 1.43–1.16 (m, 14H), 0.87 (t, *J* = 6.6 Hz, 3H).

^{13}C NMR (125 MHz, $CDCl_3$): δ 166.4, 133.9, 132.9, 130.9, 129.7, 128.4, 117.9, 74.2, 38.8, 33.8, 32.0, 29.6, 29.4, 25.5, 22.8, 14.2. HRMS (ESI) m/z: $[M+Na]^+$ calcd for $C_{20}H_{30}O_2Na$, 325.2143; found: 325.2136.

Preparation of alkene 1j: To a solution of alcohol **9** (350 mg, 1.76 mmol, see ref. 29) in CH_2Cl_2 (18 mL), TBDMSCl (798 mg, 5.29 mmol), and imidazole (480 mg, 7.06 mmol) were added, and the mixture was stirred at room temperature for 16 h. Then, the mixture was diluted with CH_2Cl_2 and washed with saturated solution of NH_4Cl, dried over anhyd. Na_2SO_4, and the solvent removed. The residue was purified by flash chromatography on silica gel (hexane:AcOEt 97:3) to yield **1j** (510 mg, 92%). Colorless oil; ^1H NMR (400 MHz, $CDCl_3$): δ 5.87–5.76 (m, 1H), 5.06–4.98 (m, 2H), 3.68 (quint, *J* = 5.8 Hz, 1H), 2.26–2.13 (m, 2H), 1.42–1.40 (m, 2H), 1.31–1.19 (m, 14H), 0.90–0.87 (m, 12H), 0.04 (s, 6H). ^{13}C NMR (125 MHz, $CDCl_3$): δ 135.7, 116.6, 72.2, 42.1, 37.0, 32.1, 29.9, 29.8, 29.7, 29.5, 26.1, 25.5, 22.8, 18.3, 14.3, -4.2, -4.4. HRMS (ESI) m/z: $[M+Na]^+$ calcd for $C_{19}H_{40}OSiNa$, 335.2741; found: 335.2735.

Synthesis of alkenes 1u and 3f

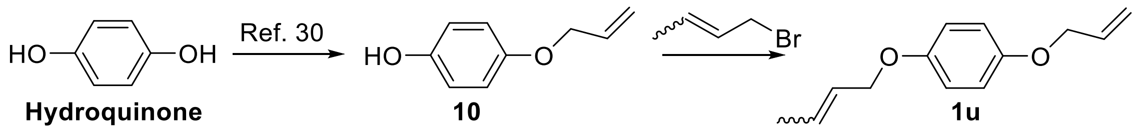

Preparation of 1u: To a mixture of **10** (400 mg, 2.66 mmol, see Ref. 30), and K_2CO_3 (736 mg, 5.33 mmol) in acetone (8 mL), *trans*-crotyl bromide (503 mg, 3.73 mmol, 15% of *cis*-isomer) was added, and the mixture was stirred at 70 ºC overnight. Then, the mixture was filtered, and the solvent removed. The residue was purified by flash chromatography on silica gel (hexane:AcOEt 9:1) to yield **1u** (434 mg, 80%). Yellowish oil; ^1H NMR (400 MHz, $CDCl_3$): δ 6.86 (s, 1H), 6.85 (s, 3H), 6.10–5.99 (m, 1H), 5.90–5.79 (m, 1H), 5.76–5.67 (m, 1H), 5.40 (dd, *J* = 16.8, 1.6 Hz, 1H), 5.27 (dd, *J* = 10.2, 1.6, Hz, 1H), 4.57–4.52 (m, 2H, minor isomer), 4.49 (dt, *J* = 5.4, 1.6 Hz, 2H), 4.41 (dt, *J* = 5.9, 1.2 Hz, 2H), 1.76 (d, *J* = 6.4 Hz, 3H), 1.73 (d, *J* = 6.4 Hz, 3H, minor isomer). ^{13}C NMR (125 MHz, $CDCl_3$): δ 153.1, 152.9, 133.7, 130.5, 128.5 (minor isomer), 126.5, 126.0 (minor isomer),

117.6, 115.8, 115.7, 69.6, 69.4, 64.4 (minor isomer), 18.0, 13.4 (minor isomer). HRMS (APCI) m/z: $[M+H]^+$ calcd for $C_{13}H_{17}O_2$, 205.1223; found: 205.1224.

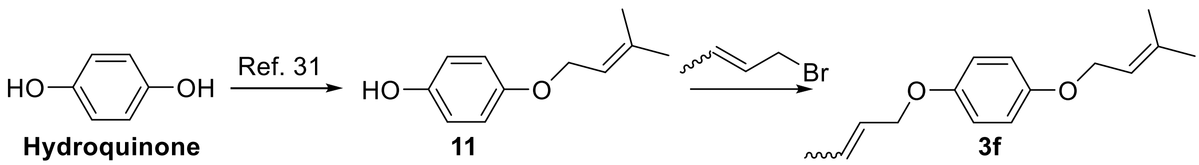

Preparation of 3f: To a mixture of **11** (400 mg, 2.24 mmol, see Ref. 31), and K_2CO_3 (620 mg, 4.49 mmol) in acetone (16 mL), trans-crotyl bromide (454 mg, 3.36 mmol, 15% of cis-isomer) was added, and the mixture was stirred at 50 ºC for 6 h. Then, the mixture was filtered, and the solvent removed. The residue was purified by flash chromatography on silica gel (hexane:AcOEt 97:3) to yield **3f** (465 mg, 89%). A 10% of isomer of γ-addition of crotyl bromide was observed. Colorless oil; ^{1}H NMR (400 MHz, $CDCl_3$): δ 6.86–6.83 (m, 4H), 6.11 (dd, *J* = 17.6, 10.9 Hz, 1H, γ-isomer), 5.89–5.80 (m, 1H), 5.77–5.67 (m, 1H), 5.49 (t, *J* = 6.8 Hz, 1H), 5.15–5.08 (m, 2H, γ-isomer), 4.55 (d, *J* = 6.7 Hz, 2H, minor isomer), 4.46 (d, *J* = 6.7 Hz, 2H), 4.40 (dt, *J* = 5.8, 2.2 Hz, 2H), 1.80 (s, 3H), 1.76 (dd, *J* = 6.4, 1.3 Hz, 3H), 1.74 (s, 3H). ^{13}C NMR (125 MHz, $CDCl_3$): δ 153.2, 153.0, 138.0, 130.4, 128.5 (minor isomer), 126.5, 126.1 (minor isomer), 120.1, 115.7, 115.67 (minor isomer), 115.6, 114.7 (minor isomer), 69.5, 65.4, 64.4 (minor isomer), 26.8 (minor isomer), 26.0, 18.3, 18.0, 13.4 (minor isomer). HRMS (APCI) m/z: $[M+H]^+$ calcd for $C_{15}H_{21}O_2$, 233.1536; found: 233.1534.

General procedure for hydrogenation of alkenes 1a-v, 3a-f and 5a

A sample of alkene (1 mmol, 1 eq) was added to a mixture of NH_4Cl (2 mmol, 2 eq), Mn dust (2 mmol, 2 eq), and 5% Pd/C (see Table S3) in water (0.1 M). The flask was strongly sealed, and the mixture was stirred for 8 h (monosubstituted alkenes) or 24 h (disubstituted alkenes) at rt. Then, water was added, and the mixture was extracted with AcOEt, washed with brine, and thoroughly filter before being dried over anhyd. Na_2SO_4, and the solvent removed. Unless otherwise stated, the compounds were weighed, isolated yields calculated, and characterized without further purification. (See results in Schemes 3-5, main text).

Table S3. Pd/C catalyst loading in each reaction.

Starting Material	Amount (mg)	mmol	5% Pd/C (mg)	5% Pd/C (mol% Pd)
**1a**	100	0.58	10	*0.8*
**1b**	100	0.40	10	*1.17*
**1c**	100	0.74	10	*0.63*
**1d**	100	0.47	10	*1*
**1d (deut)**	100	0.47	10	*1*
**1e**	100	0.40	10	*1.17*
**1f**	100	0.37	10	*1.27*
**1g**	100	0.70	10	*0.67*
**1h**	100	0.42	10	*1.12*
**1i**	100	0.33	10	*1.4*
**1j**	100	0.32	10	*1.47*
**1k**	100	0.41	10	*1.15*
**1l**	100	0.65	10	*0.72*
**1m**	100	0.62	10	*0.76*
**1n**	100	0.59	10	*0.8*
**1o**	100	0.63	10	*0.75*
**1p**	100	0.35	10	*1.34*
**1q**	100	0.59	10	*0.8*
**1r**	100	0.61	10	*0.77*
**1s**	100	0.65	10	*0.72*
**1t**	100	0.32	10	*1.47*
**1u**	100	0.49	10	*0.96*
**1v**	100	0.46	10	*1.02*
**3a**	100	0.54	10	*0.87*
**3b**	100	0.60	10	*0.78*
**3c**	100	0.74	10	*0.63*
**3c (deut)**	100	0.74	10	*0.63*
**3d**	100	0.57	10	*0.82*
**3e**	100	0.55	10	*0.85*
**3f**	100	0.43	10	*1.09*
**5a**	100	0.52	10	*0.9*

General procedure for hydrogenation of alkynes 7a-h

A sample of alkyne (1 mmol, 1 eq) was added to a mixture of NH_4Cl (2 mmol, 2 eq), Mn dust (2 mmol, 2 eq), and Lindlar's catalyst (5% in Pd, see Table S4) in a 1:4 mixture of THF:water (0.1 M). The flask was strongly sealed, and the mixture was stirred for 48 h at rt. Water was added, and the mixture was extracted with AcOEt, washed with brine, and thoroughly filter before being dried over anhyd. Na_2SO_4, and the solvent removed. Unless otherwise stated, the compounds were weighed, isolated yields calculated, and characterized without further purification. (See results in Scheme 6, main text).

Table S4. Lindlar catalyst loading in each reaction.

Starting Material	Amount (mg)	mmol	5% Lindlar (mg)	5% Lindlar (mol% Pd)
**1a**	100	0.59	10	*0.8*
**7b**	100	0.76	10	*0.65*
**7c**	100	0.62	10	*0.76*
**7d**	100	0.57	10	*0.79*
**7e**	100	0.56	10	*0.78*
**7f**	100	0.44	10	*1.08*
**7g**	100	0.40	10	*1.14*
**7h**	100	0.35	10	*1.34*
**7i**	100	0.31	10	*1.48*

Characterization of hydrogenated compounds.

Compound *d₄*-2d. (90% deuterium incorporation). 93 mg, 91%. Colorless oil.

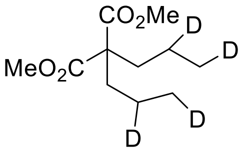

***d*$_4$-2d**

^{1}H NMR (400 MHz, CDCl$_3$): δ 3.70 (s, 6H, minor isomer), 3.69 (s, 6H), 1.87–1.78 (m, 4H), 1.20–1.07 (s, 2H), 0.92–0.82 (m, 4H). ^{13}C NMR (125 MHz, CDCl$_3$): δ 172.6, 57.8, 52.6 (minor isomer), 52.3, 34.9, 17.8–17.1 (m), 14.6–13.5 (m). HRMS (APCI) m/z: [M+H]$^+$ calcd for $C_{11}H_{17}D_4O_4$, 221.1685; found: 221.1679.

Compound 2h. 98 mg, 97%. Colorless oil.

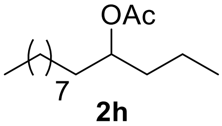

2h

^{1}H NMR (400 MHz, CDCl$_3$): δ 4.87 (quint, J = 7.0, 1H), 2.03 (s, 3H), 1.55–1.43 (m, 4H), 1.37–1.19 (s, 16H), 0.94–0.80 (m, 6H). ^{13}C NMR (125 MHz, CDCl$_3$): δ 171.1, 74.3, 36.4, 34.3, 32.0, 29.7, 29.4, 25.5, 22.8, 21.4, 18.7, 14.2, 14.1. HRMS (ESI) m/z: [M+Na]$^+$ calcd for $C_{15}H_{30}O_2Na$, 265.2138; found: 265.2134.

Compound 2i. 88 mg, 87%. Yellowish oil.

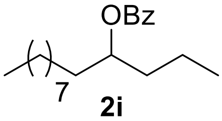

2i

^{1}H NMR (400 MHz, CDCl$_3$): δ 8.05 (d, J = 8.6 Hz, 2H), 7.55 (t, J = 7.5 Hz, 1H), 7.44 (t, J = 7.5 Hz, 2H), 5.20–5.10 (m, 1H), 1.75–1.55 (m, 4H), 1.46–1.20 (m, 16H), 0.93 (t, J = 7.3 Hz, 3H), 0.87 (t, J = 7.3 Hz, 3H). ^{13}C NMR (125 MHz, CDCl$_3$): δ 166.5, 132.8, 131.0, 129.7, 128.4, 75.0, 36.6, 34.4, 32.0, 29.7,

29.6, 29.4, 25.5, 22.8, 18.8, 14.3, 14.2. HRMS (ESI) m/z: $[M+Na]^+$ calcd for $C_{20}H_{32}O_2Na$, 327.2290; found: 327.2300.

Compound 2j. 88 mg, 87%. Colorless oil.

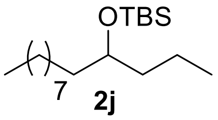

2j

^{1}H NMR (400 MHz, $CDCl_3$): δ 3.63 (quint, *J* = 5.5 Hz, 1H), 1.46–1.37 (m, 4H), 1.35–1.25 (s, 16H), 0.92–0.87 (m, 15H), 0.4 (s, 6H). ^{13}C NMR (125 MHz, $CDCl_3$): δ 72.4, 39.6, 37.4, 32.1, 30.0, 29.9, 29.8, 29.7, 29.5, 26.1, 25.5, 22.9, 18.8, 18.3, 14.5, 14.3, -4.2, -4.3. HRMS (ESI) m/z: $[M+Na]^+$ calcd for $C_{19}H_{42}OSiNa$, 337.2897; found: 337.2898.

Compound 2k. 61 mg, 81%. Colorless oil.

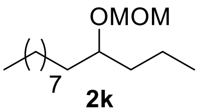

2k

^{1}H NMR (400 MHz, $CDCl_3$): δ 4.64 (s, 2H), 3.52 (quint, *J* = 5.6 Hz, 1H), 3.37 (s, 3H), 1.53–1.39 (m, 4H), 1.38–1.19 (s, 16H), 0.91 (t, *J* = 7.2 Hz, 3H), 0.87 (t, *J* = 7.2 Hz, 3H). ^{13}C NMR (125 MHz, $CDCl_3$): δ 95.5, 77.4, 55.6, 36.7, 34.5, 32.0, 30.0, 29.8, 29.7, 29.5, 25.4, 22.8, 18.7, 14.4, 14.2. HRMS (ESI) m/z: $[M+Na]^+$ calcd for $C_{15}H_{32}O_2Na$, 267.2295; found: 267.2290.

Compound 2p. 87 mg, 86%. Yellowish oil.

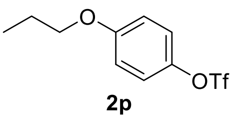

2p

^{1}H NMR (400 MHz, $CDCl_3$): δ 7.18 (d, *J* = 9.2 Hz, 2H), 6.91 (d, *J* = 9.2 Hz, 2H), 3.92 (t, *J* = 6.5 Hz, 2H), 1.82 (sext, *J* = 6.6 Hz, 2H), 1.04 (t, *J* = 7.4 Hz, 3H). ^{13}C NMR (125 MHz, $CDCl_3$): δ 158.8, 143.0, 122.4, 118.9 (q, *J* = 320.8 Hz, *C*-F_3), 115.7, 70.2, 22.6, 10.6. HRMS (APCI) m/z: $[M]^+$ calcd for $C_{10}H_{11}F_3O_4S$, 284.0330; found: 284.0320.

Compound 2s. 94 mg, 93%. Colorless oil.

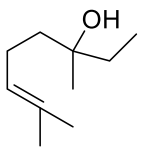

2s

^{1}H NMR (400 MHz, $CDCl_3$): δ 5.13 (t, *J* = 7.0 Hz, 1H), 2.03 (q, *J* = 7.8 Hz, 2H), 1.68 (s, 3H), 1.62 (s, 3H), 1.53–1.41 (m, 4H), 1.15 (s, 3H), 0.89 (t, *J* = 7.0 Hz, 3H). ^{13}C NMR (125 MHz, $CDCl_3$): δ 131.8, 124.7, 73.1, 41.2, 34.4, 26.4, 25.8, 22.8, 17.8, 8.4. HRMS (APCI) m/z: $[M-H]^+$ calcd for $C_{10}H_{19}O$, 155.1430; found: 155.1430.

Compound 2u. 71 mg, 94%. Yellowish oil.

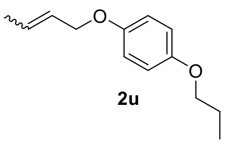
2u

^{1}H NMR (400 MHz, $CDCl_3$): δ 6.85–6.80 (m, 4H), 5.90–5.80 (m, 1H), 5.77–5.68 (m, 1H), 4.40 (dt, *J* = 6.0, 1.2 Hz, 2H), 3.87 (t, *J* = 6.6, 2H), 1.83–1.69 (m, 5H), 1.03 (t, *J* = 7.4 Hz, 3H). ^{13}C NMR (125 MHz, $CDCl_3$): δ 153.5, 152.9, 130.5, 126.5, 115.8, 115.5, 70.3, 69.5, 22.8, 18.0, 10.7. HRMS (APCI) m/z: $[M+H]^+$ calcd for $C_{13}H_{19}O_2$, 207.1380; found: 207.1381.

Compound 2v. 69 mg, 91%. Brown oil.

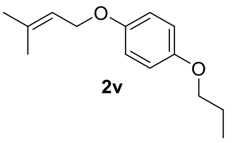
2v

^{1}H NMR (400 MHz, $CDCl_3$): δ 6.88–6.81 (m, 4H), 5.50 (t, *J* = 6.7 Hz, 1H), 4.46 (d, *J* = 6.9 Hz, 2H), 4.03 (t, *J* = 6.8 Hz, 2H, minor isomer), 3.87 (t, *J* = 6.8 Hz, 2H), 1.82–1.79 (m, 2H), 1.80 (s, 3H), 1.78 (s, 3H, minor isomer), 1.76 (s, 3H, minor isomer), 1.74 (s, 3H), 1.03 (t, *J* = 7.3 Hz, 3H). ^{13}C NMR (125 MHz, $CDCl_3$): δ 153.4, 153.0, 138.0, 120.1, 115.7, 115.5, 70.3, 65.5, 31.0 (minor isomer), 26.0, 22.8, 18.3, 10.7. HRMS (APCI) m/z: $[M+H]^+$ calcd for $C_{14}H_{21}O_2$, 221.1536; found: 221.1539.

Compound *d₂*-4c. (81% deuterium incorporation). 86 mg, 85%. Colorless oil.

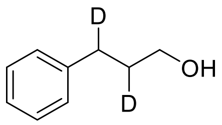
***d*$_2$-4c**

^{1}H NMR (400 MHz, $CDCl_3$): δ 7.30 (dd, *J* = 8.2, 6.9 Hz, 2H), 7.22 (d, *J* = 7.4 Hz, 3H), 3.68 (d, *J* = 6.2 Hz, 2H), 2.77–2.69 (m, 1H), 1.96–1.86 (m, 1H), 1.69 (bs, 1H, OH). ^{13}C NMR (125 MHz, $CDCl_3$): δ 141.9, 128.52, 128.50, 126.0, 62.3, 34.3–33.6 (m), 32.2–31.5 (m). HRMS (APCI) m/z: $[M+H]^+$ calcd for $C_9H_{11}D_2O$, 139.1086; found: 139.1087.

Compound 4f. (10% of isomer from γ-addition of crotyl bromide was observed). 67 mg, 88%. Yellowish oil.

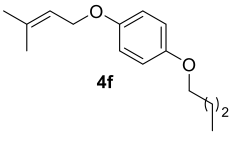
4f

^{1}H NMR (400 MHz, $CDCl_3$): δ 6.88-6.78 (m, 4H), 5.49 (t, *J* = 6.7 Hz, 1H), 4.82 (d, *J* = 6.7 Hz, 2H, minor isomer), 4.46 (d, *J* = 6.8 Hz, 2H), 4.03 (t, *J* = 6.9 Hz, 2H, minor isomer), 3.91 (t, *J* = 6.9, 2H), 2.48 (t, *J* = 6.9, 2H, minor isomer), 1.82–1.79 (m,

2H), 1.80 (s, 3H), 1.76 (s, 3H, minor isomer), 1.75 (s, 3H, minor isomer), 1.73 (s, 3H), 1.53–1.43 (m, 2H), 1.01 (t, J = 7.4 Hz, 3H, minor isomer), 0.97 (t, J = 7.4 Hz, 3H). ^{13}C NMR (125 MHz, $CDCl_3$): δ 153.4, 153.0, 142.5 (minor isomer), 138.0, 125.3 (minor isomer), 120.1, 115.7, 115.5, 114.5 (minor isomer), 112.0 (minor isomer), 68.5, 67.3 (minor isomer), 65.5, 37.5 (minor isomer), 34.4 (minor isomer), 31.6, 30.4 (minor isomer), 29.8 (minor isomer), 26.0, 22.9 (minor isomer), 19.4, 18.3, 14.0. HRMS (APCI) m/z: $[M+H]^+$ calcd for $C_{15}H_{23}O_2$, 235.1693; found: 235.1691.

Compound 6a. (83:17 Mixture **6a**:**6a-*red***).[32] 69 mg, 68%. Yellowish oil.

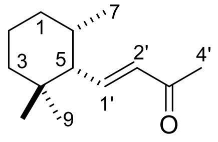

6a

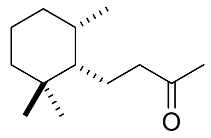

6a-*red*

^{1}H NMR (400 MHz, $CDCl_3$): δ 6.84 (dd, J = 15.7, 11.0 Hz, 1H), 6.03 (d, J = 15.7 Hz, 1H), 2.39 (t, J = 8.2 Hz, 2H, **6a-*red***), 2.25 (s, 3H), 2.12 (s, 3H, **6a-*red***), 2.02–1.92 (m, 1H), 1.89 (dd, J = 11.0, 4.0 Hz, 1H), 1.64-1.50 (m, 2H), 1.49-1.33 (m, 1H), 1.32-1.20 (m, 1H), 1.10 (m, 2H), 1.02 (s, 3H), 0.96 (s, 3H, **6a-*red***), 0.85 (s, 3H, **6a-*red***), 0.84 (d, J = 6.9 Hz, 3H, **6a-*red***), 0.74 (s, 3H), 0.72 (d, J = 6.9 Hz, 3H). ^{13}C NMR (125 MHz, $CDCl_3$): δ 209.6 (**6a-*red***), 198.3, 147.9, 133.6, 55.1, 49.2 (**6a-*red***), 39.9 (**6a-*red***), 34.5, 34.4 (**6a-*red***), 33.8, 31.3, 30.7, 30.4 (**6a-*red***), 30.0 (**6a-*red***), 29.7, 28.5 (**6a-*red***), 27.3, 27.1, 22.4 (**6a-*red***), 22.3, 21.1, 19.6 (**6a-*red***). 1D-NOESY experiences: proton irradiated (nOe observed): H_3-7 (H_2-1, H5, H-6), H_3-8 (H2, H5, H6, H_3-9), H_3-9 (H_2-1, H5, H8). HRMS (APCI) m/z: $[M+H]^+$ calcd for $C_{13}H_{23}O$, 195.1743; found: 195.1749.

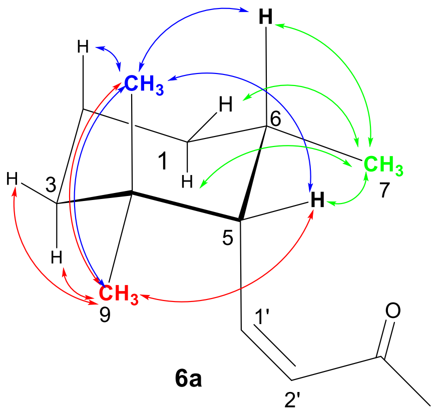

6a

Compound 8d. 97 mg, 96%.Yellowish oil.

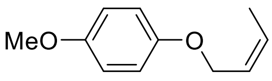

8d

^{1}H NMR (400 MHz, $CDCl_3$): δ 6.91–6.84 (m, 4H), 5.82–5.68 (m, 2H), 4.56 (d, J = 5.3 Hz, 2H), 3.77 (s, 3H), 1.74 (d, J = 5.3 Hz, 3H). ^{13}C NMR (125 MHz, $CDCl_3$): δ 153.9, 153.0, 128.5, 126.0, 115.8, 114.7, 64.4, 55.8, 13.4. HRMS (APCI) m/z: $[M+H]^+$ calcd for $C_{11}H_{15}O_2$, 179.0994; found: 179.0997.

References

1.- Yoshimitsu, T.; Makino, T.; Nagaoka, H. Total synthesis of (+)-muconin. *J. Org. Chem.* **2004**, *69*, 1993–1998.

2.- Balas, L.; Bertrand-Michel, J.; Viars, F.; Faugere, J.; Lefort, C.; Caspar-Bauguil, S.; Langin, D.; Durand, T. Regiocontrolled syntheses of FAHFAs and LC-MS differentiation of regioisomers. *Org. Biomol. Chem.* **2016**, *14*, 9012–9020.

3.- Li, X.; Sun, B.; Zhou, J.; Jin, C.; Yu, C. Regioselective acetoxylation of terminal olefins using a palladium(II)–thiadiazole catalyst. *Eur. J. Org. Chem.* **2019**, 2635–2638.

4.- Gogoi, N.; Boruwa, J.; Barua, N. C. A concise total synthesis of antifungal antibiotic (+)-preussin. *Eur. J. Org. Chem.* **2006**, 1722–1725.

5.- Ning, X. -S.; Wang, M. -M.; Yao, C. -Z.; Chen, X. -M.; Kang, Y. -B. *tert*-Butyl nitrite: Organic redox cocatalyst for aerobic aldehyde-selective Wacker–Tsuji oxidation. *Org. Lett.* **2016**, *18*, 2700–2703.

6.- Angamuthu, V.; Rahman, F. -Ur.; Petroselli, M.; Li, Y.; Yu, Y.; Rebek Jr, J. Mono epoxidation of α,ω-dienes using NBS in a water-soluble cavitand. *Org. Chem. Front.* **2019**, *6*, 3220–3223.

7.- Yu, L.; Li, H.; Zhang, X.; Ye, J.; Liu, J.; Xu, Q.; Lautens, M. Organoselenium-catalyzed mild dehydration of aldoximes: An unexpected practical method for organonitrile synthesis. *Org. Lett.* **2014**, *16*, 1346–1349.

8.- Tsukamoto, H.; Suzuki, R.; Kondo, Y. Revisiting benzenesulfonyl linker for the deoxygenation and multifunctionalization of phenols. *J. Comb. Chem.* **2006**, *8,* 289–292.

9.- Schlüter, J.; Blazejak, M.; Hintermann, L. Aluminum-catalyzed hydroalkoxylation at elevated temperatures: Fast and simple access to coumarans and other oxygen heterocycles. *ChemCatChem.* **2013**, *5*, 3309–3315.

10.- Brucelle, F.; Renaud, P. Synthesis of indolines, indoles, and benzopyrrolizidinones from simple aryl azides. *Org. Lett.* **2012**, *14*, 3048-3051.

11.- Campaña, A. G.; Estévez, R. E.; Fuentes, N.; Robles, R.; Cuerva, J. M.; Buñuel, E.; Cárdenas, D.; Oltra, J. E. Unprecedented hydrogen transfer from water to alkenes and alkynes mediated by Ti^{III} and late transition metals. *Org. Lett.* **2007**, *9*, 2195–2198.

12.- Böse, D.; Denmark, S. E. Investigating the enantiodetermining step of a chiral Lewis-base catalyzed bromocycloetherification of privileged alkenes. *Synlett* **2018**, *29*, 433–439.

13.- Zhou, F.; Zhu, J.; Zhang, Y.; Zhu, S. NiH-catalyzed reductive relay hydroalkylation: A strategy for the remote C(sp^3)-H alkylation of alkenes. *Angew. Chem. Int. Ed.* **2018**, *57*, 4058–4062.

14.- Ito, H.; Watanabe, A.; Sawamura, M. Versatile dehydrogenative alcohol silylation catalyzed by Cu(I)-phosphine complex. *Org. Lett.* **2005**, *7*, 1869–1871.
15.- Höfener, S.; Lauterwasser, F.; Bräse, S. Second-generation paracyclophane imine ligands for the dialkylzinc addition to aldehydes. Optimization of the branched side chain leads to improvement for aliphatic aldehydes. *Adv. Synth. Catal.* **2004**, *346*, 755–759.
16.- Xu, S.; Cai, T.; Yun, Z. Cobalt-containing mesoporous ZSM-5 zeolite catalyzed C=C bond cleavage of alkenes to form nitriles. *Synlett* **2016**, *27*, 221–224.
17.- Guo, Y.; Fan, X. -M.; Nie, M.; Liu, H. -W.; Liao, D. -H.; Pan, X. -D.; Ji, Y. -F. Practical ligand-free copper-catalysed short-chain alkoxylation of unactivated aryl bromides. *Eur. J. Org. Chem.* **2015**, 4744–4755.
18.- Nacario, R.; Kotakonda, S.; Fouchard, D. M. D.; Viranga Tillekeratne, L. M.; Hudson, R. A. Reductive monoalkylation of aromatic and aliphatic nitro compounds and the corresponding amines with nitriles. *Org. Lett.* **2005**, *7*, 471–474.
19.- Decorzant, R.; Vial, C.; Näf, F. A short synthesis of ambrox® from sclareol. *Tetrahedron* **1987**, *43*, 1871-1879.
20.- Glorius, F.; Pfaltz, A. *Org. Lett.* **1999**, *1*, 141–144.
21.-O'Mahony, R. M.; Lynch, D.; Hayes, H. L. D.; Thuama, E. N.; Donnellan, P.; Jones, R. C.; Glennon, B.; Collins, S. G.; Maguire, A. R. Exploiting the continuous *in situ* generation of mesyl azide for use in a telescoped process. *Eur. J. Org. Chem.* **2017**, 6533–6539.
22.- Gieshoff, T. N.; Chakraborty, U.; Villa, M.; von Wangelin, A. J. Alkene hydrogenations by soluble iron nanocluster catalysts. *Angew. Chem. Int. Ed.* **2017**, *56*, 3585–3589.
23.- Nyfeler, E.; Renaud, P. Decarboxylative radical azidation using MPDOC and MMDOC esters. *Org. Lett.* **2008**, *10*, 985–988.

24.- Yanagisawa, A.; Hibino, H.; Habaue, S.; Hisada, Y.; Yasue, K.; Yamamoto, H. Regio- and stereoselective synthesis of 1,5-dienes using allylic barium reagents. *Bull. Chem. Soc. Jpn.* **1995**, *68*, 1263–1268.

25.- Efe, C.; Lykakis, I. N.; Stratakis, M. Gold nanoparticles supported on TiO_2 catalyse the cycloisomerisation/oxidative dimerisation of aryl propargyl ethers. *Chem. Commun.* **2011**, *47*, 803–805.
26.- Justicia, J.; Jiménez, T.; Miguel, D.; Contreras-Montoya, R.; Chahboun, R.; Álvarez-Manzaneda, E.; Collado-Sanz, D.; Cárdenas, D. J.; Cuerva, J. M. Titanocene(III)-catalyzed 6-*exo versus* 7-*endo* cyclizations of epoxypolyprenes: Efficient control and synthesis of versatile terpenic building blocks. *Chem. Eur. J.* **2013**, *19*, 12825–12833.
27.- Vasilikogiannaki, E.; Titilas, I.; Vassilikogiannakis, G.; Stratakis, M. *cis*-Semihydrogenation of alkynes with amine borane complexes catalyzed by gold nanoparticles under mild conditions. *Chem. Commun.* **2015**, *51*, 2384–2387.
28.- Jiménez, T.; Barea, E.; Oltra, J. E.; Cuerva, J. M.; Justicia, J. Mn(0)-mediated chemoselective reduction of aldehydes. Application to the synthesis of α-deuterioalcohols. *J. Org. Chem.* **2010**, *75*, 7022–7025.
29.- Dommisse, A.; Wirtz, J.; Kock, K.; Barthlott, W.; Kolter, T. Synthesis of (*S*)-nonacosan-10-ol, the major component of tubular plant wax crystals. *Eur. J. Org. Chem.* **2007**, 3508–3511.

30.- Gautier, A.; Mulatier, J. -C.; Crassous, J.; Dutasta, J. -P. Chiral trialkanolaminolamine-based hemicryptophanes: Synthesis and oxovanadium complex. *Org. Lett.* **2005**, *7*, 1207–1210.
31.- Schmidt, B.; Riemer, M. Microwave-promoted deprenylation: prenyl ether as a thermo-labile phenol protecting group. *Synthesis* **2016**, *48*, 1399-1406.
32.- When the hydrogenation reaction was left for 76 h, a 89% of fully reduced compound **6a-*red*** was obtained as the sole product. Its spectroscopic data matched previously described: Kantam, M. L.; Kishore, R.; Yadav, J.; Sudhakar, M.; Venugopal, A. Chemoselective hydrogenation of the olefinic bonds using a palladium/magnesium-lanthanum mixed oxide catalyst. *Adv. Synth. Catal.* **2012***, 354,* 663–669.

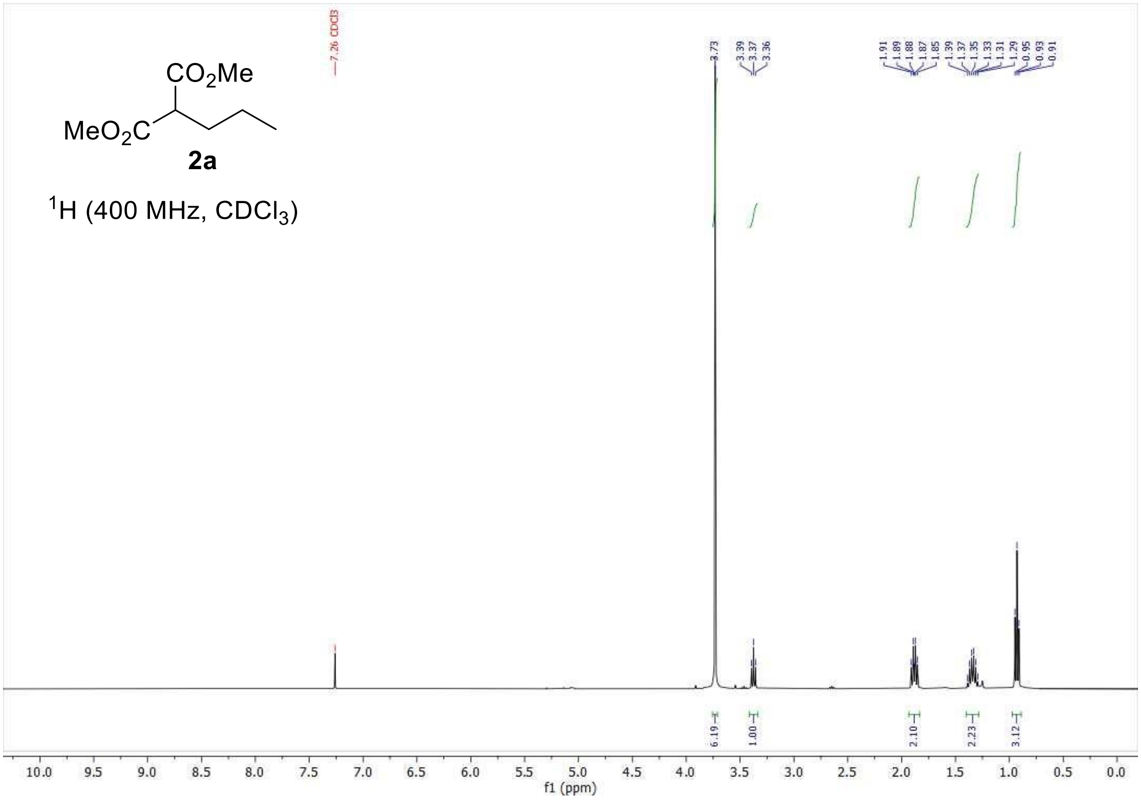

2a

^1H (400 MHz, $CDCl_3$)

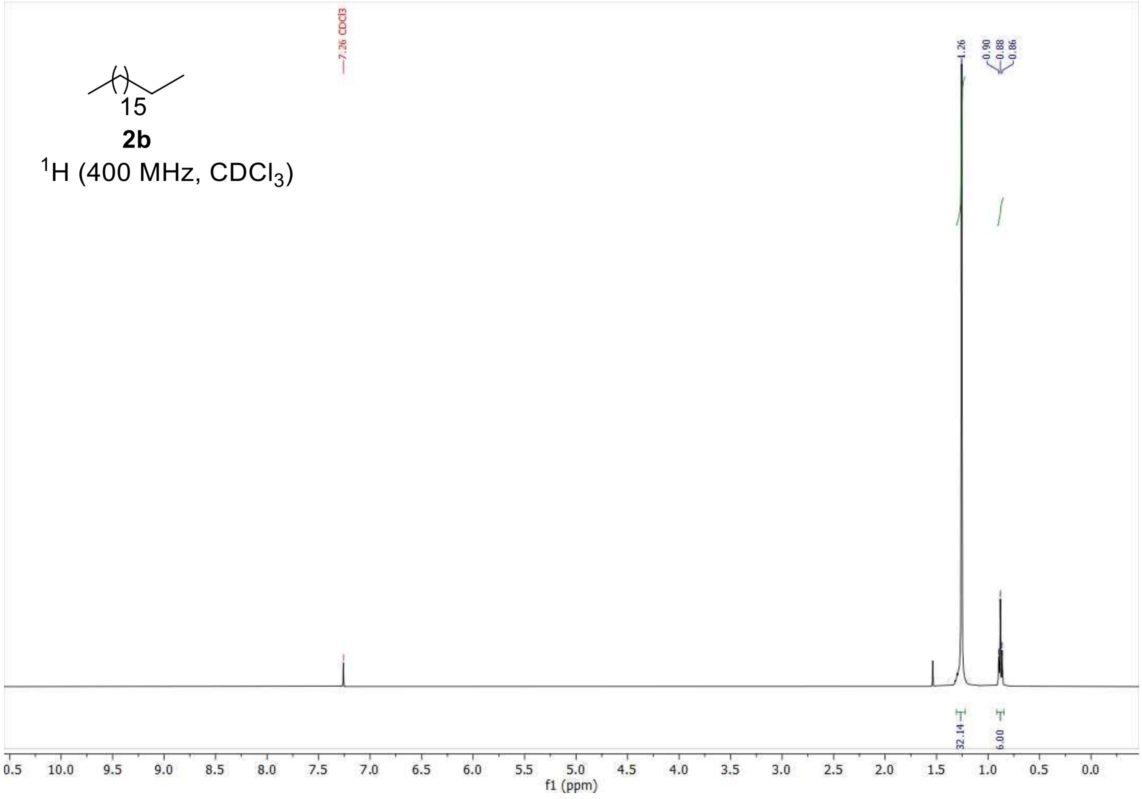

2b

^1H (400 MHz, $CDCl_3$)

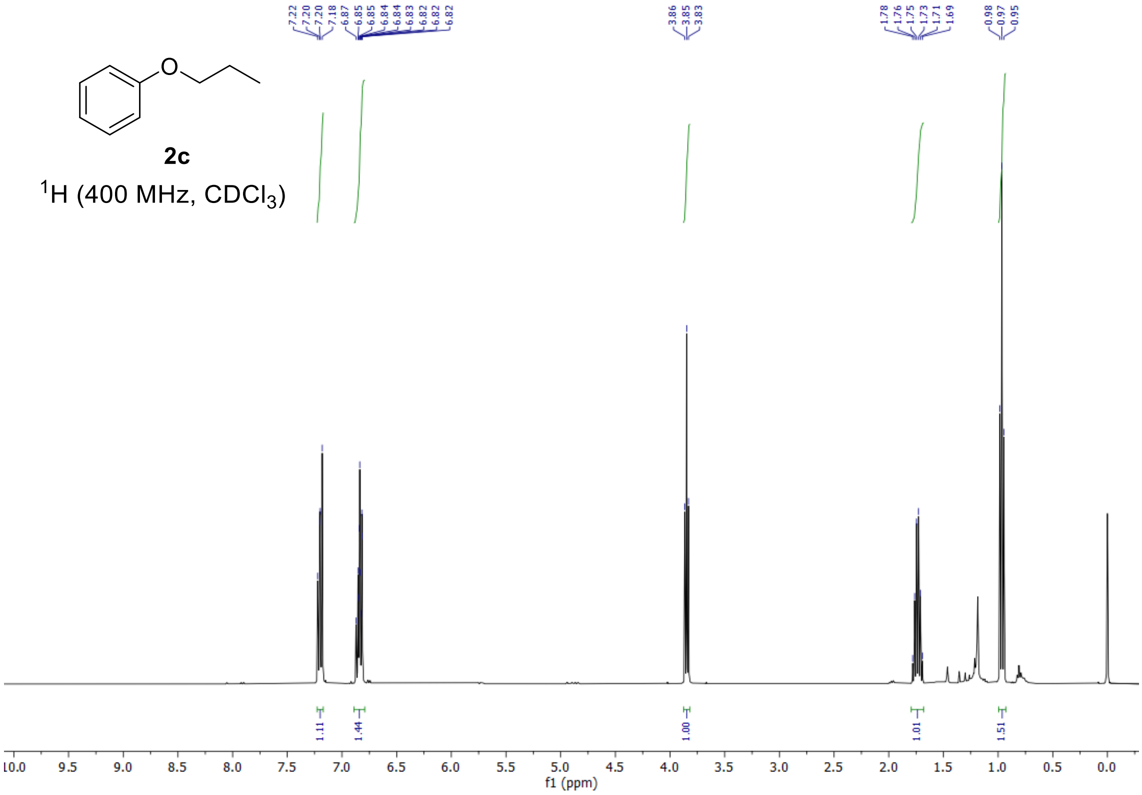

2c
1H (400 MHz, CDCl3)
f1 (ppm)

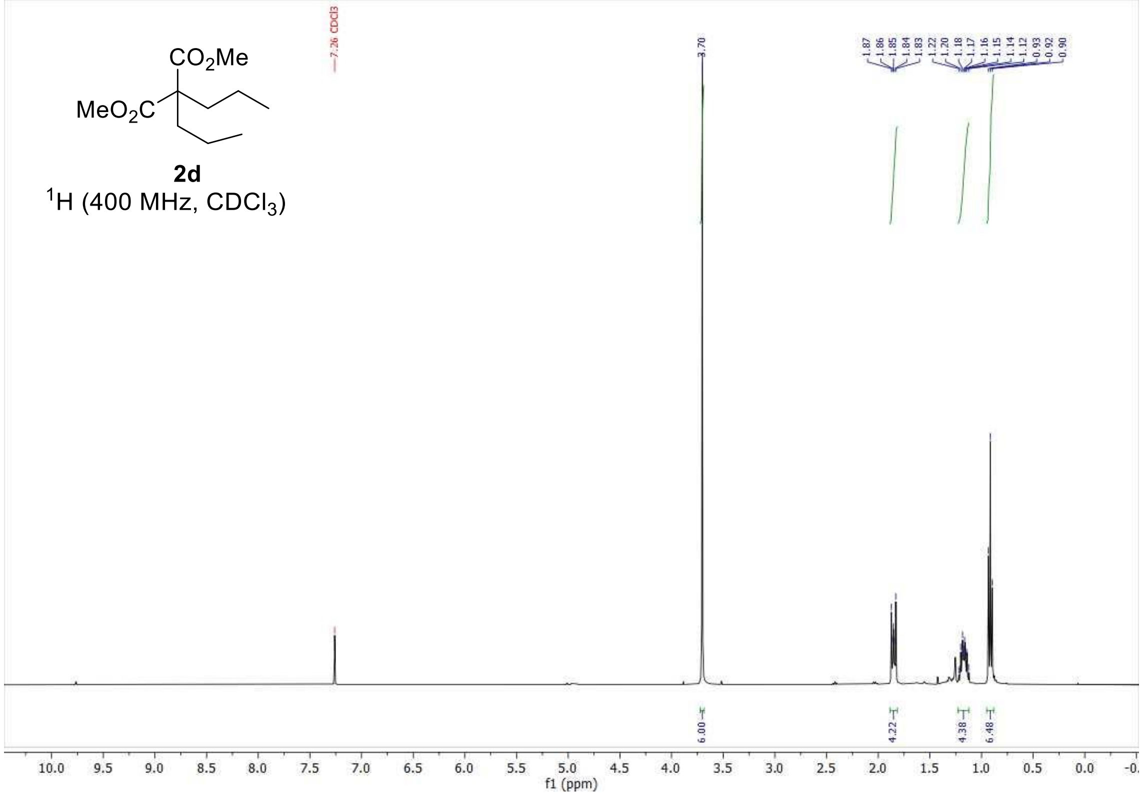

CO2Me
MeO2C
2d
1H (400 MHz, CDCl3)
f1 (ppm)

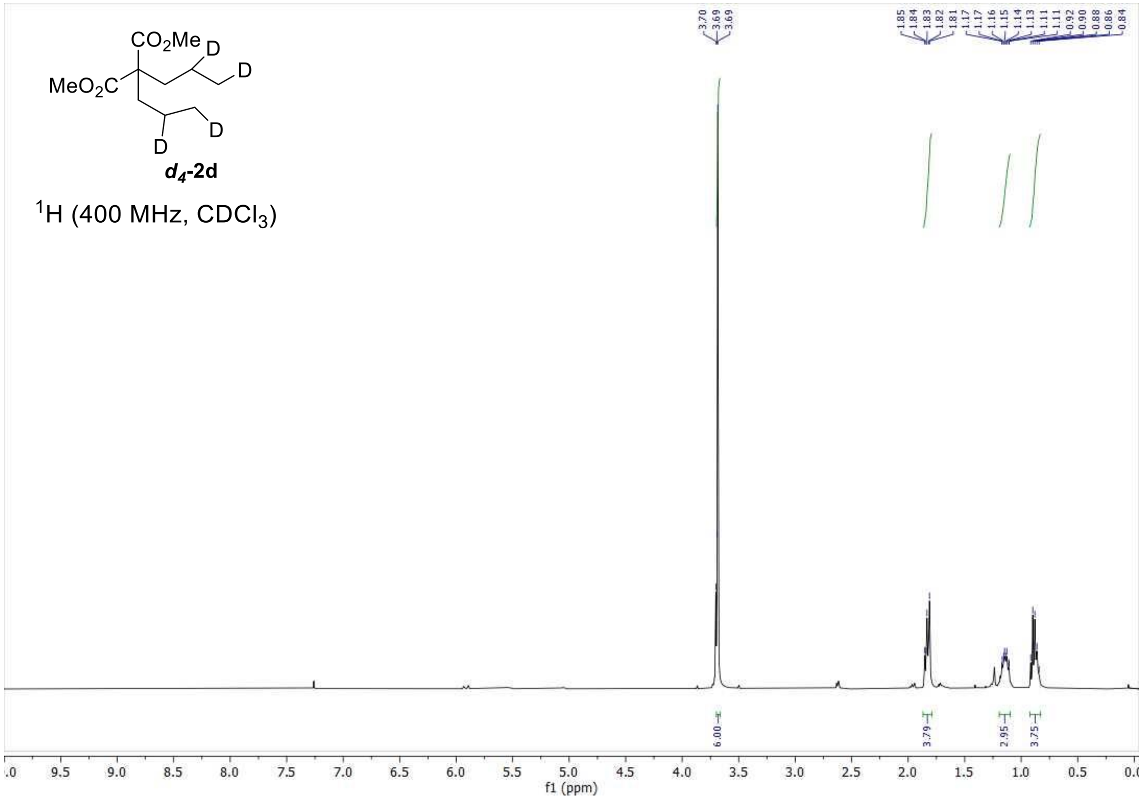

CO2Me
MeO2C
D
D
D
D
d4-2d
1H (400 MHz, CDCl3)
3.70
3.69
3.69
1.85
1.84
1.83
1.82
1.81
1.17
1.17
1.16
1.15
1.14
1.13
1.11
1.11
0.92
0.90
0.88
0.86
0.84
6.00
3.79
2.95
3.75
f1 (ppm)

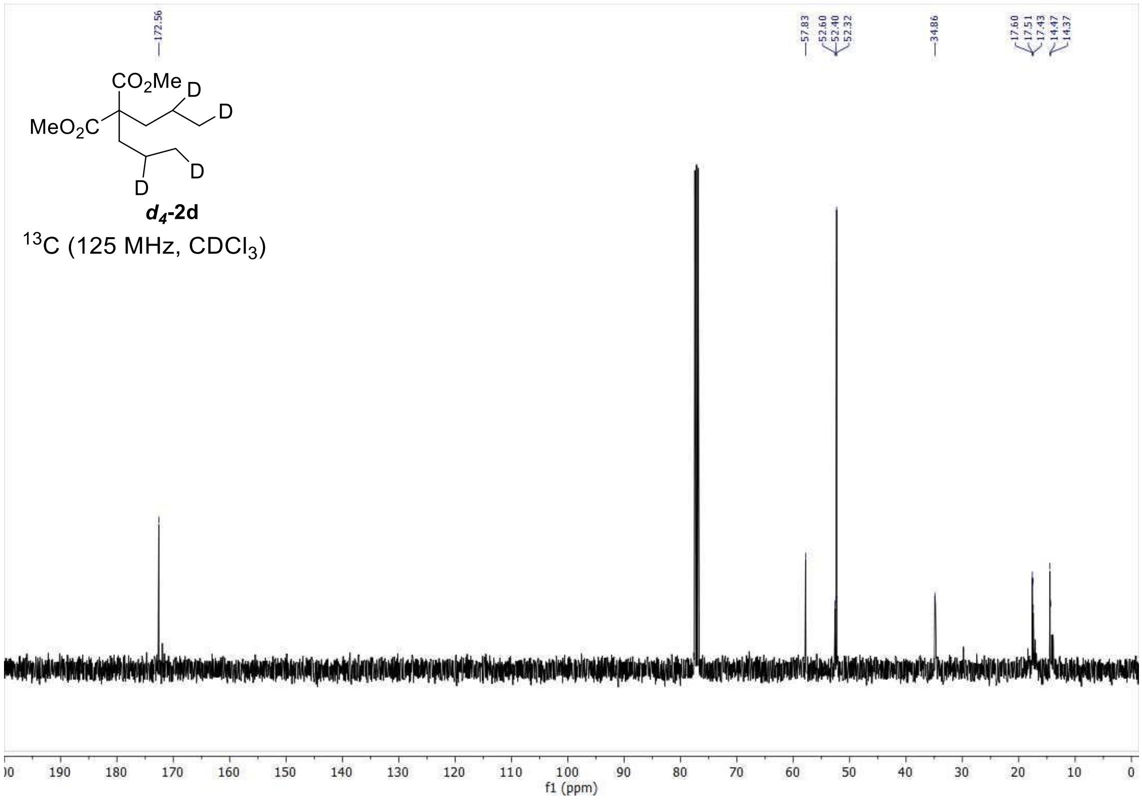

CO2Me
MeO2C
D
D
D
D
d4-2d
13C (125 MHz, CDCl3)
172.56
57.83
52.60
52.40
52.32
34.86
17.60
17.51
17.43
14.47
14.37
f1 (ppm)

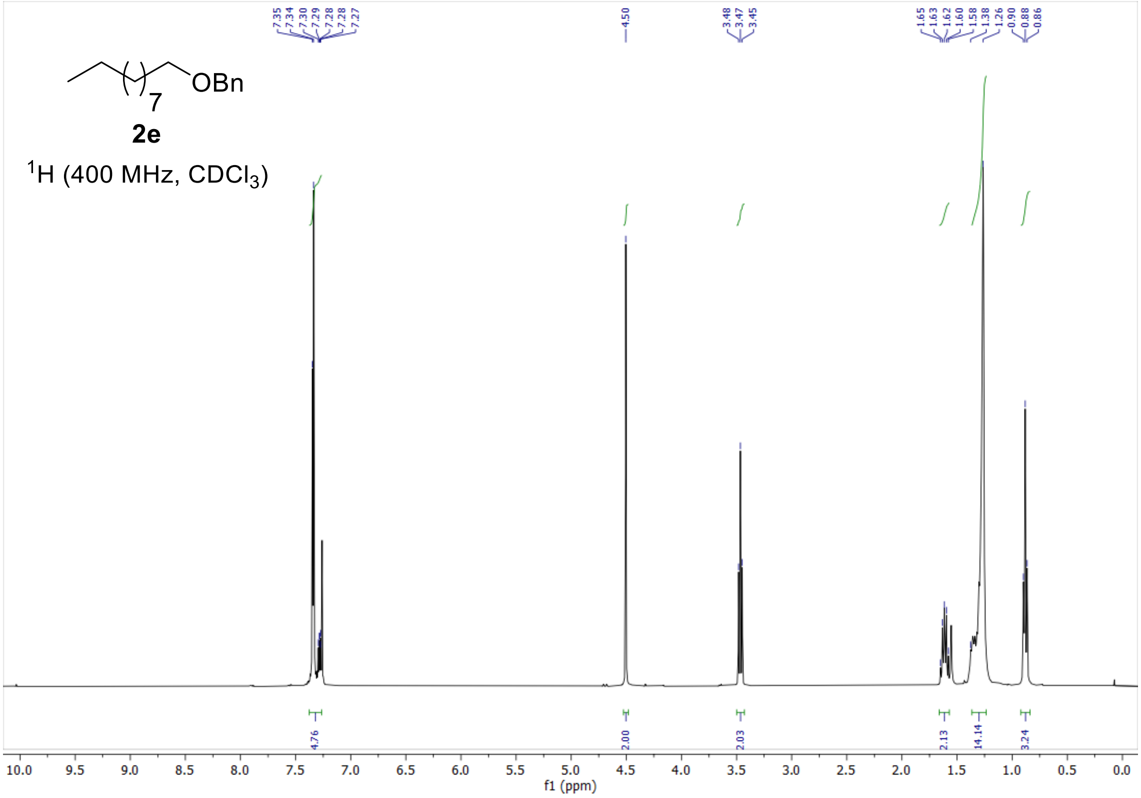
OBn
7
2e
1H (400 MHz, CDCl3)
f1 (ppm)

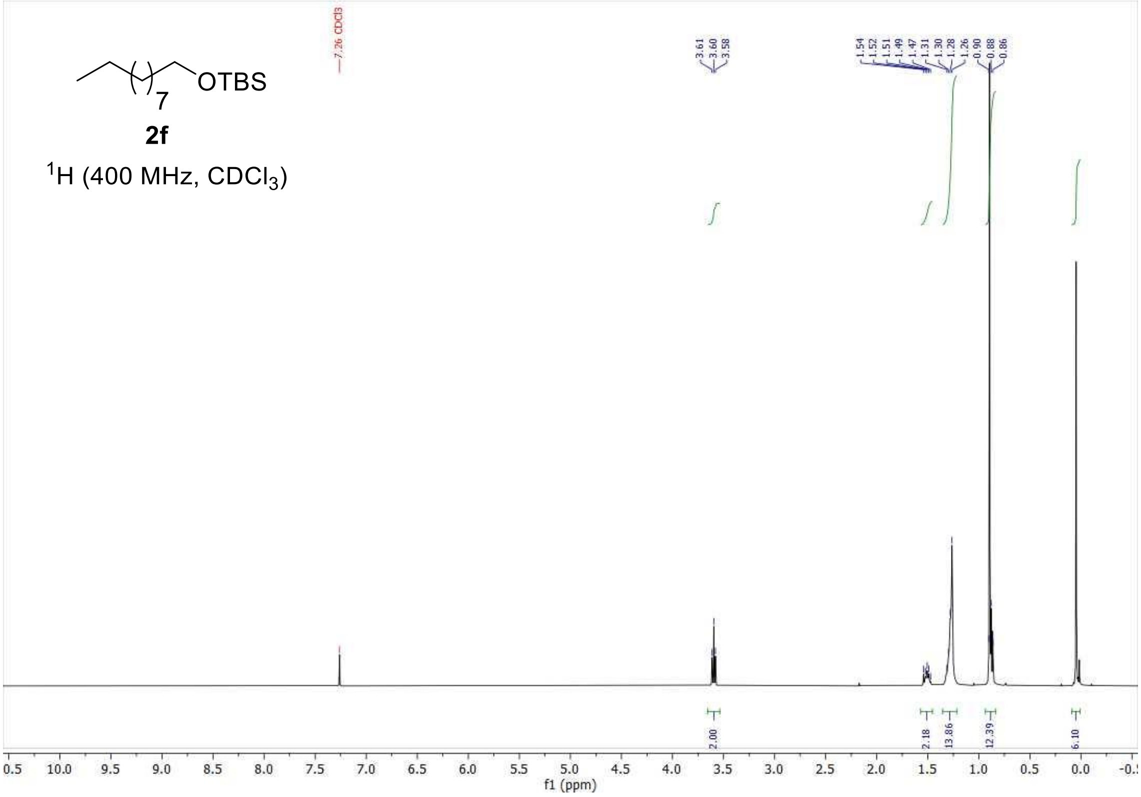
OTBS
7
2f
1H (400 MHz, CDCl3)
7.26 CDCl3
f1 (ppm)

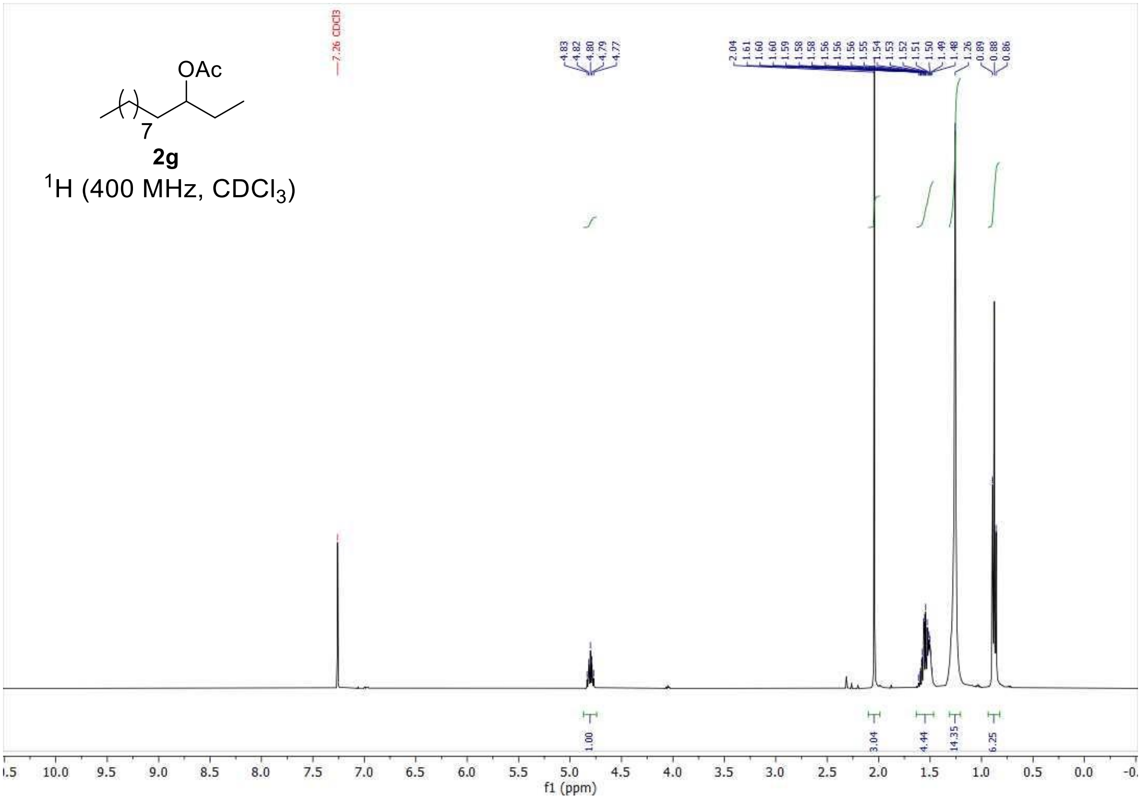

OAc
7
2g
^{1}H (400 MHz, CDCl$_3$)
7.26 CDCl3
4.83
4.82
4.80
4.79
4.77
2.04
1.61
1.60
1.60
1.59
1.58
1.58
1.56
1.56
1.56
1.55
1.54
1.53
1.52
1.51
1.50
1.49
1.48
1.26
0.89
0.88
0.86
1.00
3.04
4.44
14.35
6.25
f1 (ppm)

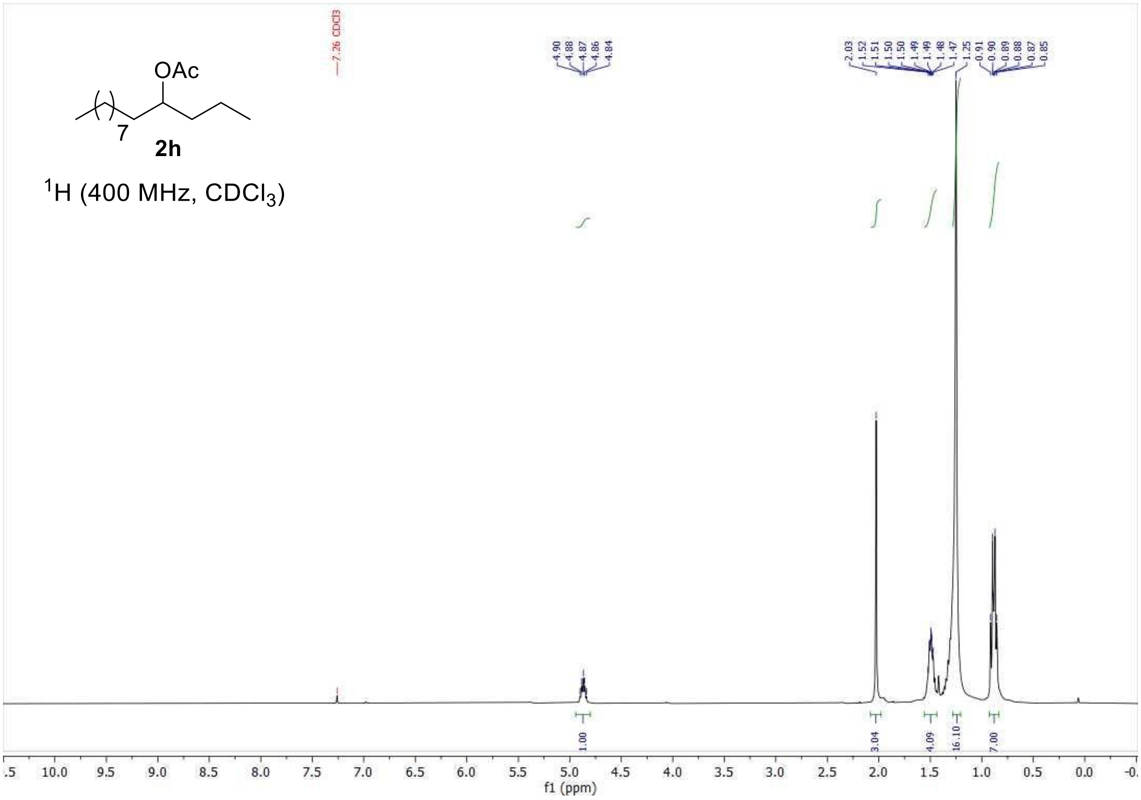
OAc
7
2h
^{1}H (400 MHz, CDCl$_3$)
7.26 CDCl3
4.90 4.88 4.87 4.86 4.84
2.03 1.52 1.51 1.50 1.50 1.49 1.49 1.48 1.47 1.25 0.91 0.90 0.89 0.88 0.87 0.85
1.00
3.04
4.09
16.10
7.00
f1 (ppm)

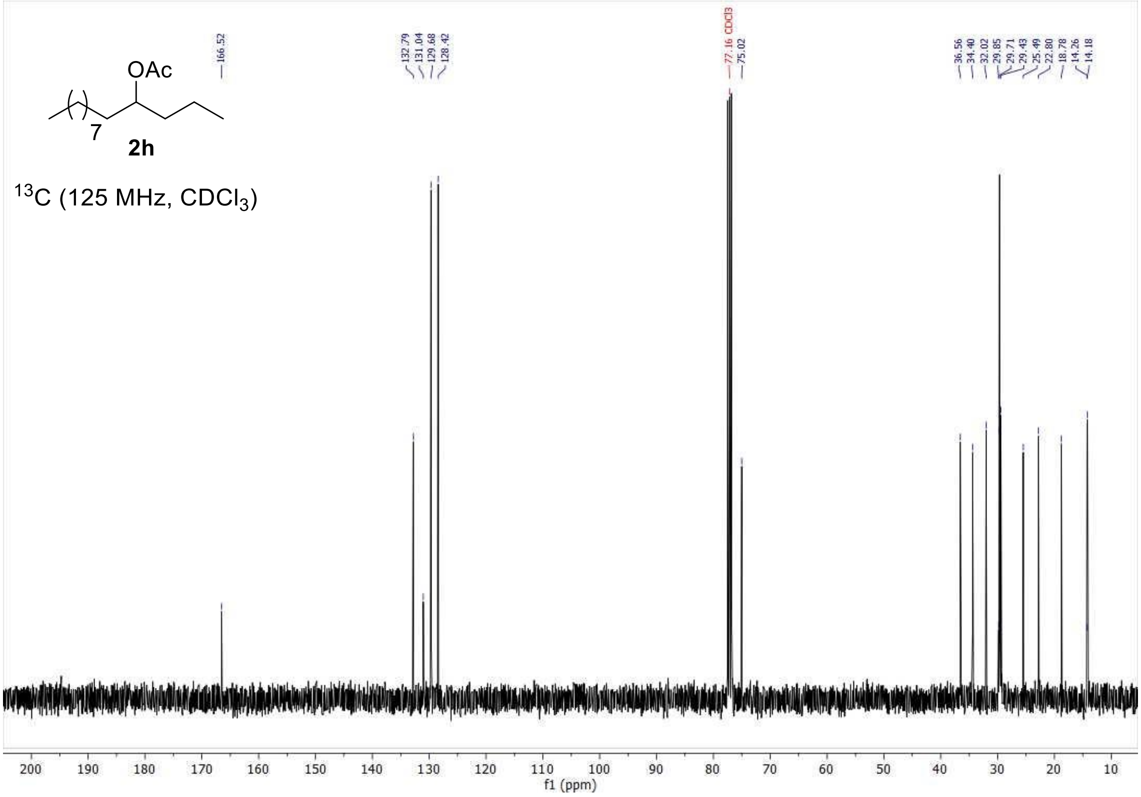
OAc
7
2h
^{13}C (125 MHz, CDCl$_3$)
166.52
132.79 131.04 129.68 128.42
77.16 CDCl3
75.02
36.56 34.40 32.02 29.85 29.71 29.43 25.49 22.80 18.78 14.26 14.18
f1 (ppm)

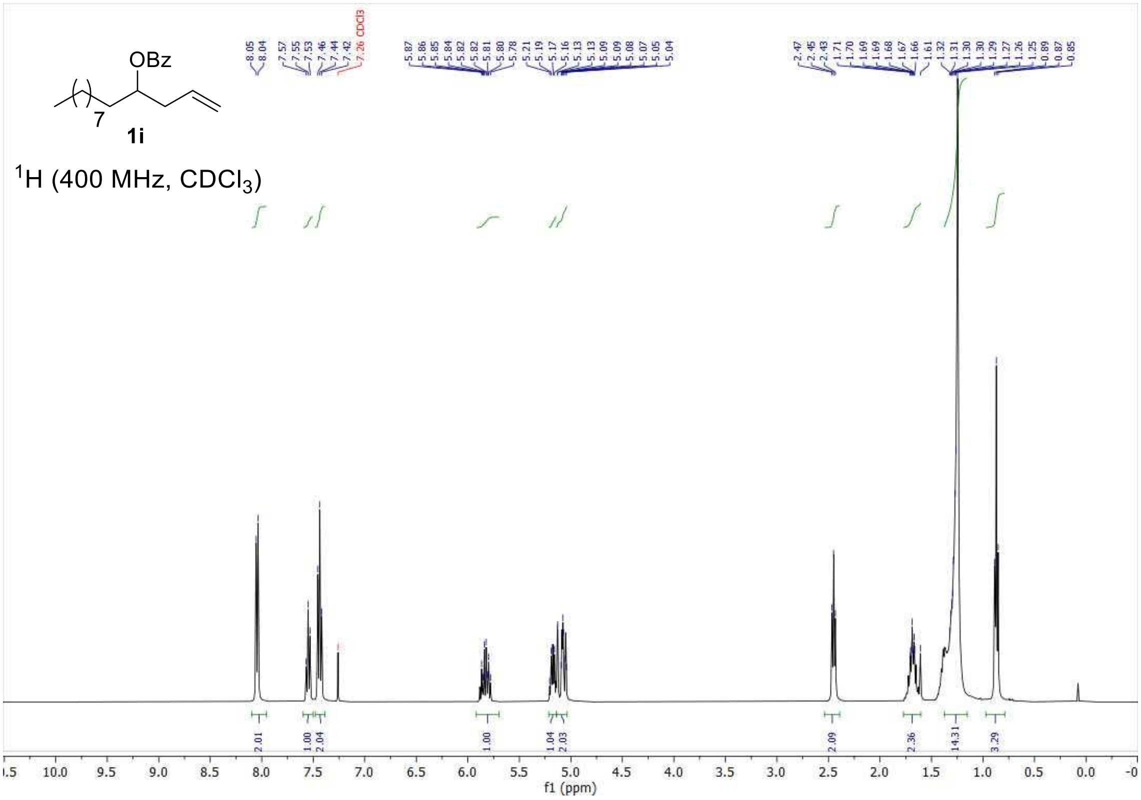
OBz
7
1i
^{1}H (400 MHz, CDCl$_3$)
f1 (ppm)

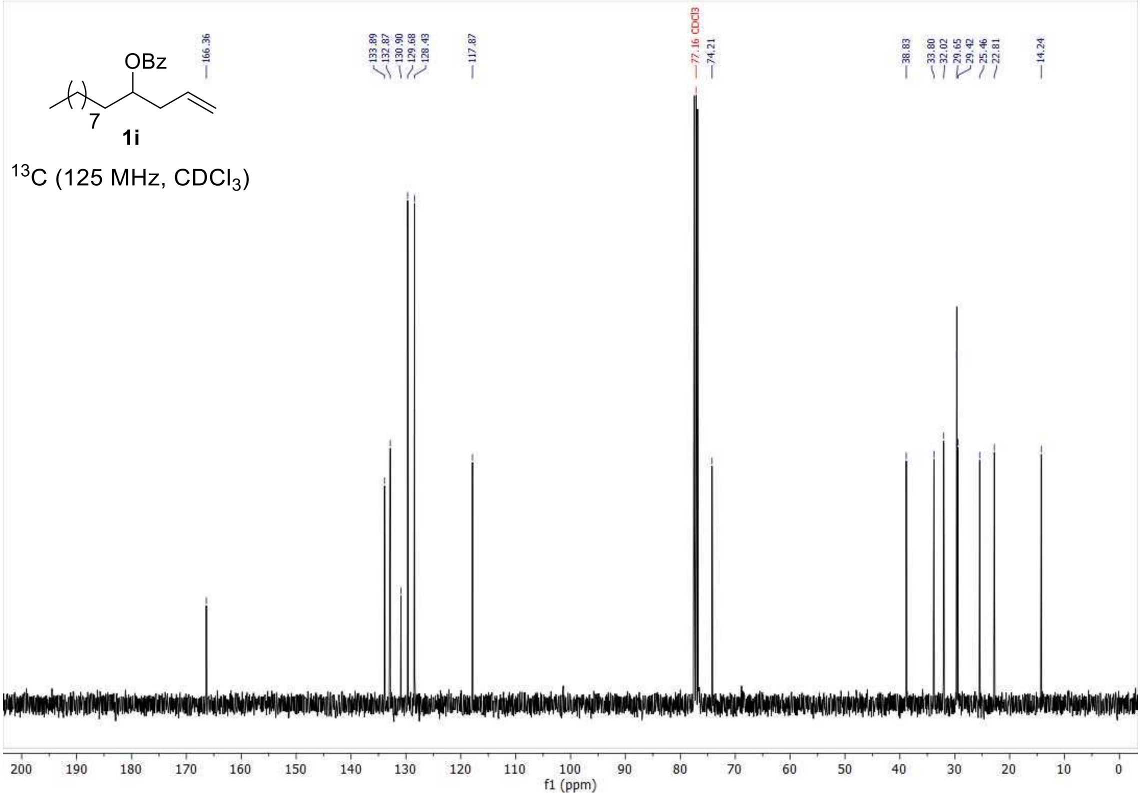
OBz
7
1i
^{13}C (125 MHz, CDCl$_3$)
f1 (ppm)

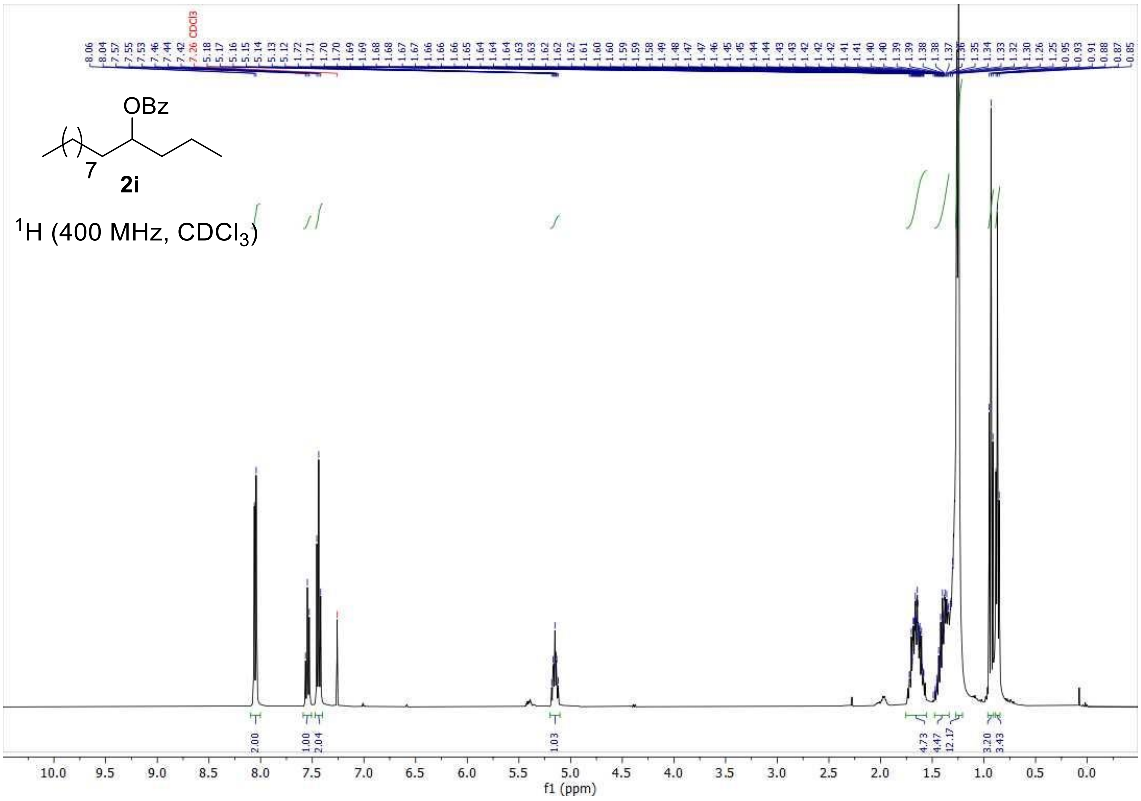
OBz
7
2i
^{1}H (400 MHz, CDCl$_3$)
f1 (ppm)

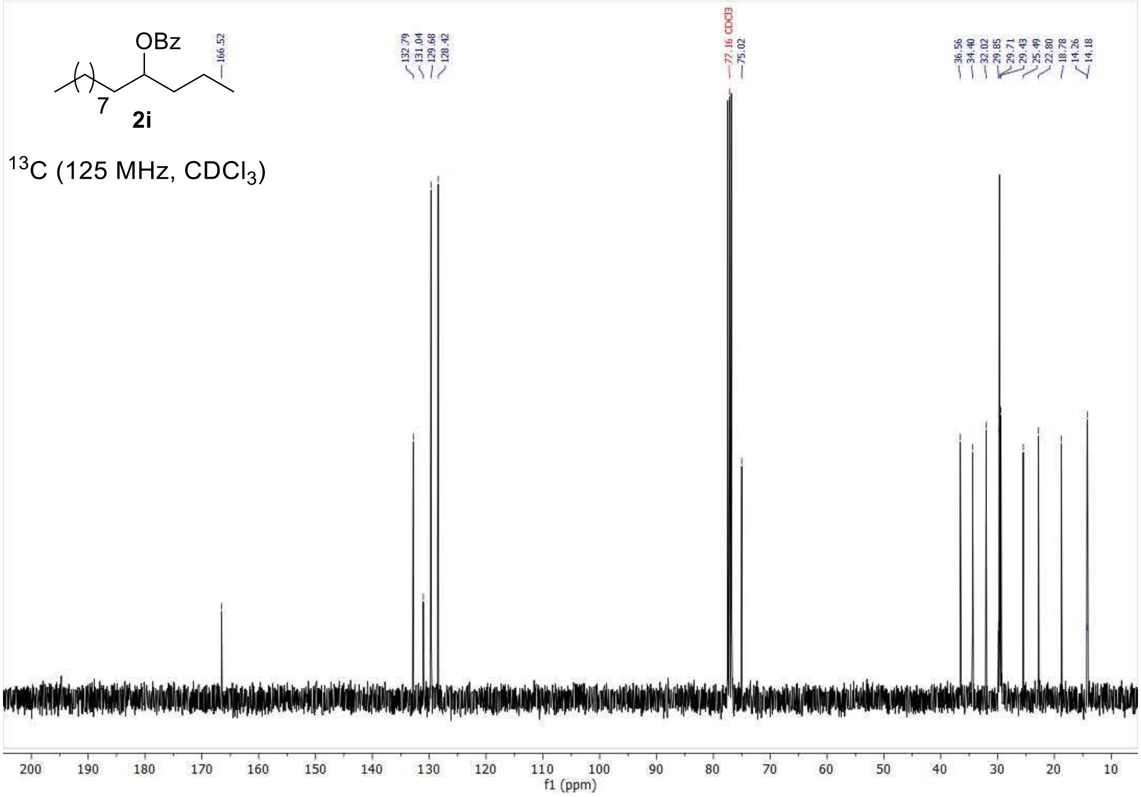
OBz
7
2i
^{13}C (125 MHz, CDCl$_3$)
166.52
132.79
131.04
129.68
128.42
77.16 CDCl3
75.02
36.56
34.40
32.02
29.85
29.71
29.43
25.49
22.80
18.78
14.26
14.18
f1 (ppm)

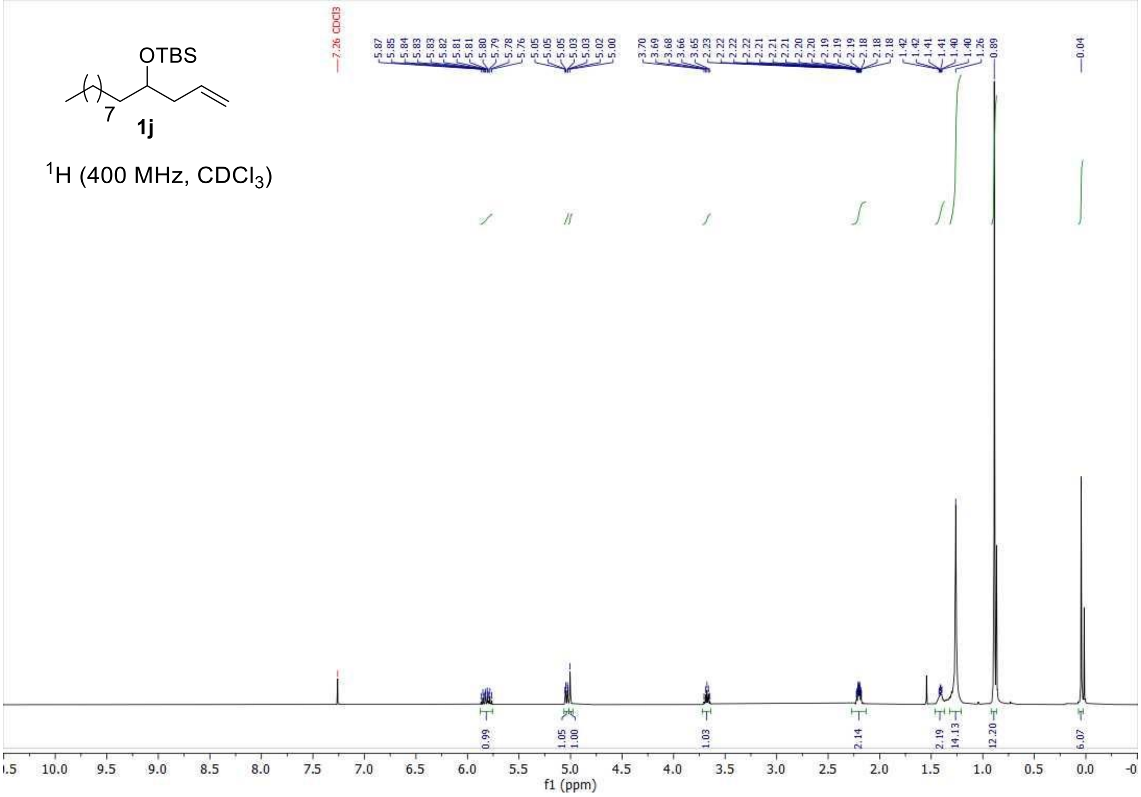

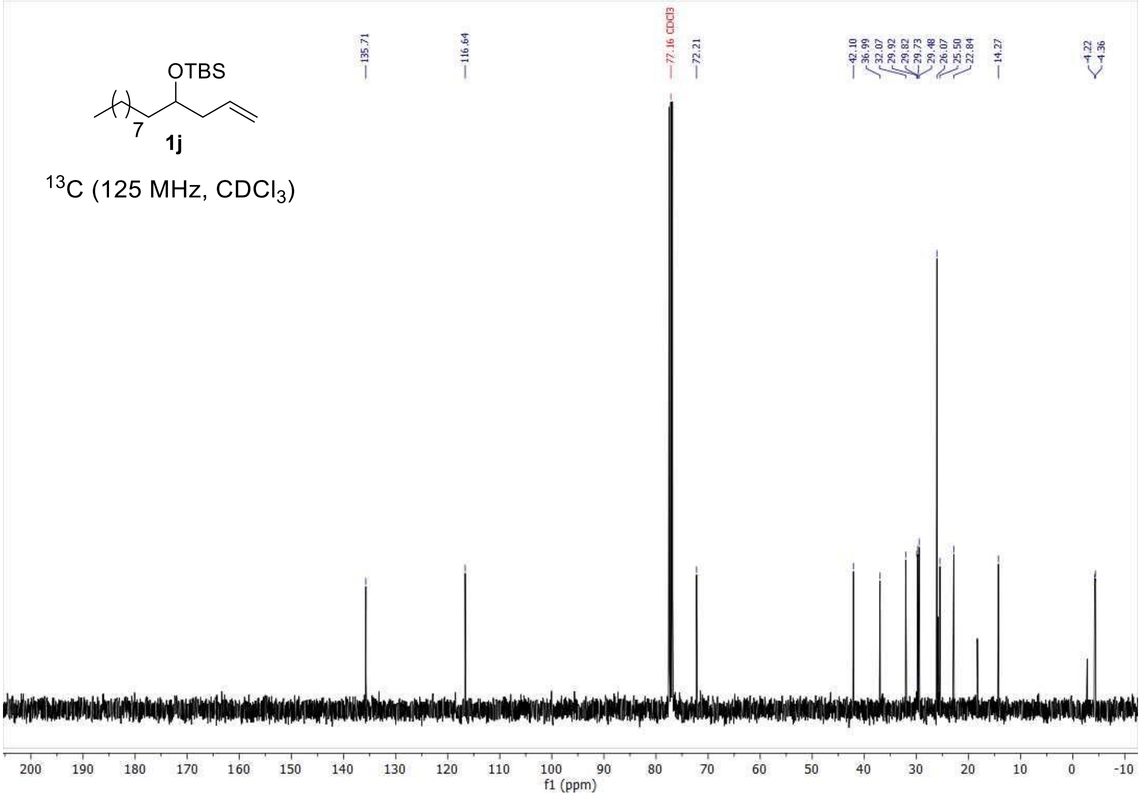

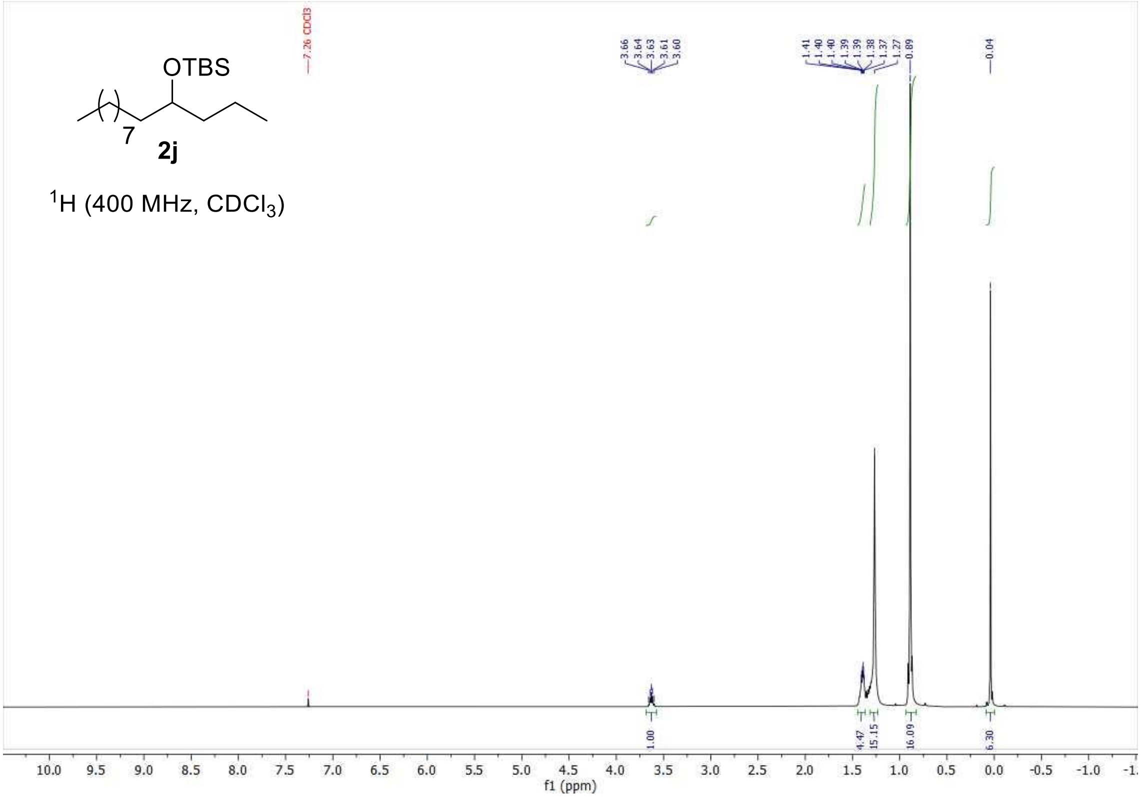
OTBS
7
2j
1H (400 MHz, CDCl3)
7.26 CDCl3
3.66
3.64
3.63
3.61
3.60
1.41
1.40
1.40
1.39
1.39
1.38
1.37
1.27
0.89
0.04
1.00
4.47
15.15
16.09
6.30
f1 (ppm)

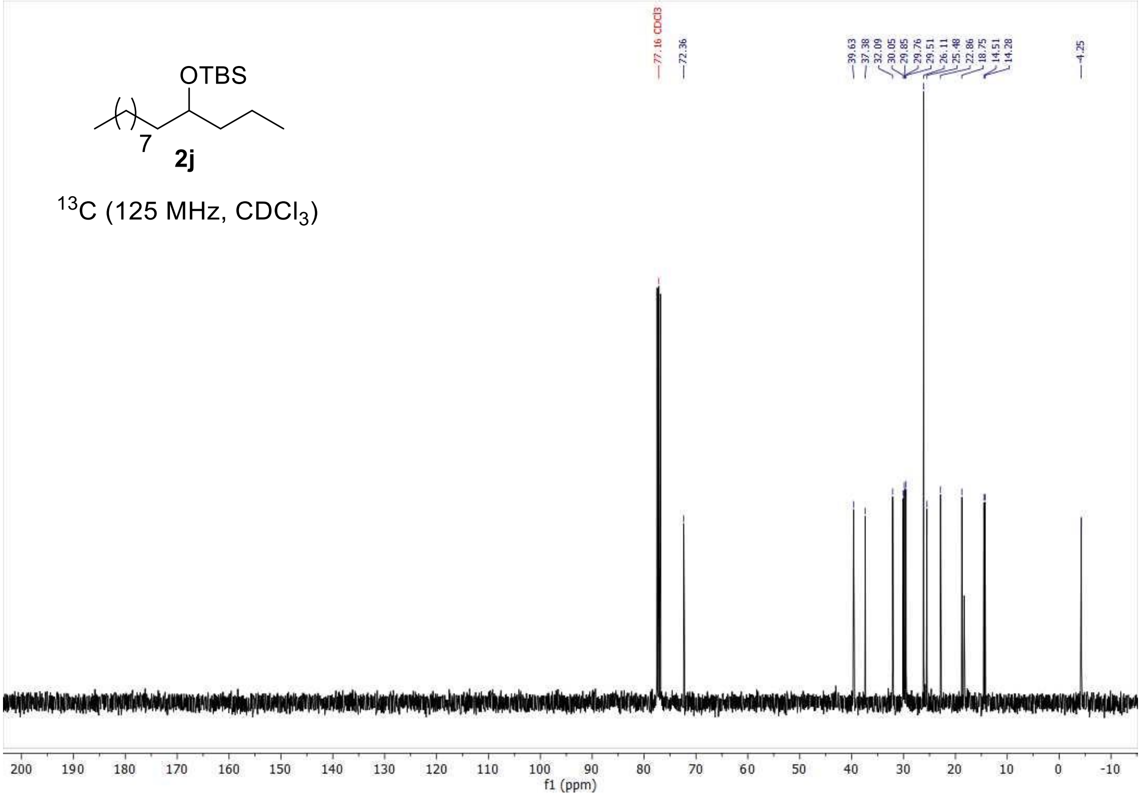
OTBS
7
2j
13C (125 MHz, CDCl3)
77.16 CDCl3
72.36
39.63
37.38
32.09
30.05
29.85
29.76
29.51
26.11
25.48
22.86
18.75
14.51
14.28
-4.25
f1 (ppm)

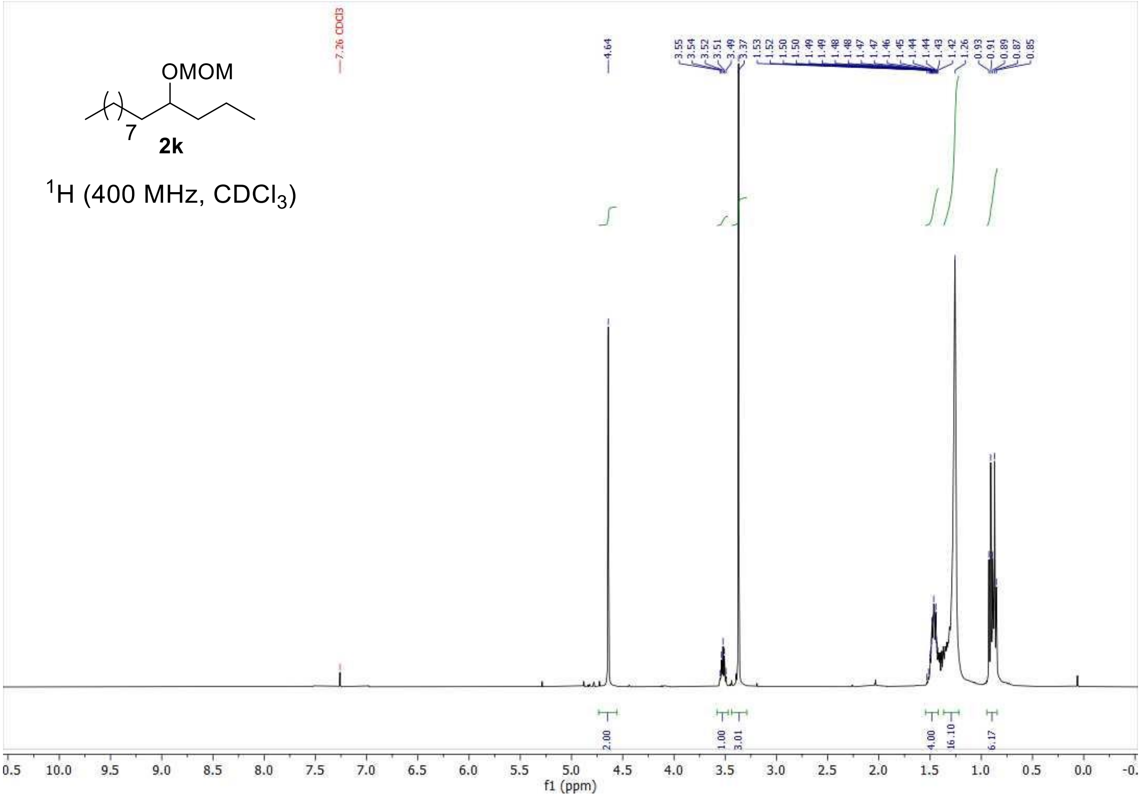

OMOM
7
2k
^{1}H (400 MHz, $CDCl_3$)
f1 (ppm)

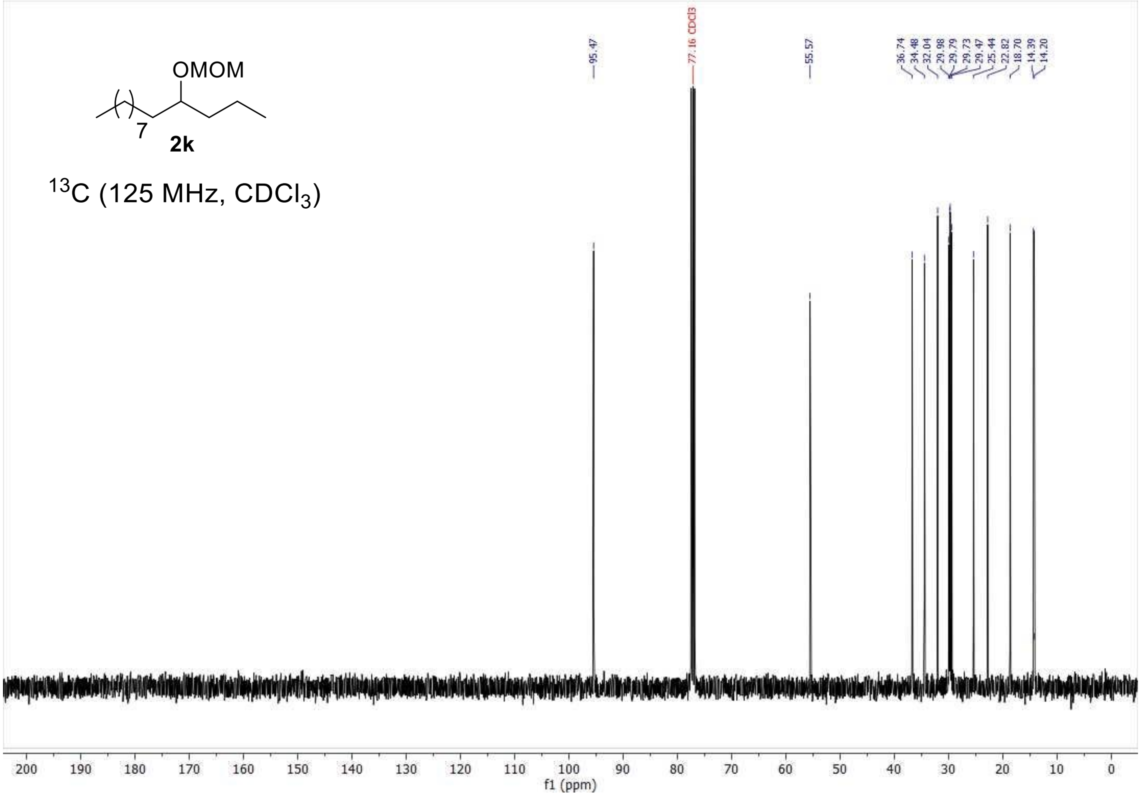

OMOM
7
2k
^{13}C (125 MHz, $CDCl_3$)
f1 (ppm)

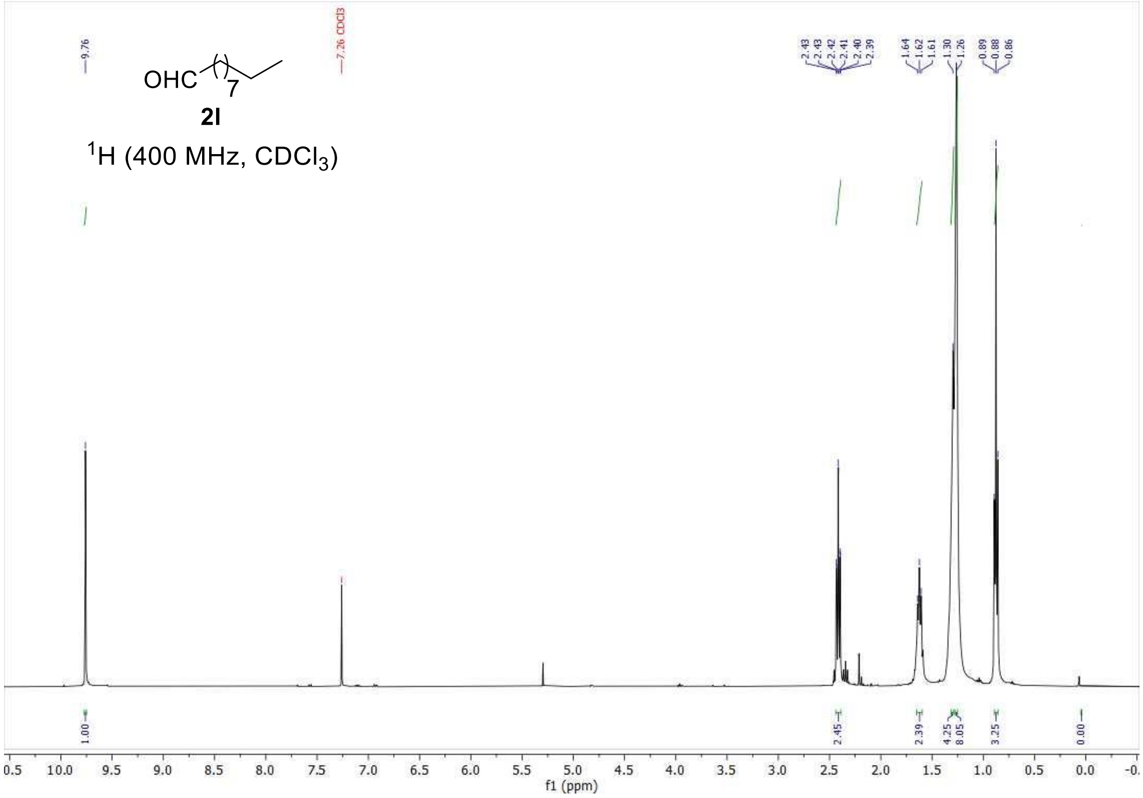
OHC
7
2l
1H (400 MHz, CDCl3)
f1 (ppm)

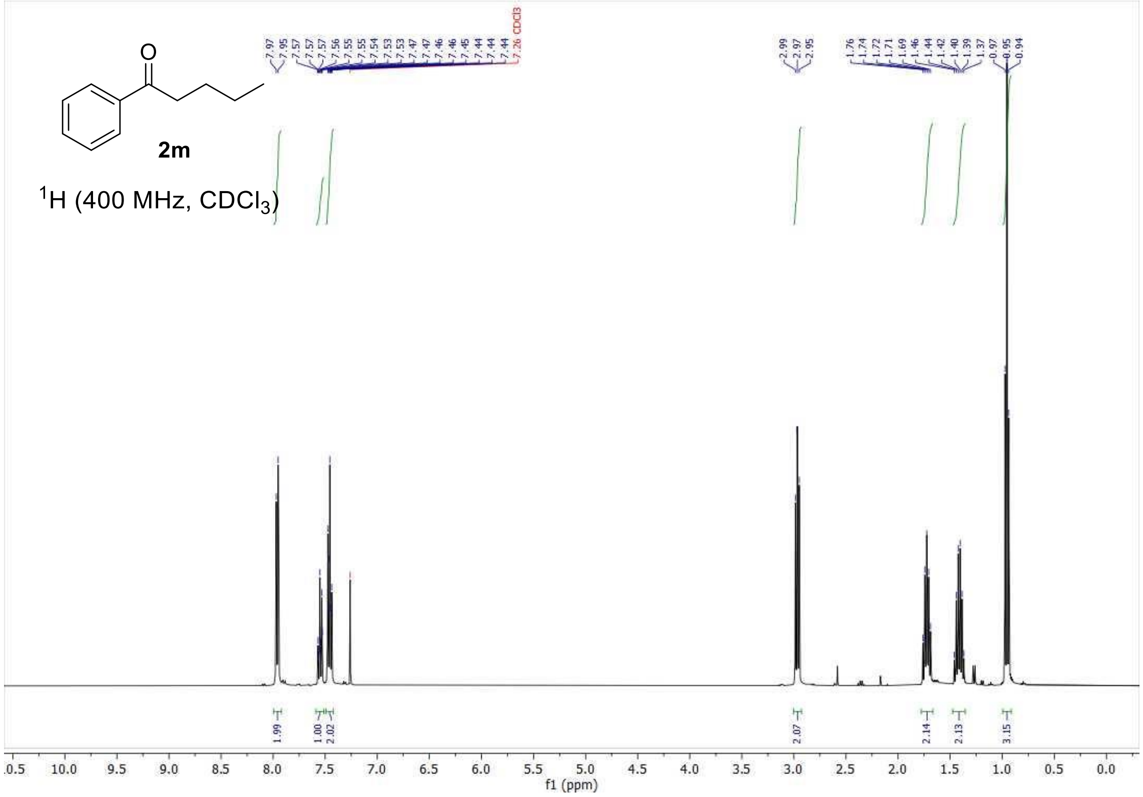
O
2m
1H (400 MHz, CDCl3)
f1 (ppm)

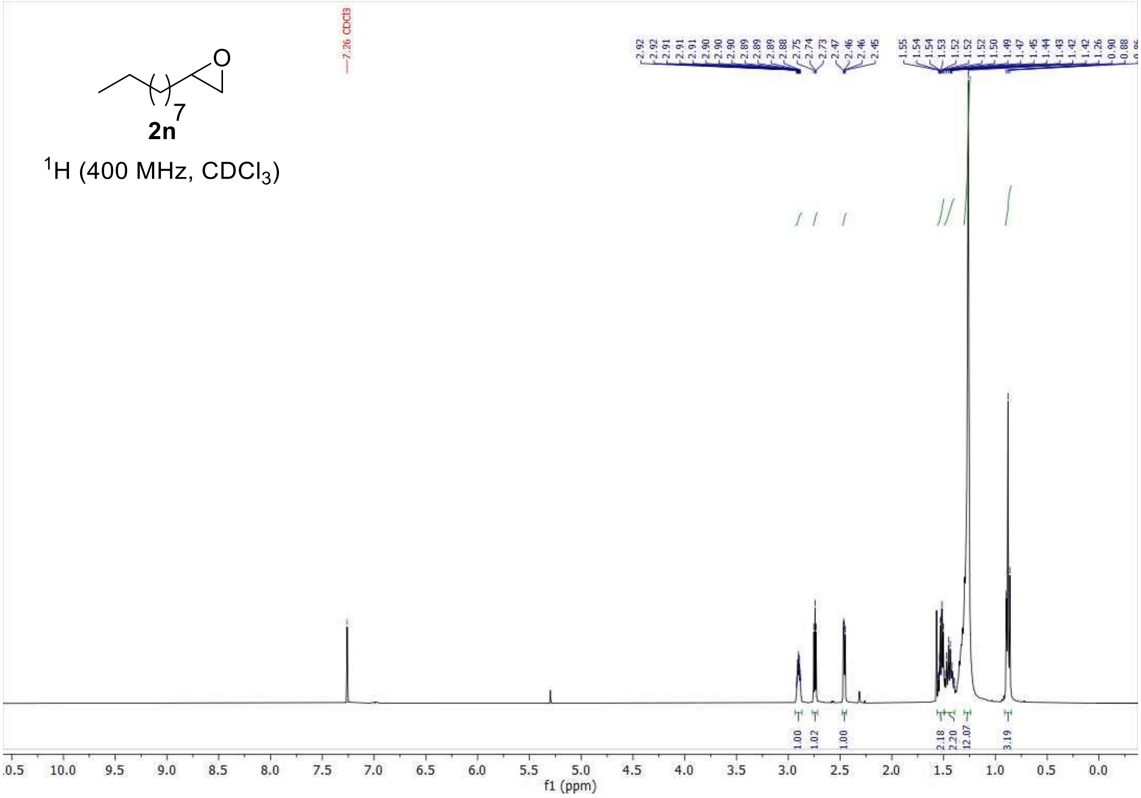
O
7
2n
1H (400 MHz, CDCl3)
7.26 CDCl3
1.00
1.02
1.00
2.18
2.20
12.07
3.19
f1 (ppm)

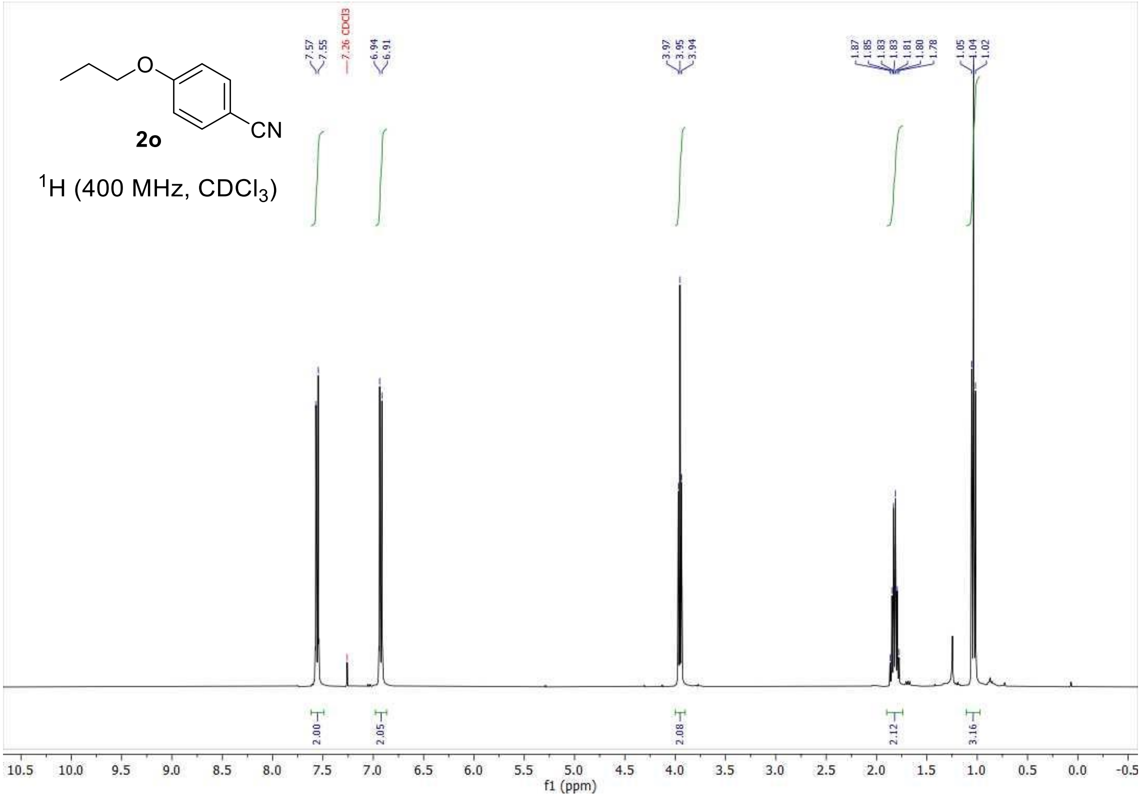
O
CN
2o
1H (400 MHz, CDCl3)
7.57
7.55
7.26 CDCl3
6.94
6.91
3.97
3.95
3.94
1.87
1.85
1.83
1.83
1.81
1.80
1.78
1.05
1.04
1.02
2.00
2.05
2.08
2.12
3.16
f1 (ppm)

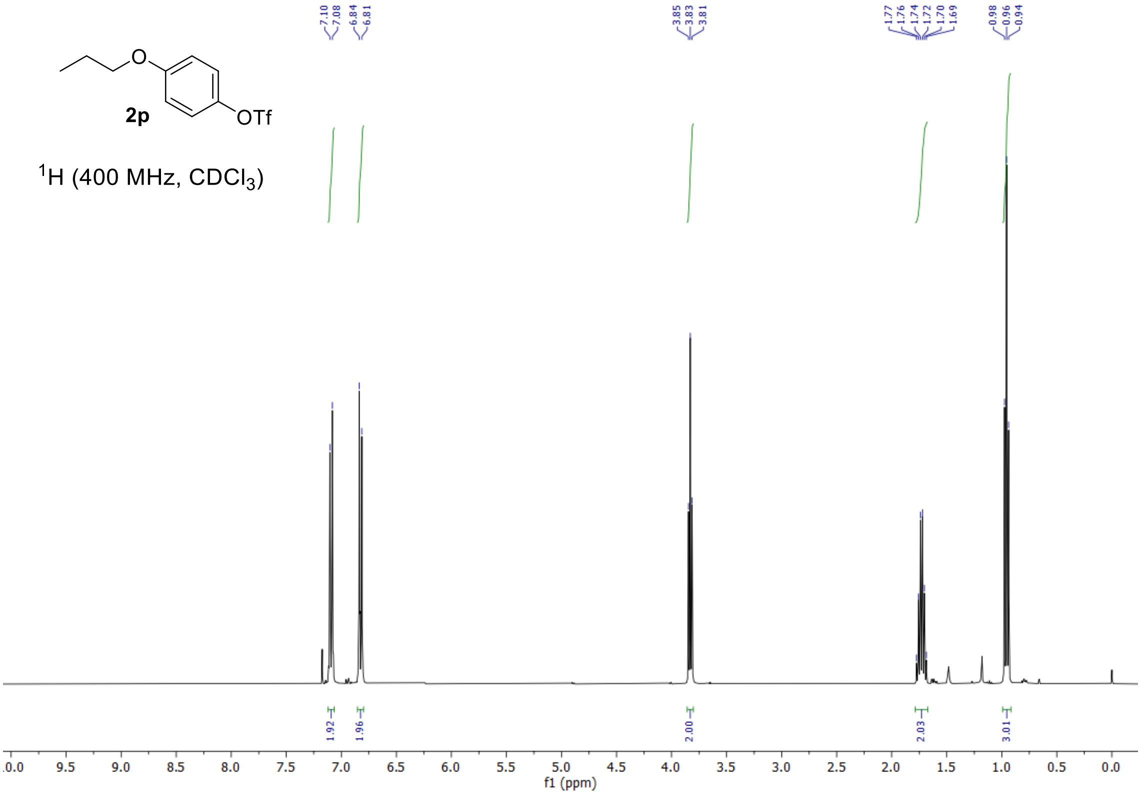
2p
OTf
O
1H (400 MHz, CDCl3)
7.10
7.08
6.84
6.81
3.85
3.83
3.81
1.77
1.76
1.74
1.72
1.70
1.69
0.98
0.96
0.94
1.92
1.96
2.00
2.03
3.01
f1 (ppm)

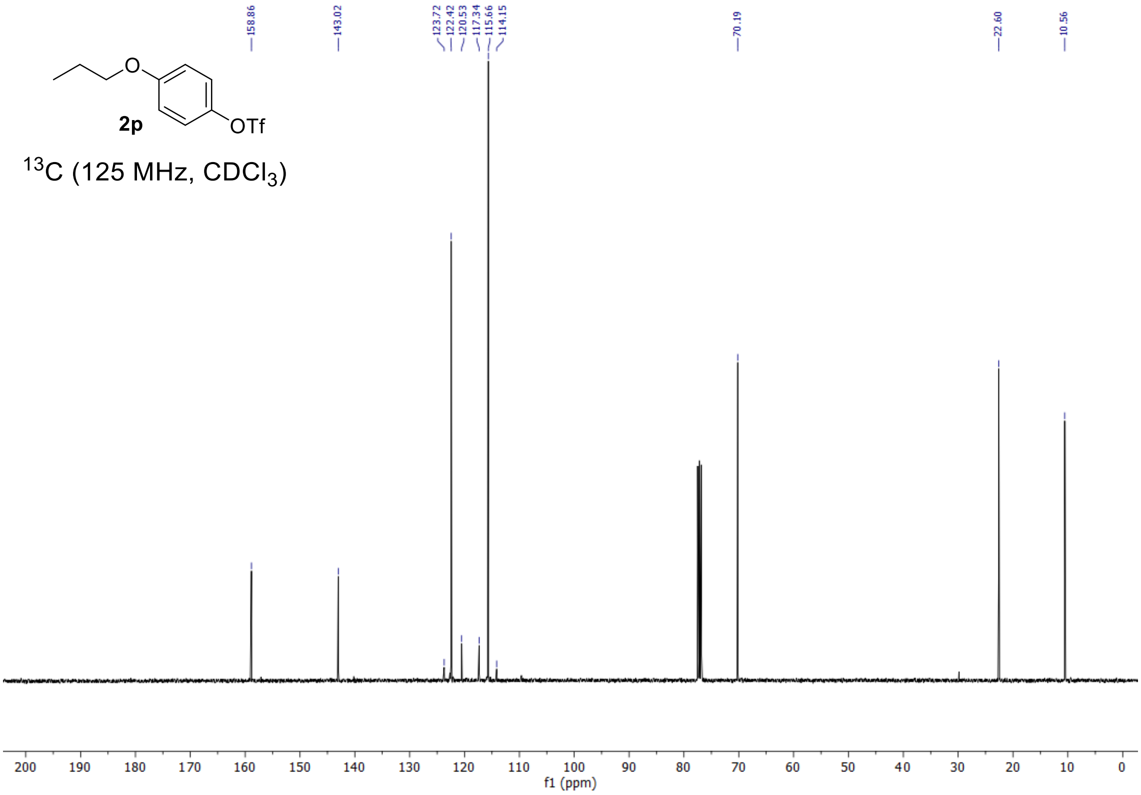
2p
OTf
O
13C (125 MHz, CDCl3)
158.86
143.02
123.72
122.42
120.53
117.34
115.66
114.15
70.19
22.60
10.56
f1 (ppm)

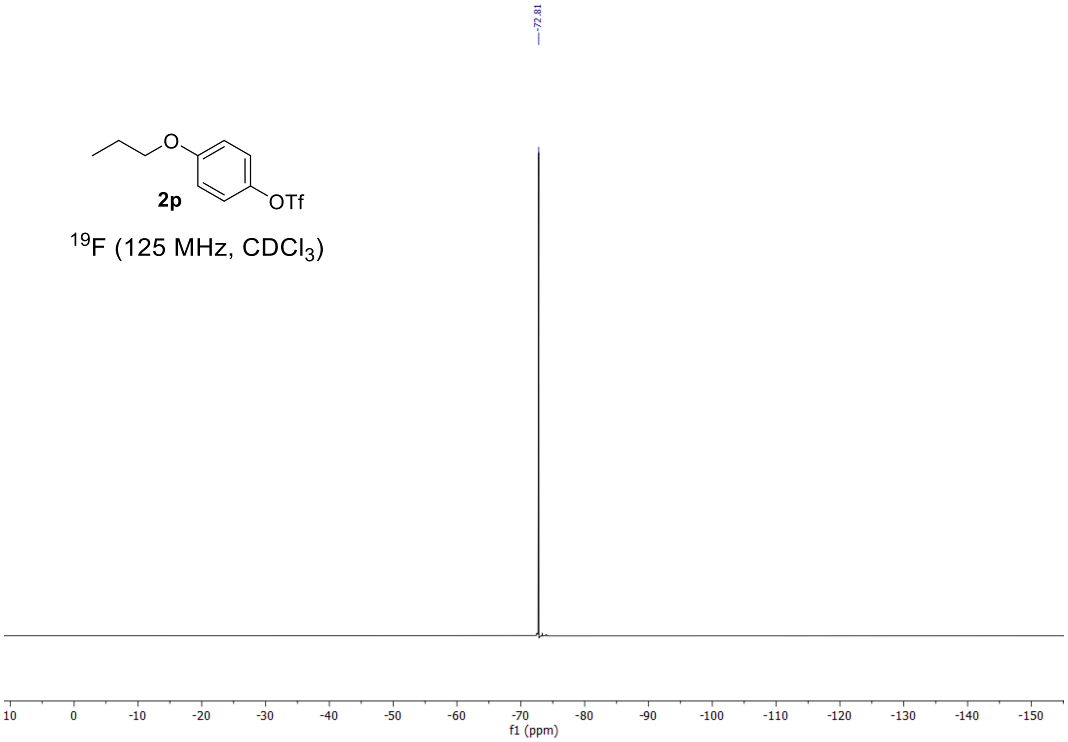
-72.81
O
2p
OTf
^{19}F (125 MHz, CDCl$_3$)
f1 (ppm)

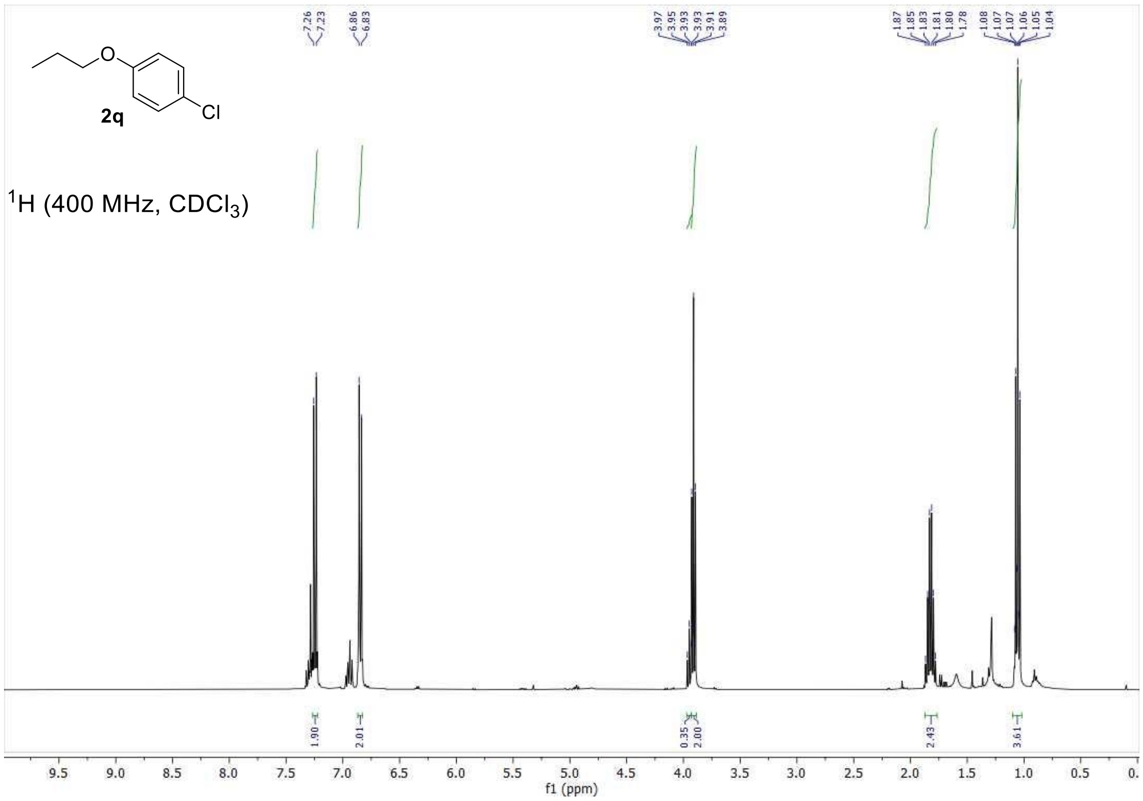
O
2q
Cl
^{1}H (400 MHz, CDCl$_3$)
7.26
7.23
6.86
6.83
3.97
3.95
3.93
3.93
3.91
3.89
1.87
1.85
1.83
1.81
1.80
1.78
1.08
1.07
1.07
1.06
1.05
1.04
1.90
2.01
0.35
2.00
2.43
3.61
f1 (ppm)

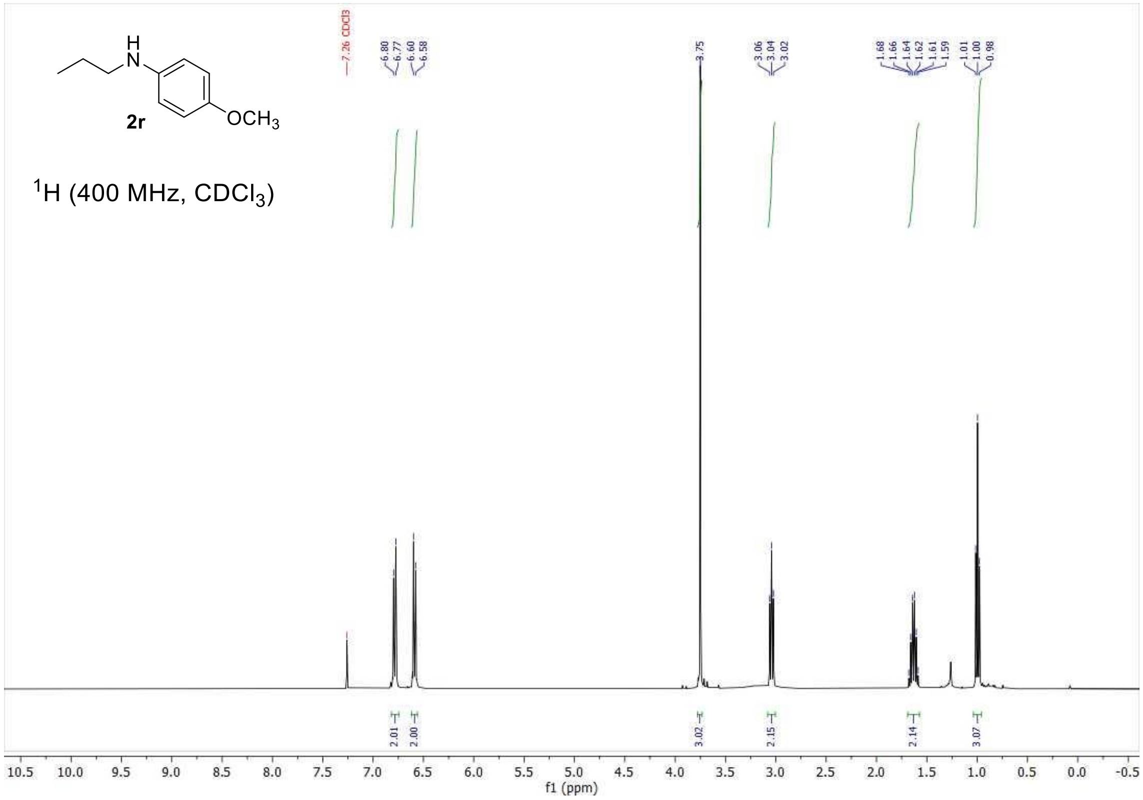
H
N
OCH3
2r
1H (400 MHz, CDCl3)
7.26 CDCl3
6.80
6.77
6.60
6.58
3.75
3.06
3.04
3.02
1.68
1.66
1.64
1.62
1.61
1.59
1.01
1.00
0.98
2.01
2.00
3.02
2.15
2.14
3.07
f1 (ppm)

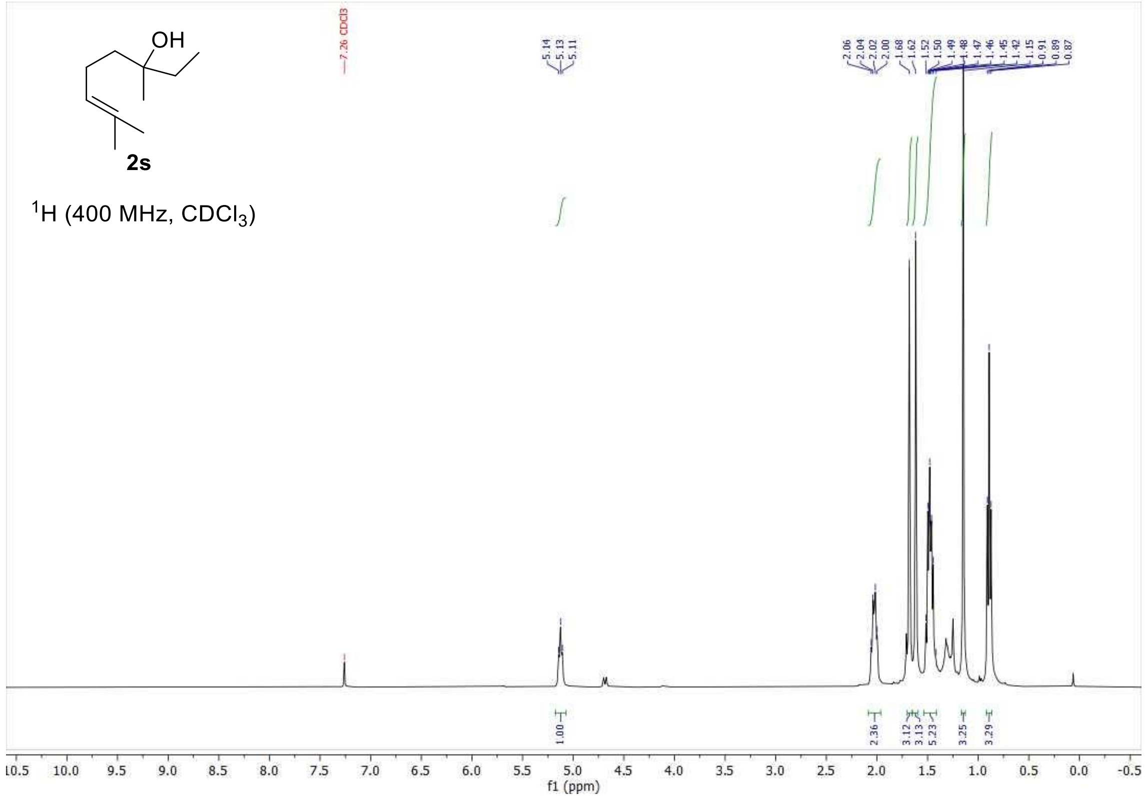
OH
2s
1H (400 MHz, CDCl3)
7.26 CDCl3
5.14
5.13
5.11
2.06
2.04
2.02
2.00
1.68
1.62
1.52
1.50
1.49
1.48
1.47
1.46
1.45
1.42
1.15
0.91
0.89
0.87
1.00
2.36
3.12
3.13
5.23
3.25
3.29
f1 (ppm)

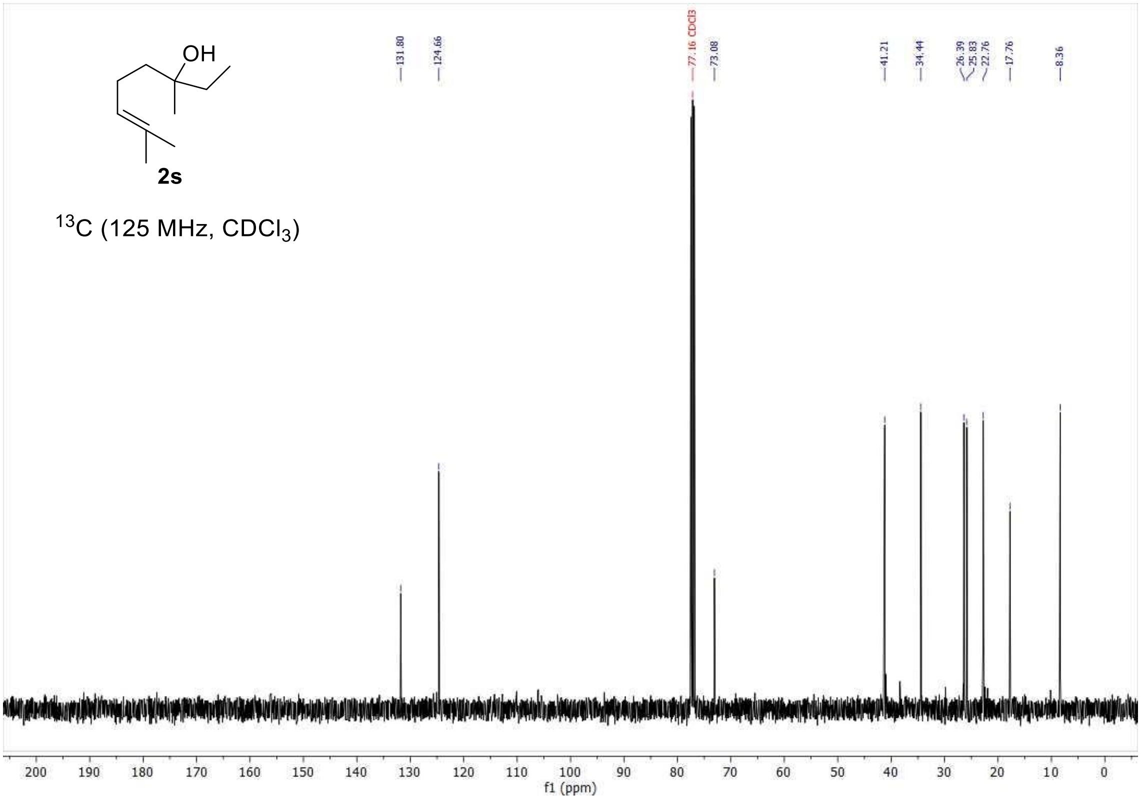
OH
2s
13C (125 MHz, CDCl3)
131.80
124.66
77.16 CDCl3
73.08
41.21
34.44
26.39
25.83
22.76
17.76
8.36
f1 (ppm)

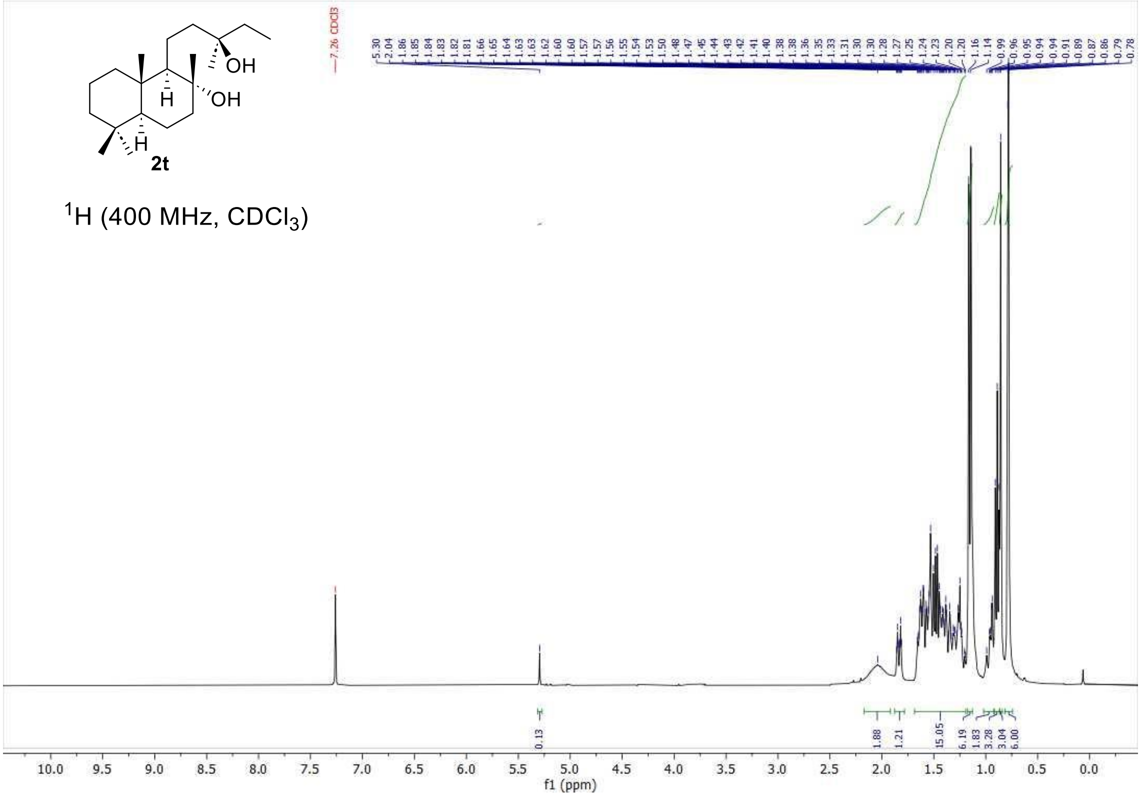
OH
OH
H
H
2t
1H (400 MHz, CDCl3)
7.26 CDCl3
5.30 2.04 1.86 1.85 1.84 1.83 1.82 1.81 1.66 1.65 1.64 1.63 1.63 1.62 1.60 1.57 1.56 1.55 1.54 1.53 1.50 1.48 1.47 1.45 1.44 1.43 1.42 1.41 1.40 1.38 1.38 1.36 1.35 1.33 1.31 1.30 1.28 1.27 1.25 1.24 1.23 1.20 1.16 1.14 0.99 0.96 0.95 0.94 0.94 0.91 0.89 0.87 0.86 0.79 0.78
0.13
1.88
1.21
15.05
6.19
1.83
3.28
3.04
6.00
f1 (ppm)

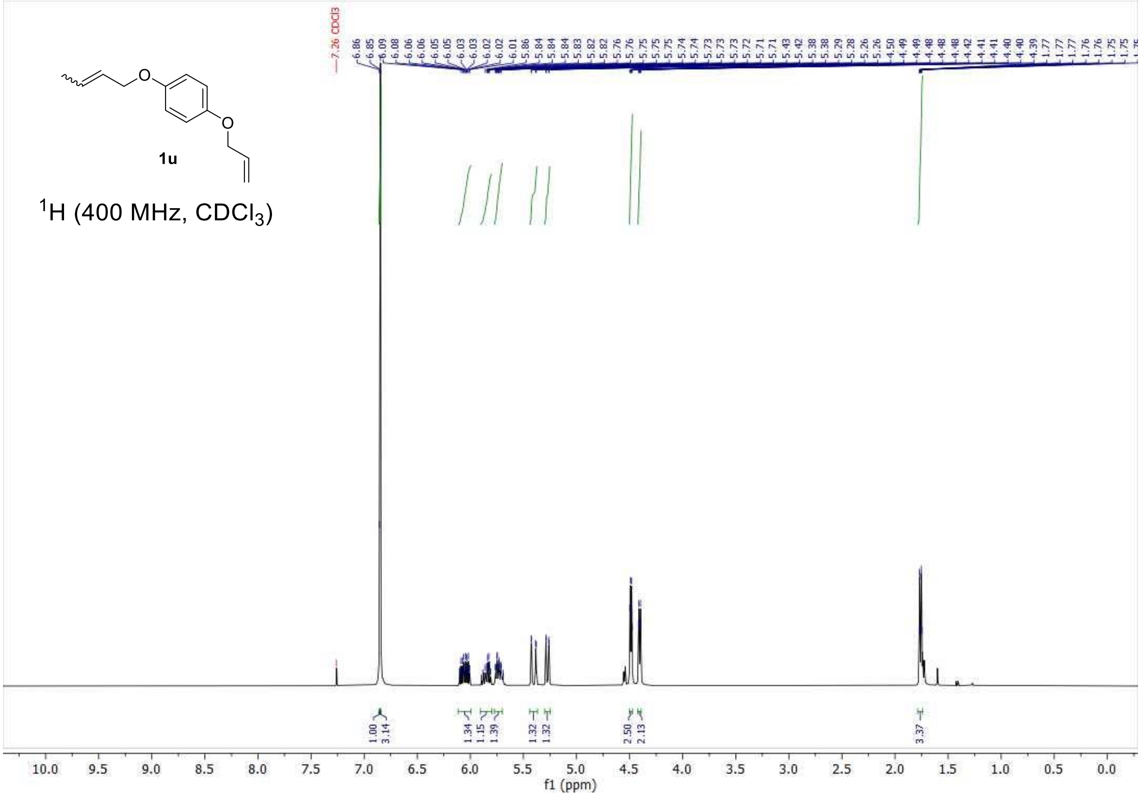

1u
^{1}H (400 MHz, $CDCl_3$)
7.26 CDCl3
f1 (ppm)

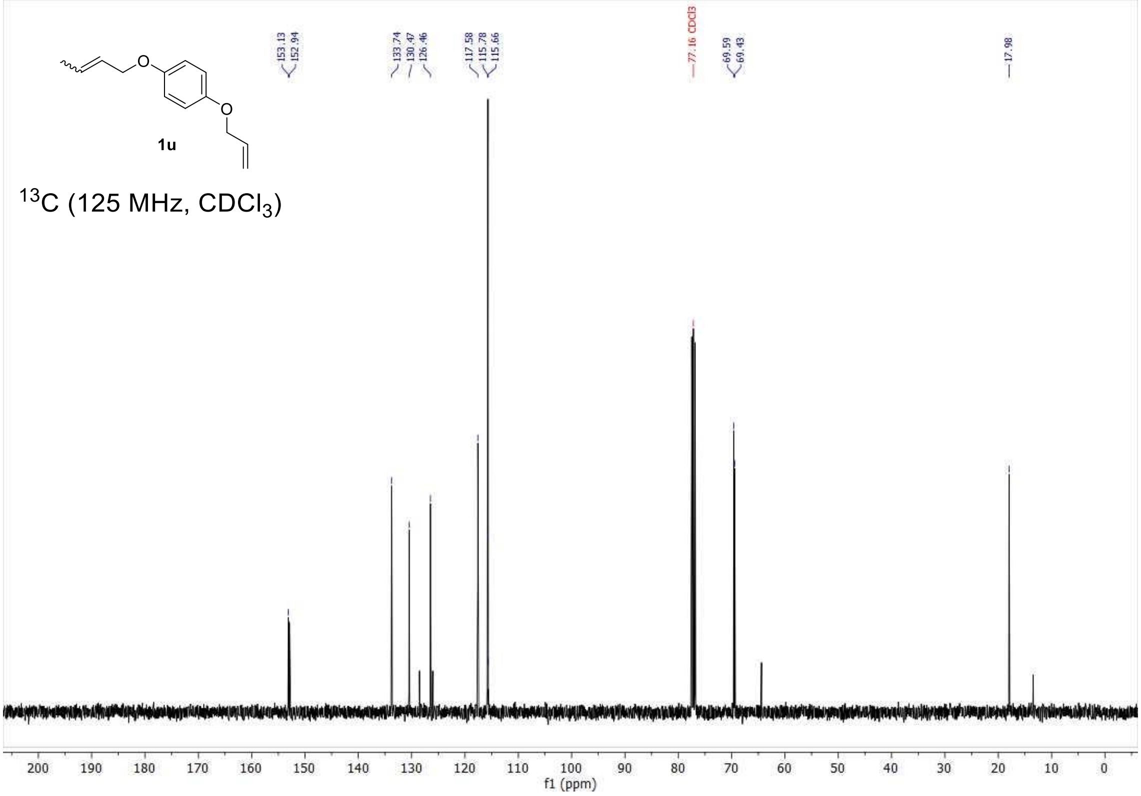

1u
^{13}C (125 MHz, $CDCl_3$)
153.13
152.94
133.74
130.47
126.46
117.58
115.78
115.66
77.16 CDCl3
69.59
69.43
17.98
f1 (ppm)

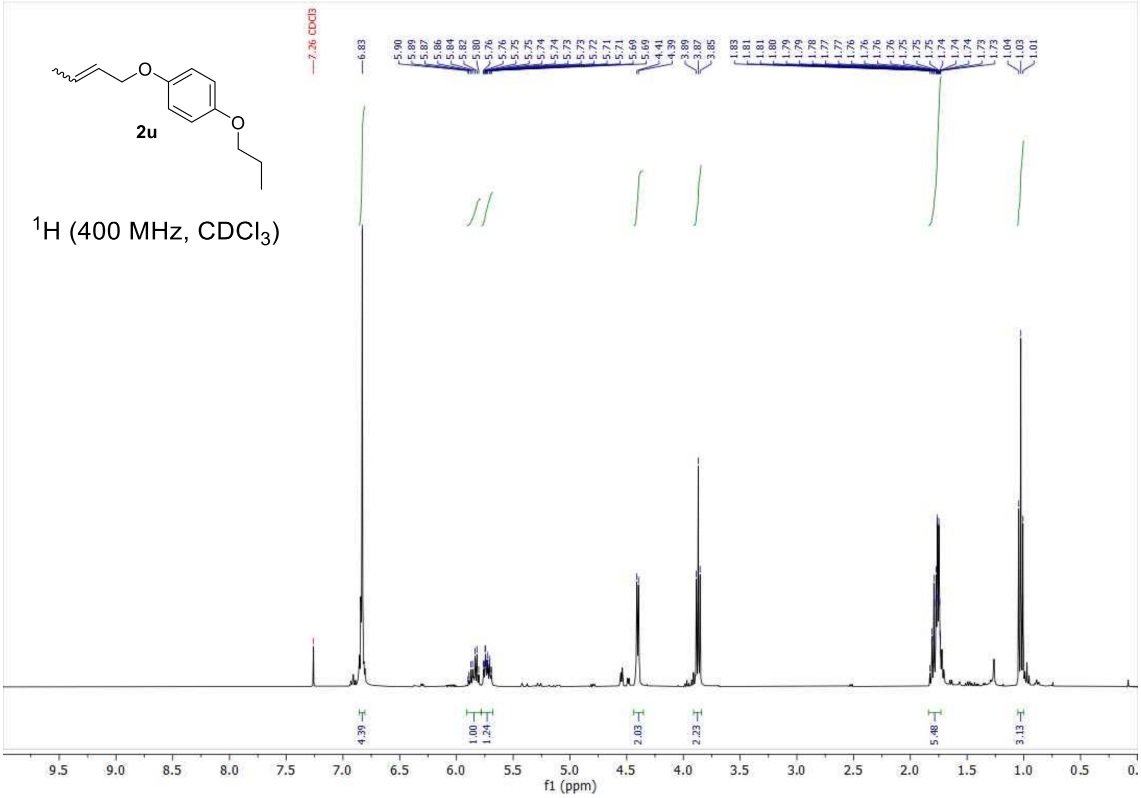
2u
^{1}H (400 MHz, $CDCl_3$)
7.26 CDCl3
6.83
4.39
1.00
1.24
2.03
2.23
5.48
3.13
f1 (ppm)

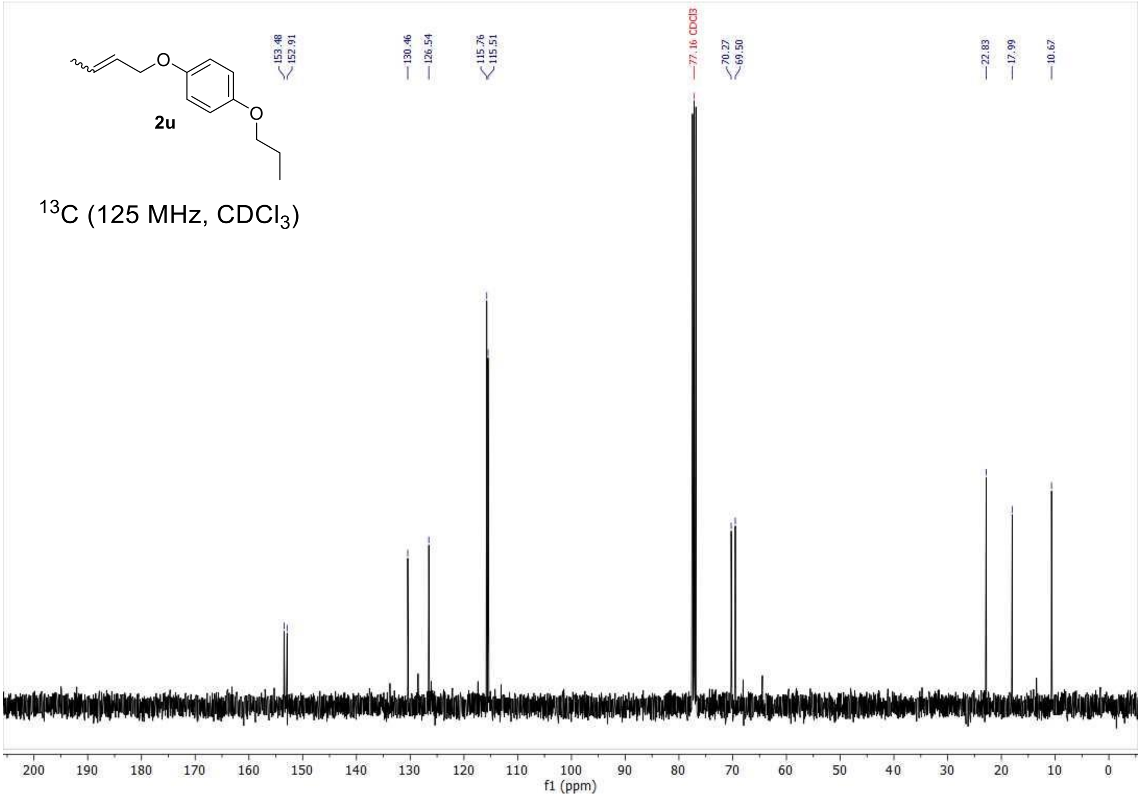
2u
^{13}C (125 MHz, $CDCl_3$)
153.48
152.91
130.46
126.54
115.76
115.51
77.16 CDCl3
70.27
69.50
22.83
17.99
10.67
f1 (ppm)

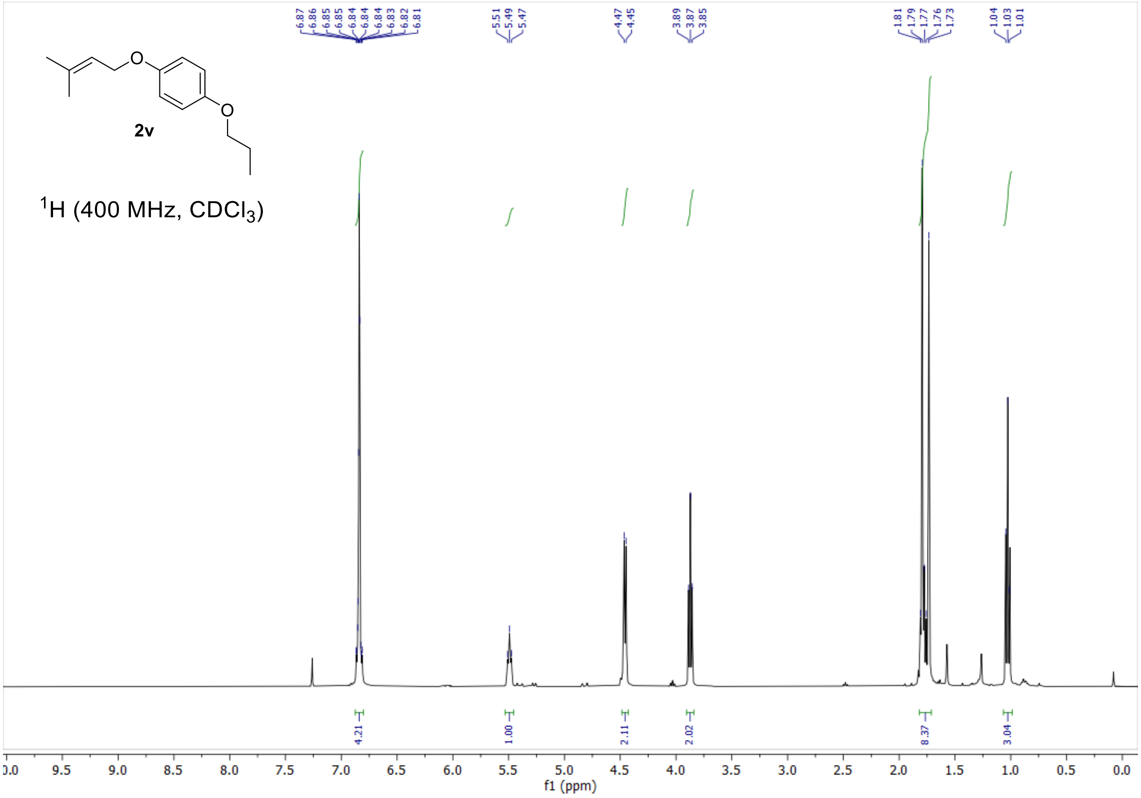
2v
^{1}H (400 MHz, CDCl$_3$)
f1 (ppm)

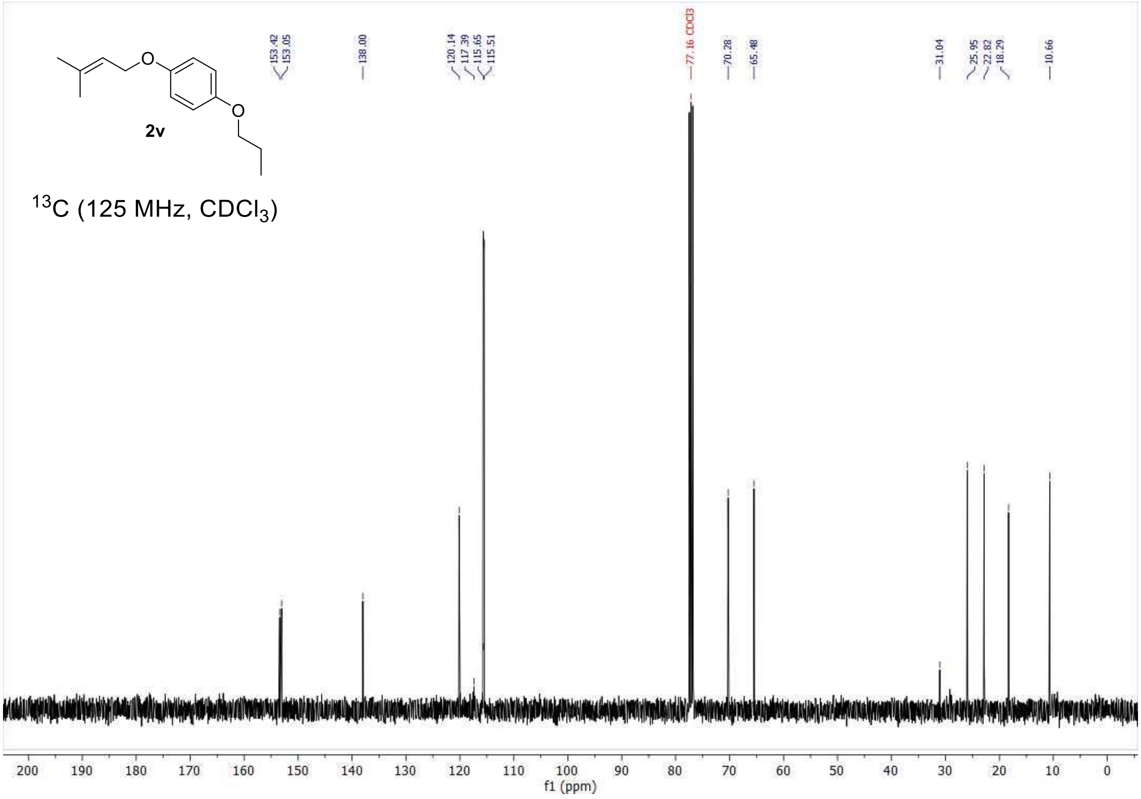
2v
^{13}C (125 MHz, CDCl$_3$)
f1 (ppm)

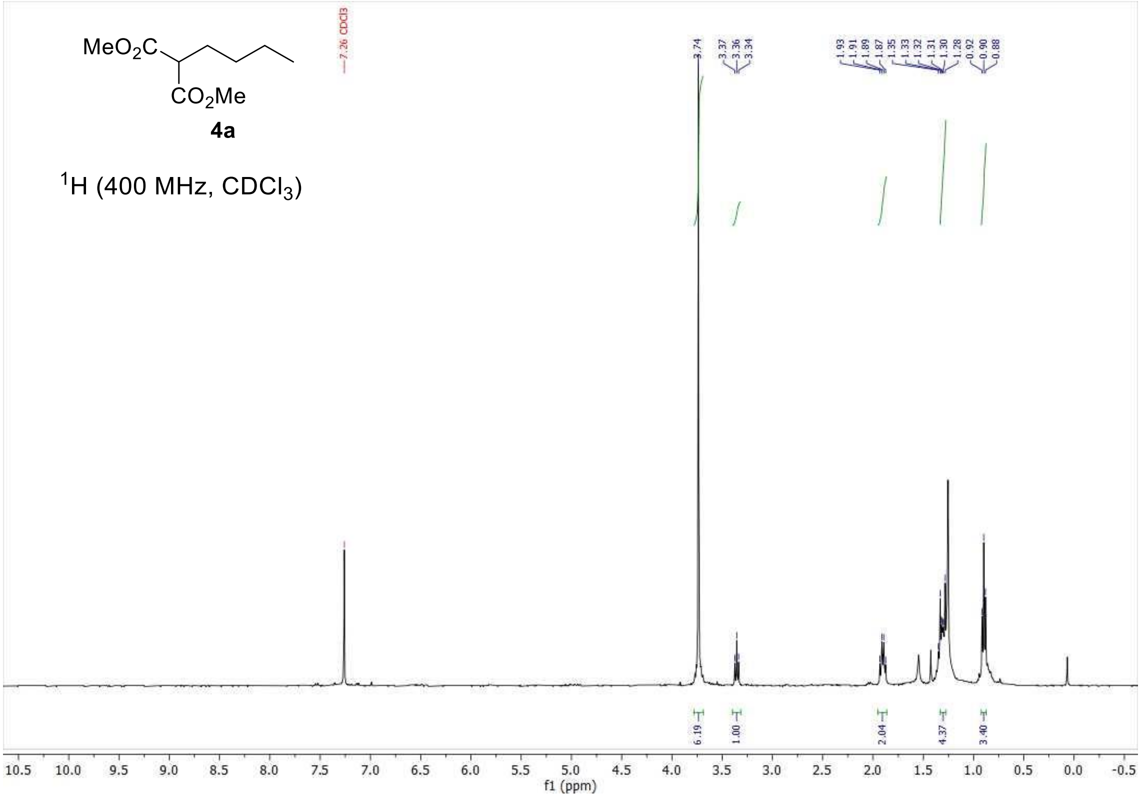
MeO2C
CO2Me
4a
1H (400 MHz, CDCl3)
7.26 CDCl3
3.74
3.37
3.36
3.34
1.93
1.91
1.89
1.87
1.35
1.33
1.32
1.31
1.30
1.28
0.92
0.90
0.88
6.19
1.00
2.04
4.37
3.40
f1 (ppm)

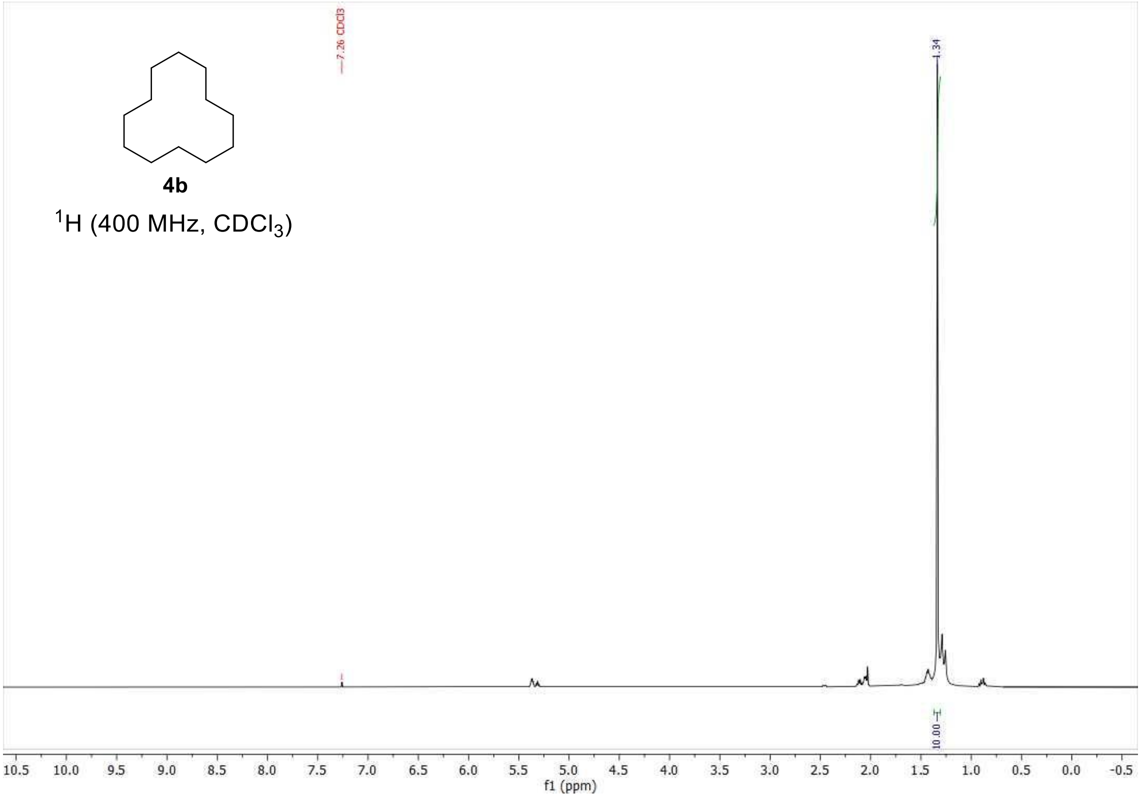
4b
1H (400 MHz, CDCl3)
7.26 CDCl3
1.34
10.00
f1 (ppm)

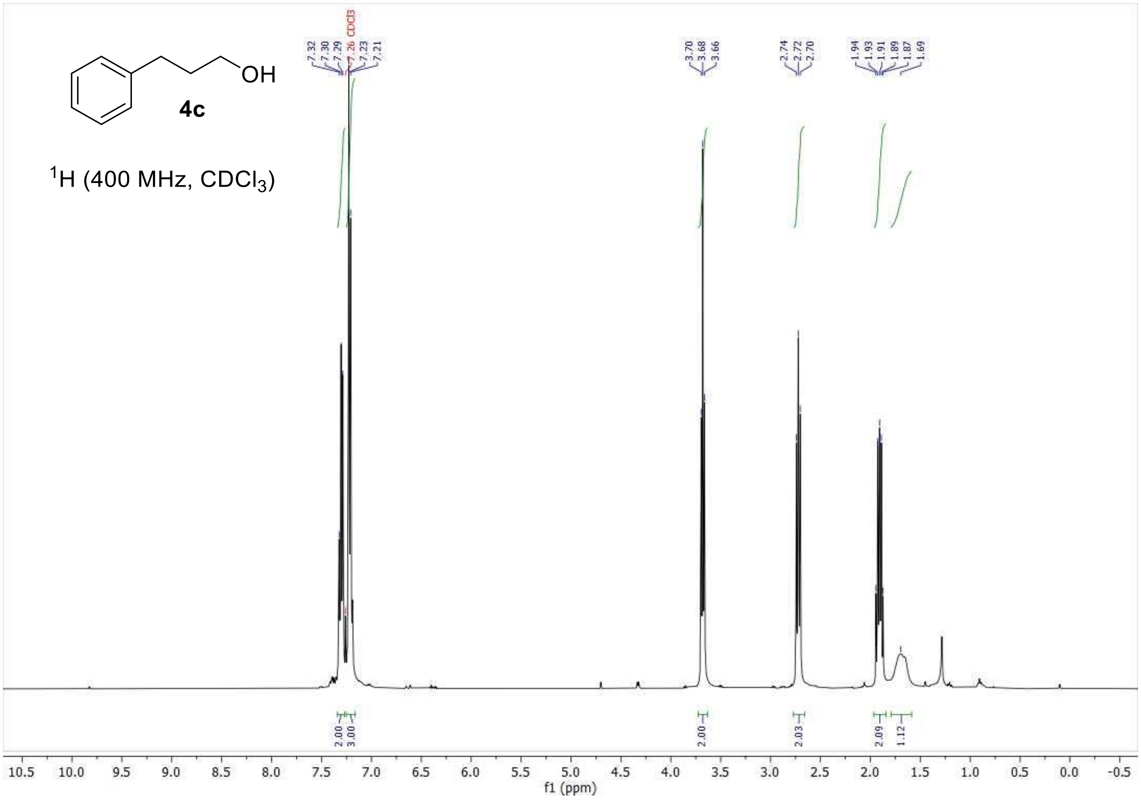

OH
4c
1H (400 MHz, CDCl3)
f1 (ppm)

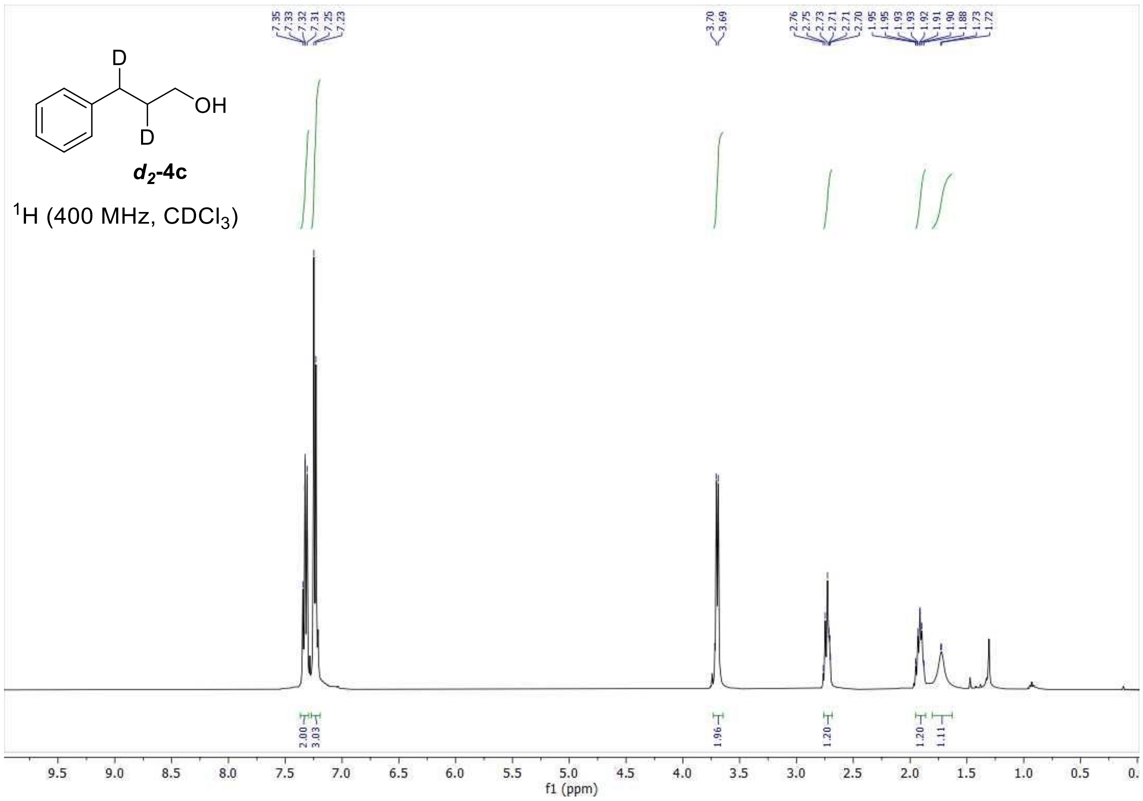

D
OH
D
d_2-4c
^{1}H (400 MHz, $CDCl_3$)
7.35
7.33
7.32
7.31
7.25
7.23
3.70
3.69
2.76
2.75
2.73
2.71
2.71
2.70
1.95
1.95
1.93
1.93
1.92
1.91
1.90
1.88
1.73
1.72
2.00
3.03
1.96
1.20
1.20
1.11
f1 (ppm)

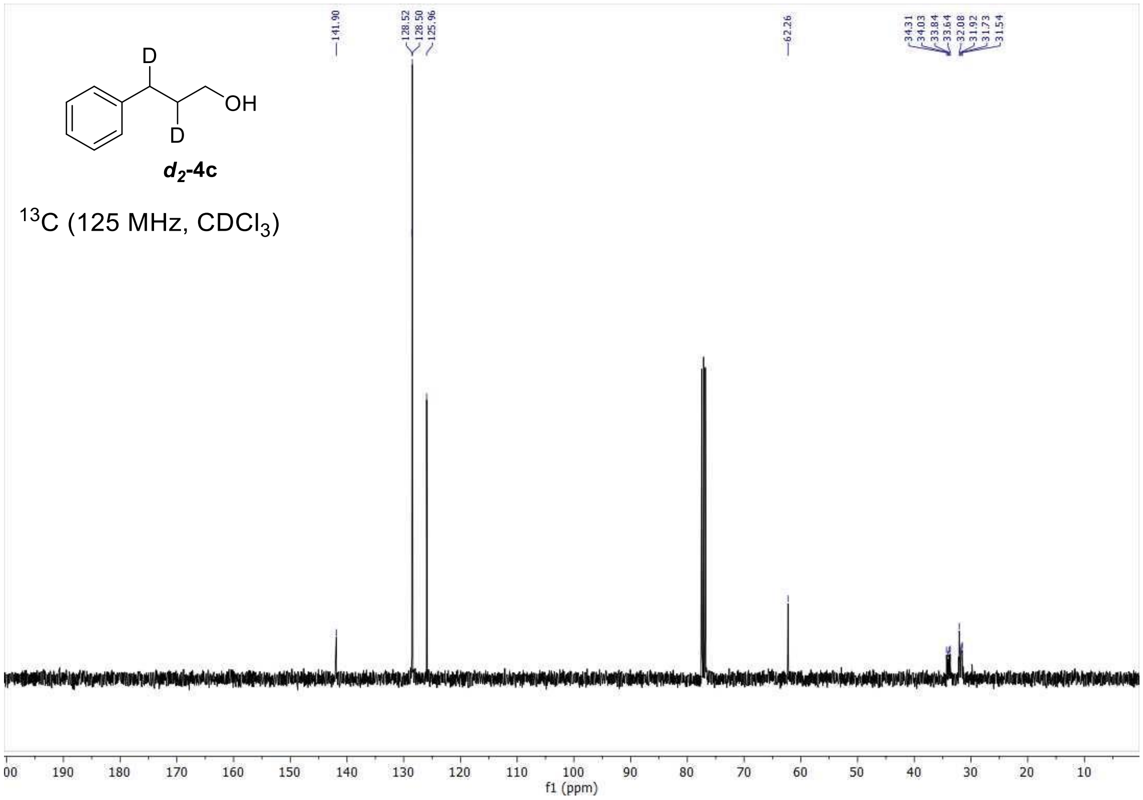

D
OH
D
d_2-4c
^{13}C (125 MHz, $CDCl_3$)
141.90
128.52
128.50
125.96
62.26
34.31
34.03
33.84
33.64
32.08
31.92
31.73
31.54
f1 (ppm)

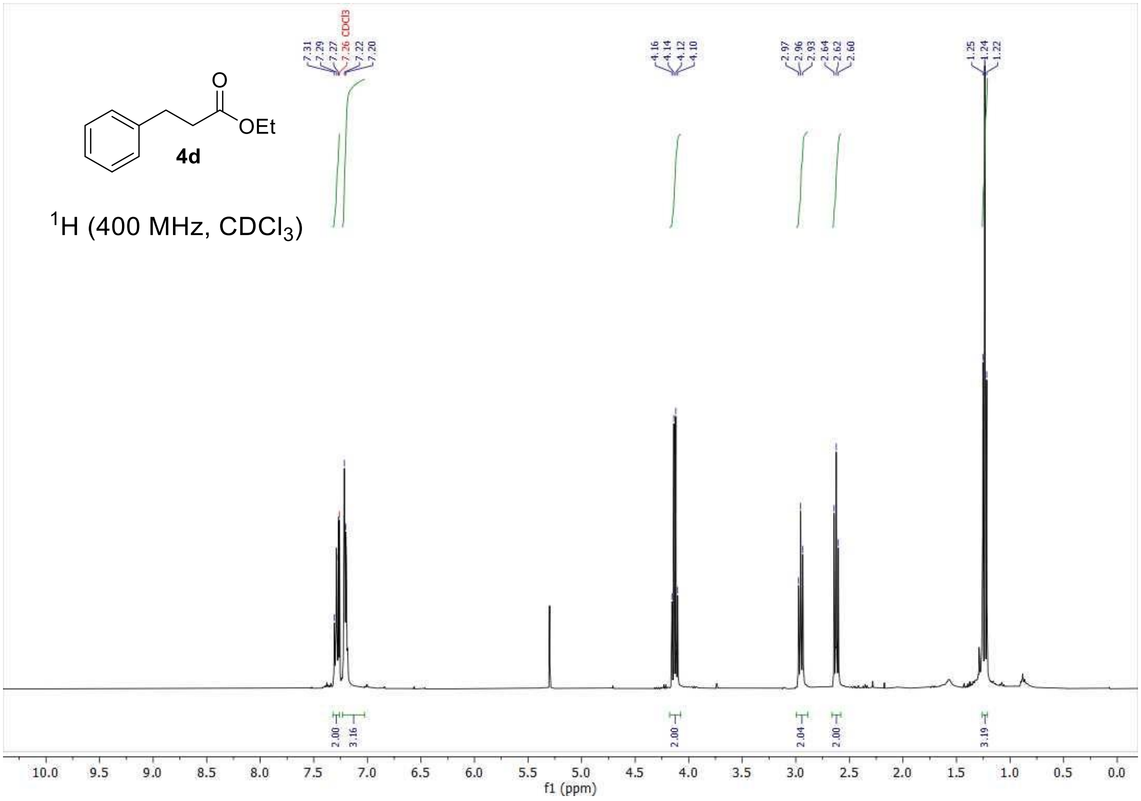
7.31 7.29 7.27 7.26 CDCl3 7.22 7.20
4.16 4.14 4.12 4.10
2.97 2.96 2.93 2.64 2.62 2.60
1.25 1.24 1.22
O
OEt
4d
1H (400 MHz, CDCl3)
2.00 3.16 2.00 2.04 2.00 3.19
10.0 9.5 9.0 8.5 8.0 7.5 7.0 6.5 6.0 5.5 5.0 4.5 4.0 3.5 3.0 2.5 2.0 1.5 1.0 0.5 0.0
f1 (ppm)

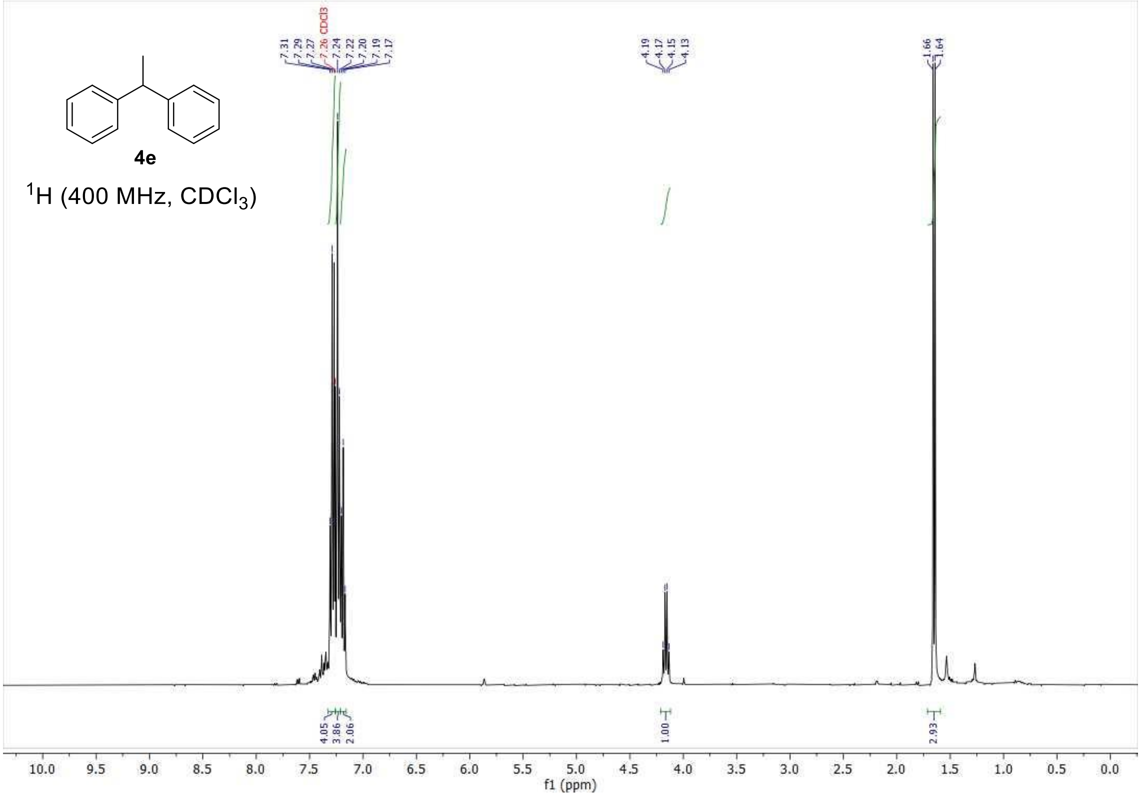
7.31 7.29 7.27 7.26 CDCl3 7.24 7.22 7.20 7.19 7.17
4.19 4.17 4.15 4.13
1.66 1.64
4e
1H (400 MHz, CDCl3)
4.05 3.86 2.06 1.00 2.93
10.0 9.5 9.0 8.5 8.0 7.5 7.0 6.5 6.0 5.5 5.0 4.5 4.0 3.5 3.0 2.5 2.0 1.5 1.0 0.5 0.0
f1 (ppm)

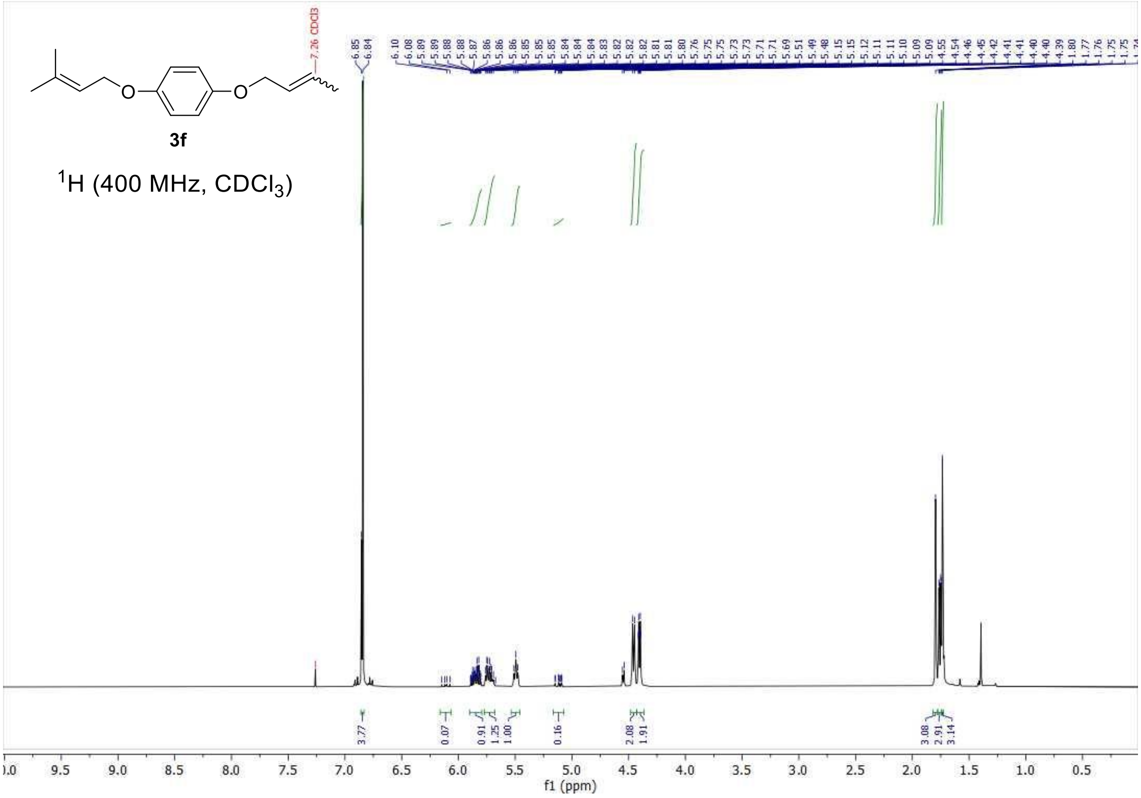
3f
1H (400 MHz, CDCl3)
7.26 CDCl3
f1 (ppm)

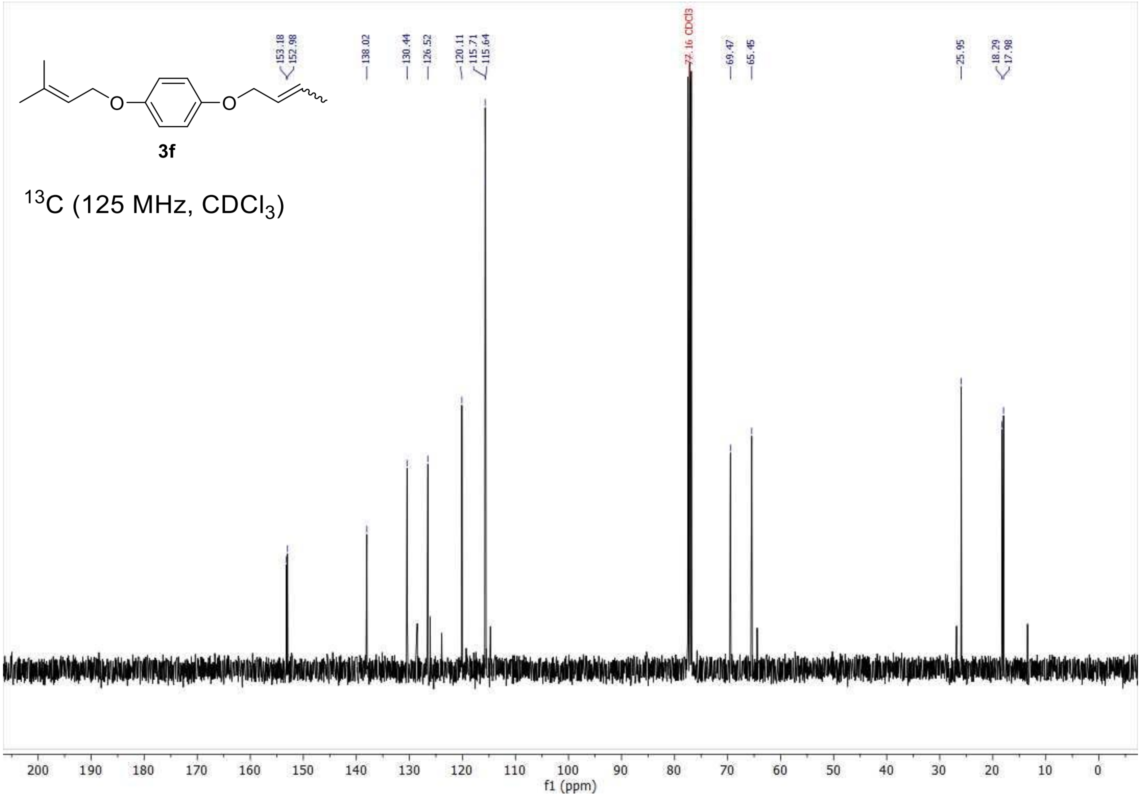
3f
13C (125 MHz, CDCl3)
153.18
152.98
138.02
130.44
126.52
120.11
115.71
115.64
77.16 CDCl3
69.47
65.45
25.95
18.29
17.98
f1 (ppm)

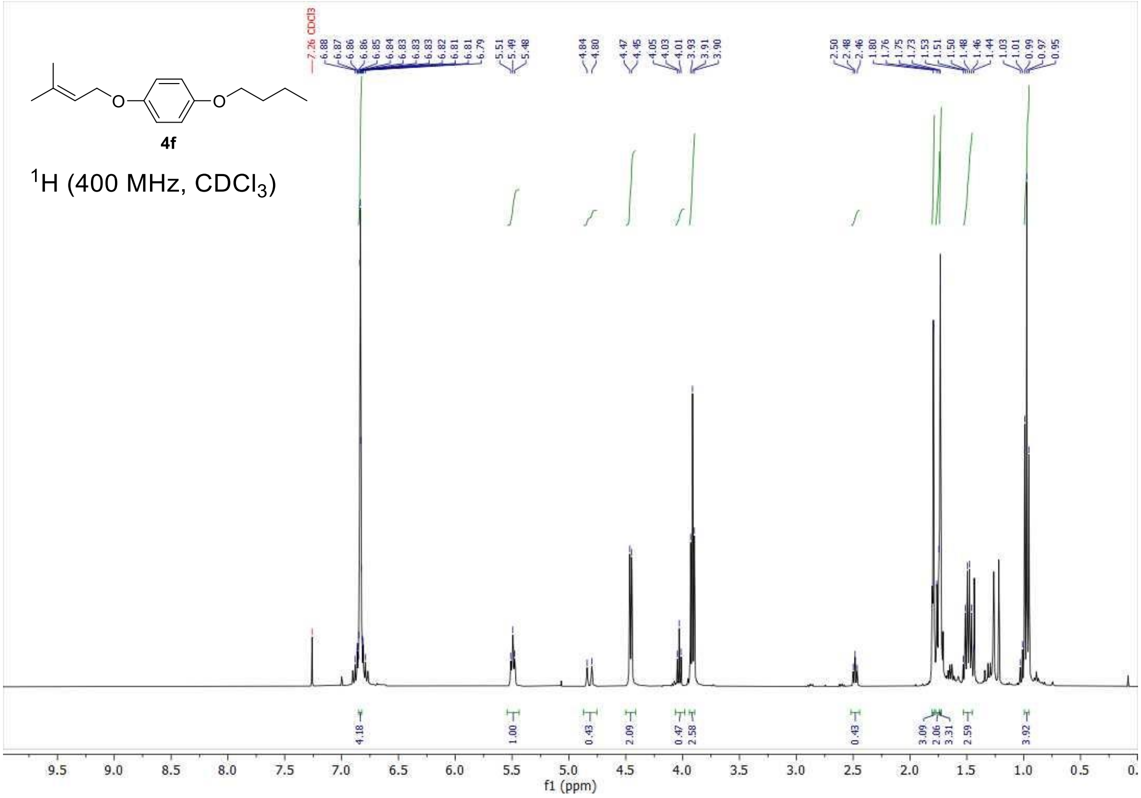
4f
1H (400 MHz, CDCl3)
7.26 CDCl3
f1 (ppm)

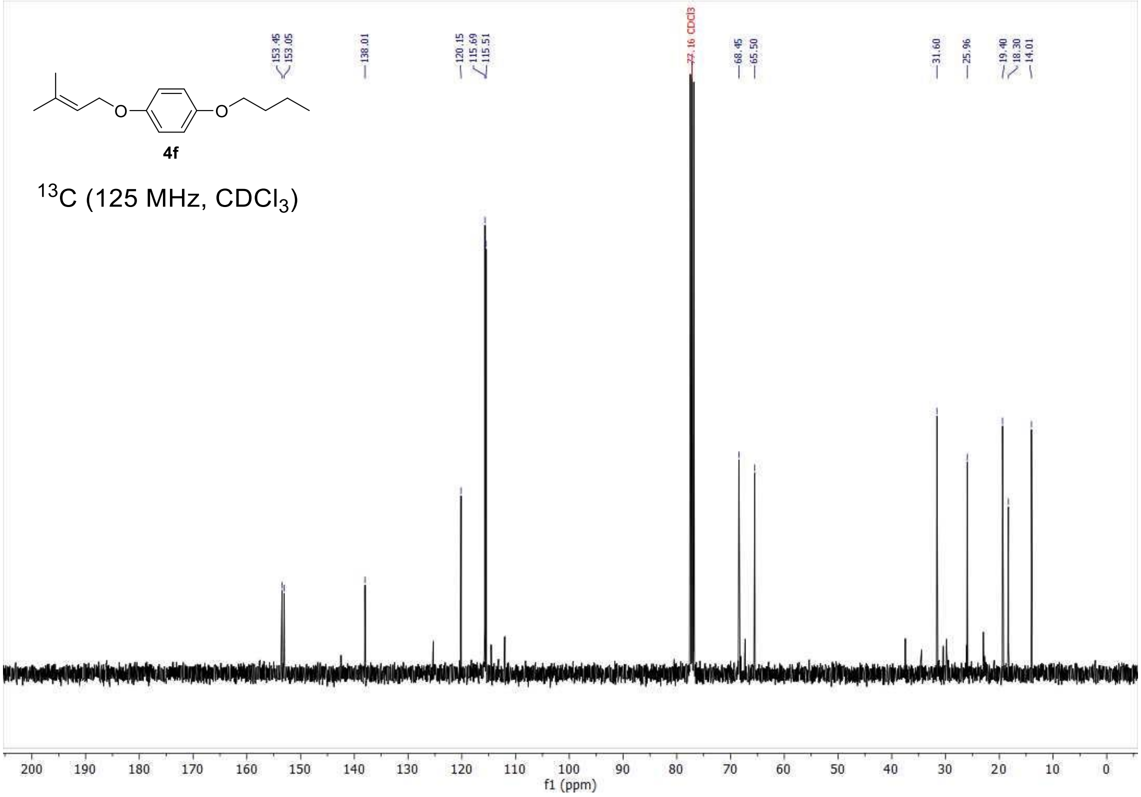
4f
13C (125 MHz, CDCl3)
77.16 CDCl3
f1 (ppm)

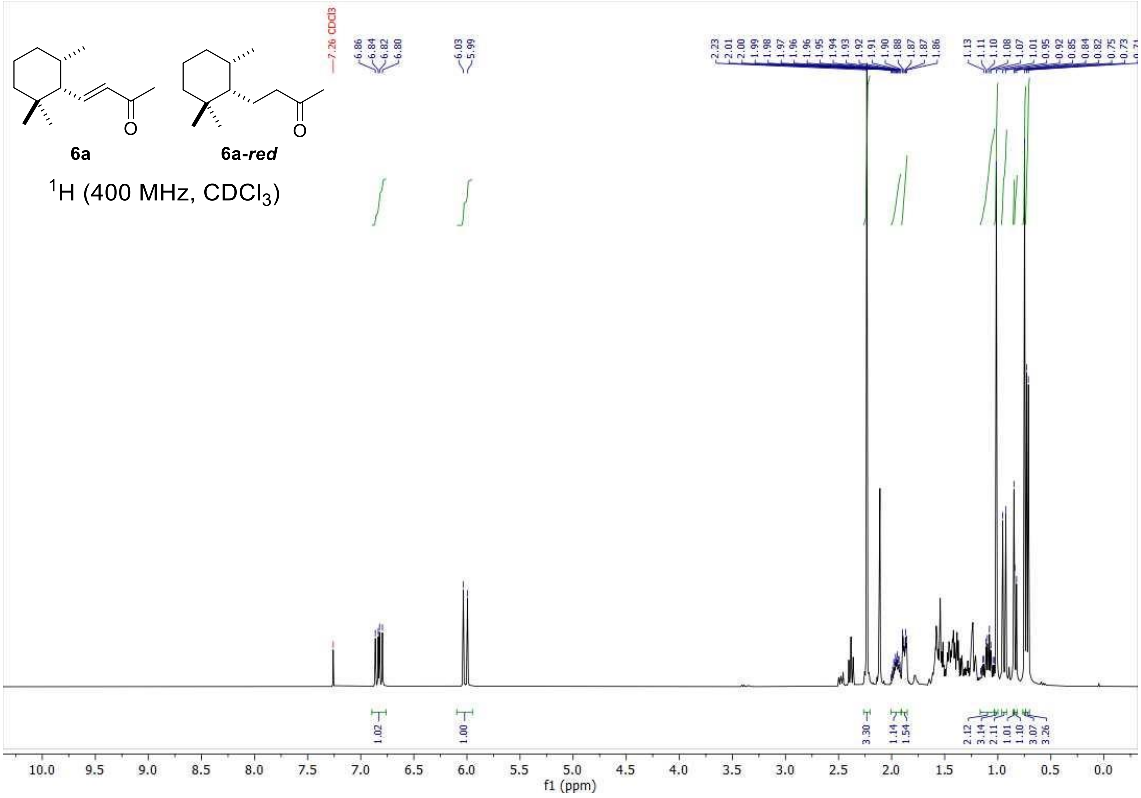
6a
6a-red
1H (400 MHz, CDCl3)
f1 (ppm)

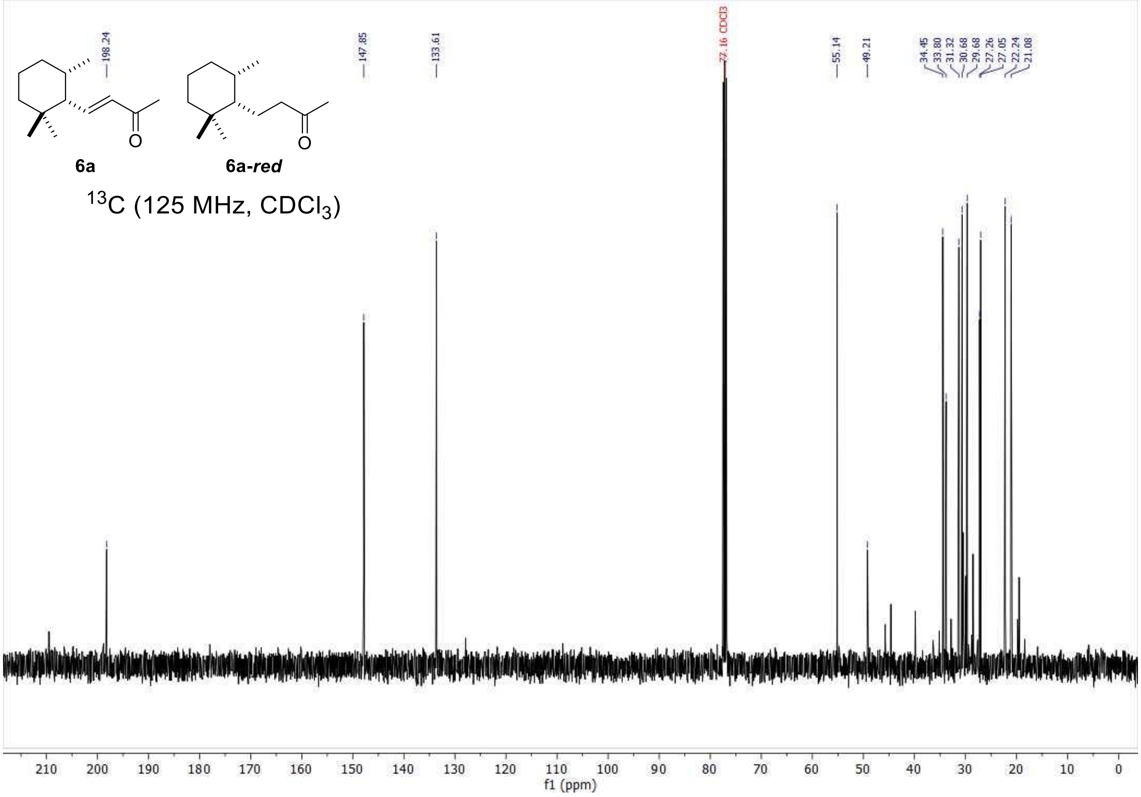
6a
6a-red
13C (125 MHz, CDCl3)
198.24
147.85
133.61
77.16 CDCl3
55.14
49.21
34.45
33.80
31.32
30.68
29.68
27.26
27.05
22.24
21.08
f1 (ppm)

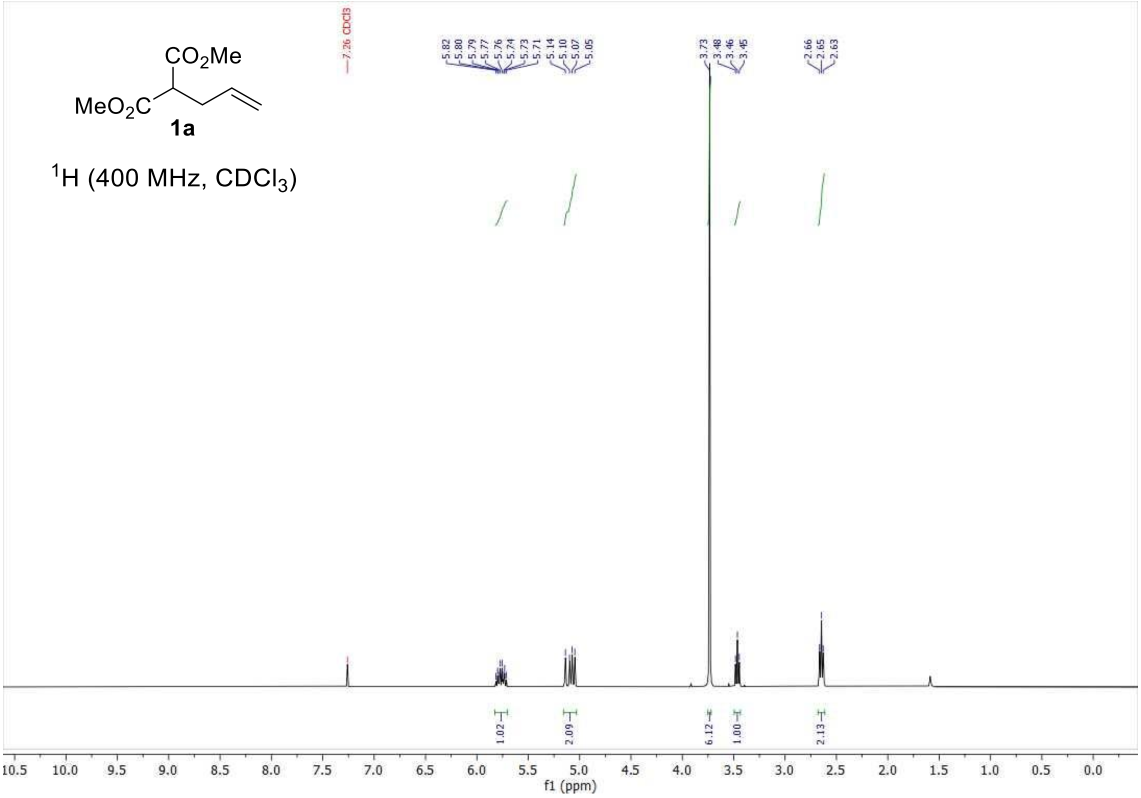
CO2Me
MeO2C
1a
1H (400 MHz, CDCl3)
f1 (ppm)

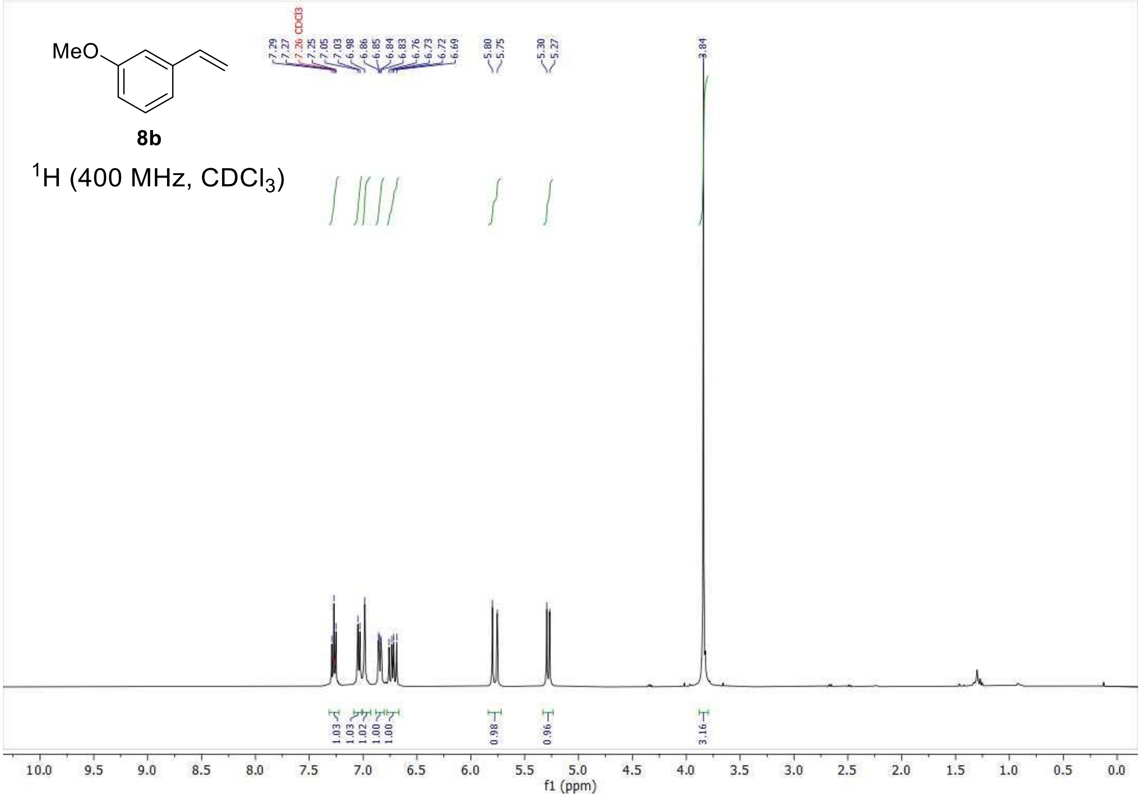
MeO
8b
1H (400 MHz, CDCl3)
f1 (ppm)

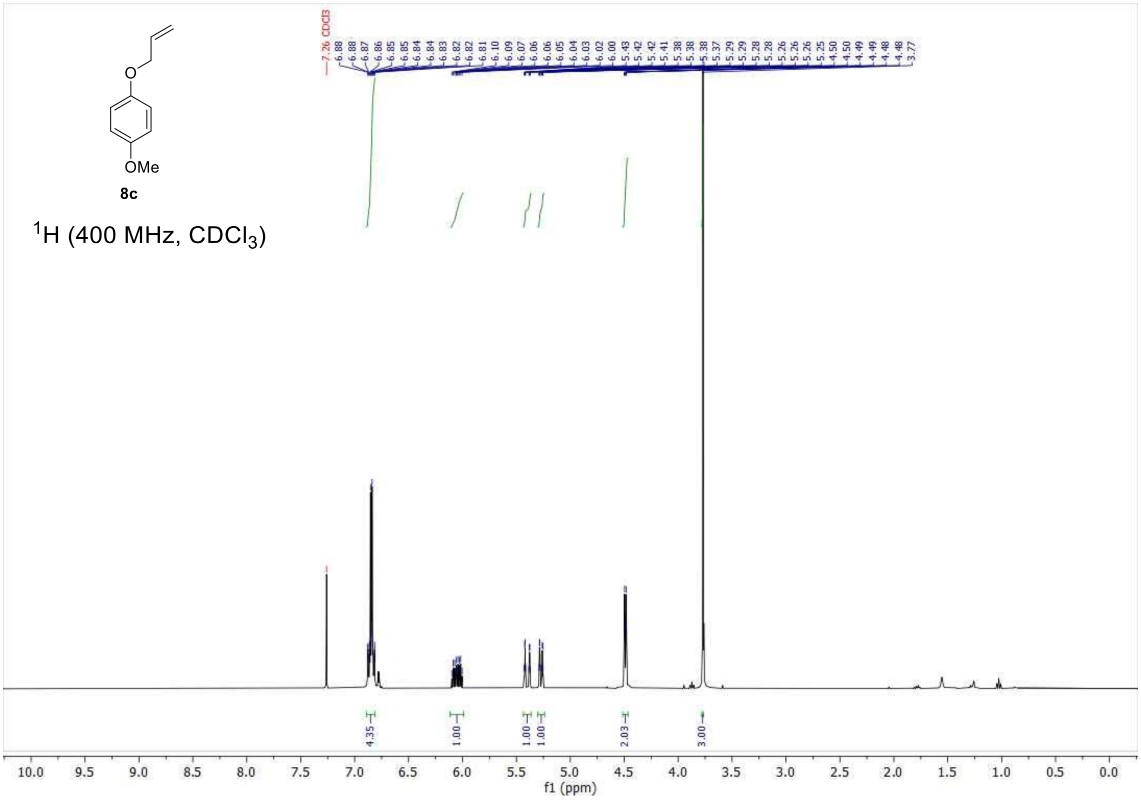
OMe
8c
1H (400 MHz, CDCl3)
7.26 CDCl3
6.88 6.87 6.86 6.85 6.85 6.84 6.84 6.83 6.82 6.82 6.81 6.10 6.09 6.07 6.06 6.06 6.05 6.04 6.03 6.02 6.00 5.43 5.42 5.41 5.38 5.38 5.38 5.37 5.29 5.29 5.28 5.28 5.26 5.26 5.26 5.25 4.50 4.49 4.49 4.48 4.48 3.77
4.35 1.00 1.00 1.00 2.03 3.00
10.0 9.5 9.0 8.5 8.0 7.5 7.0 6.5 6.0 5.5 5.0 4.5 4.0 3.5 3.0 2.5 2.0 1.5 1.0 0.5 0.0
f1 (ppm)

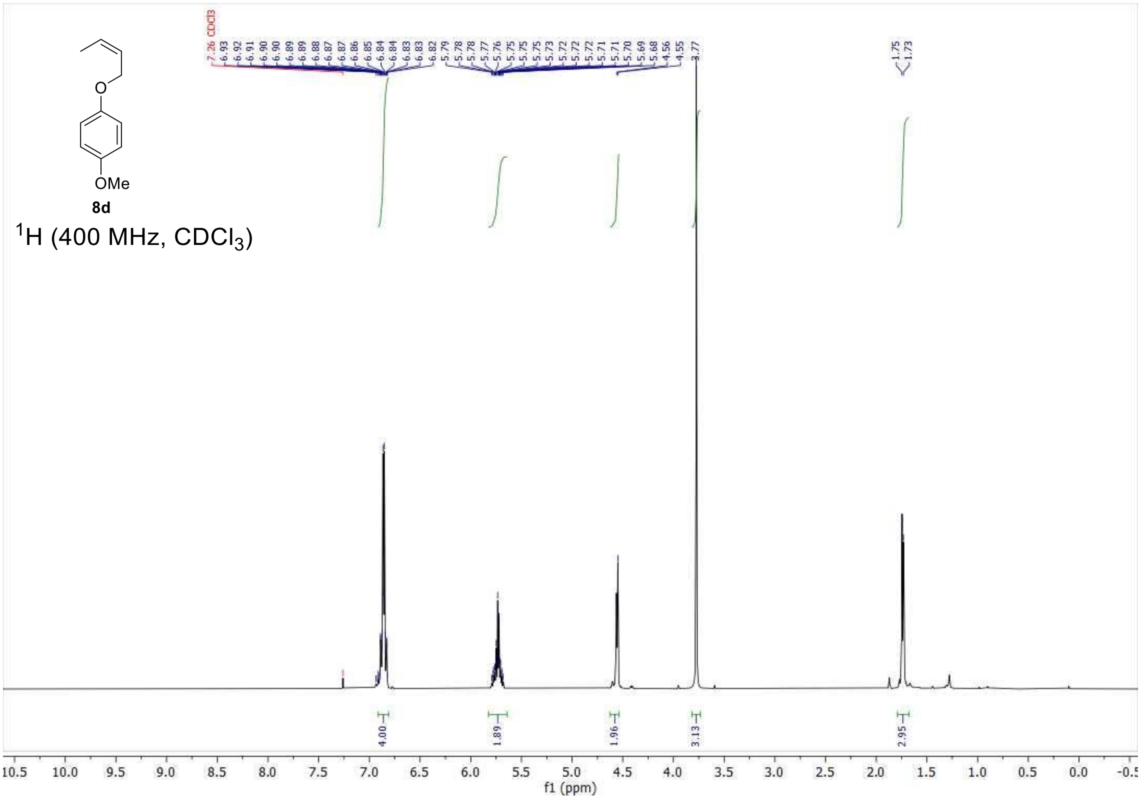

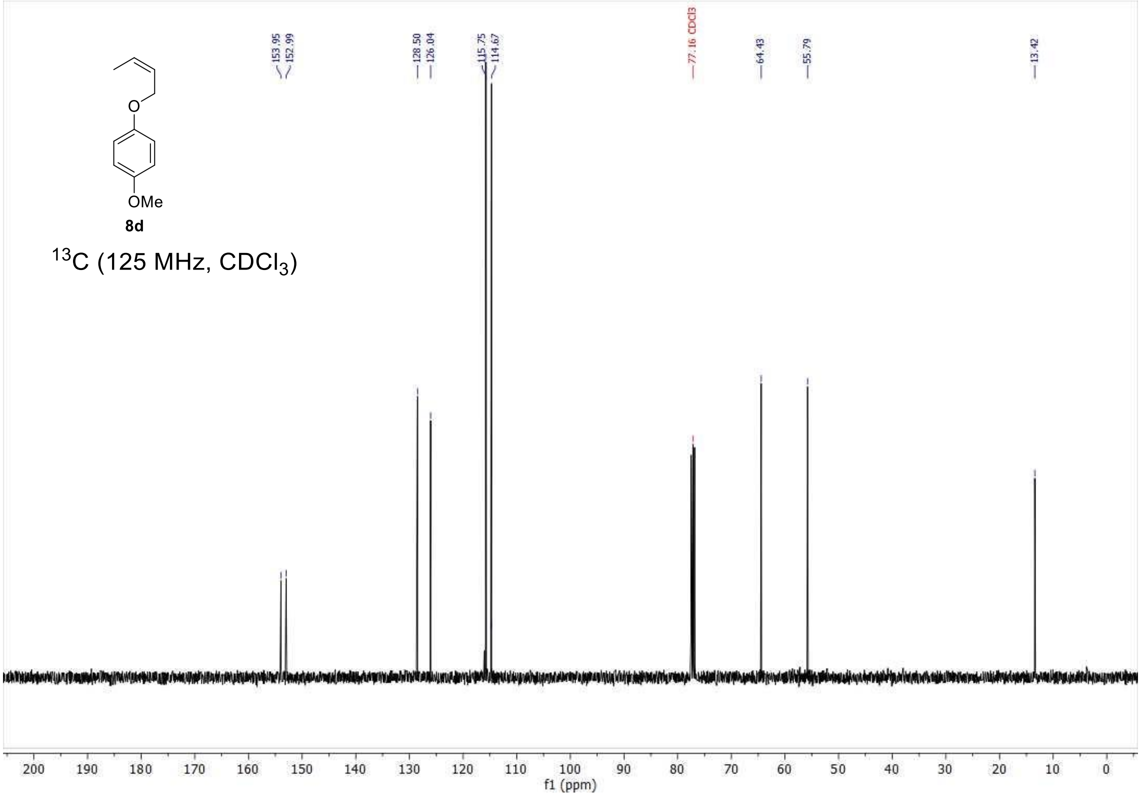

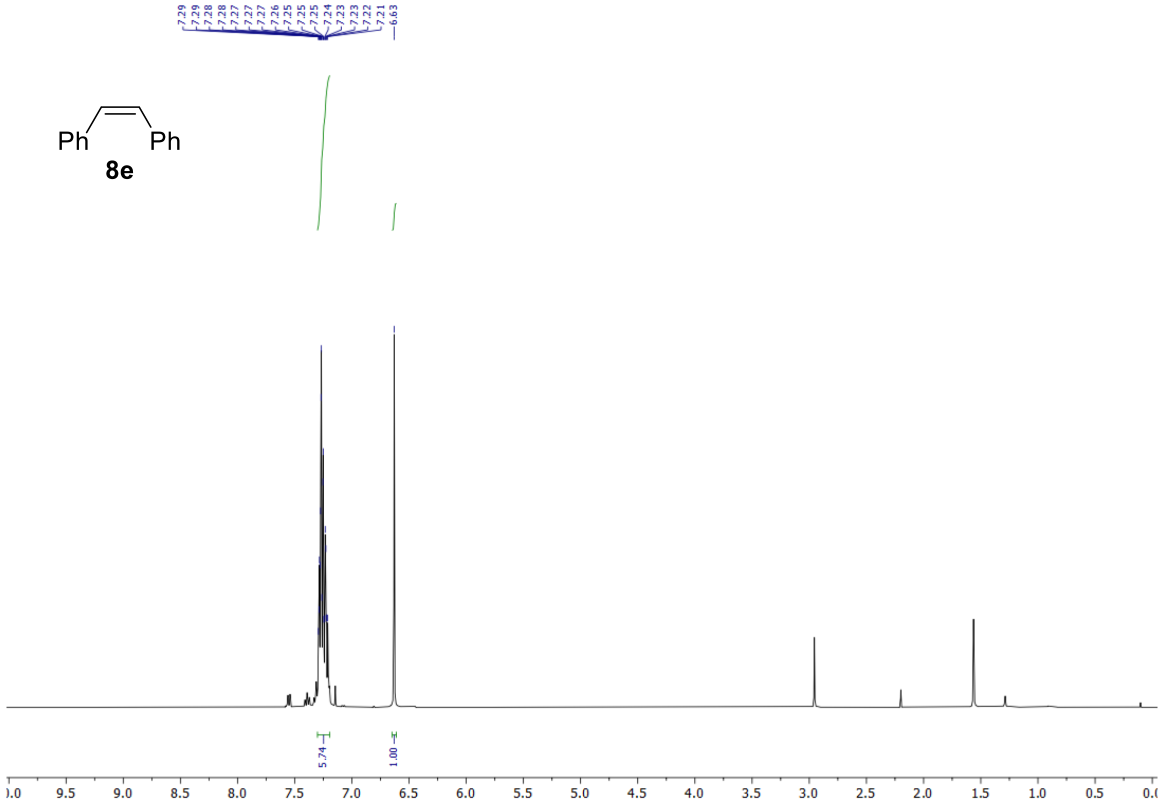

Supplement: Supplementary file 1 — ol3c03664_si_001.pdf [file ol3c03664_si_001.pdf]
